# Willingness to share contacts in case of COVID-19 positivity–predictors of collaboration resistance in a nation-wide Italian survey

**DOI:** 10.1371/journal.pone.0274902

**Published:** 2022-09-27

**Authors:** Boris Bikbov, Mauro Tettamanti, Alexander Bikbov, Barbara D’Avanzo, Alessia Antonella Galbussera, Alessandro Nobili, Gemma Calamandrei, Valentina Candini, Fabrizio Starace, Cristina Zarbo, Giovanni de Girolamo

**Affiliations:** 1 Dipartimento di Politiche per la Salute, Istituto di Ricerche Farmacologiche Mario Negri IRCCS, Milan, Italy; 2 Centre Maurice Halbwachs, École des hautes études en sciences socials, Paris, France; 3 Centro di Riferimento per le Scienze Comportamentali e la Salute Mentale, Istituto Superiore di Sanita, Rome, Italy; 4 Dipartimento di Salute Mentale, Azienda Unità Sanitaria Locale di Modena, Modena, Italy; 5 Unità di Psichiatria Epidemiologica a Valutativa, IRCCS Istituto Centro San Giovanni di Dio Fatebenefratelli, Brescia, Italy; University of Ferrara: Universita degli Studi di Ferrara, ITALY

## Abstract

**Background:**

The unwillingness to share contacts is one of the least explored aspects of the COVID-19 pandemic. Here we report the factors associated with resistance to collaborate on contact tracing, based on the results of a nation-wide survey conducted in Italy in January-March 2021.

**Methods and findings:**

The repeated cross-sectional on-line survey was conducted among 7,513 respondents (mean age 45.7, 50.4% women) selected to represent the Italian adult population 18–70 years old. Two groups were defined based on the direct question response expressing (1) unwillingness or (2) willingness to share the names of individuals with whom respondents had contact. We selected 70% of participants (training data set) to produce several multivariable binomial generalized linear models and estimated the proportion of variation explained by the model by McFadden R^2^, and the model’s discriminatory ability by the index of concordance. Then, we have validated the regression models using the remaining 30% of respondents (testing data set), and identified the best performing model by removing the variables based on their impact on the Akaike information criterion and then evaluating the model predictive accuracy. We also performed a sensitivity analysis using principal component analysis.

Overall, 5.5% of the respondents indicated that in case of positive SARS-CoV-2 test they would not share contacts. Of note, this percentage varied from 0.8% to 46.5% depending on the answers to other survey questions. From the 139 questions included in the multivariable analysis, the initial model proposed 20 independent factors that were reduced to the 6 factors with only modest changes in the model performance. The 6-variables model demonstrated good performance in the training (c-index 0.85 and McFadden R^2^ criteria 0.25) and in the testing data set (93.3% accuracy, AUC 0.78, sensitivity 30.4% and specificity 97.4%). The most influential factors related to unwillingness to share contacts were the lack of intention to perform the test in case of contact with a COVID-19 positive individual (OR 5.60, 95% CI 4.14 to 7.58, in a fully adjusted multivariable analysis), disagreement that the government should be allowed to force people into self-isolation (OR 1.79, 95% CI 1.12 to 2.84), disagreement with the national vaccination schedule (OR 2.63, 95% CI 1.86 to 3.69), not following to the preventive anti-COVID measures (OR 3.23, 95% CI 1.85 to 5.59), the absence of people in the immediate social environment who have been infected with COVID-19 (1.66, 95% CI 1.24 to 2.21), as well as difficulties in finding or understanding the information about the infection or related recommendations. A limitation of this study is the under-representation of persons not participating in internet-based surveys and some vulnerable groups like homeless people, persons with disabilities or migrants.

**Conclusions:**

Our analysis revealed several groups that expressed unwillingness to collaborate on contact tracing. The identified patterns may play a principal role not only in the COVID-19 epidemic but also be important for possible future public health threats, and appropriate interventions for their correction should be developed and ready for the implementation.

## Introduction

Collaboration is essential for successful control of COVID-19 epidemic, as well as other public health threats. Since the beginning of the pandemic there were examples of community and individual response that substantially influenced the spread of infection at the population level [1,2]. Some types of response have facilitated the epidemiological control (volunteering, reorientation of business to produce personal protective equipment, etc), while others have negatively affected anti-epidemic measures (fake news, negation of virus, etc). The individual beliefs, attitudes and behaviours have played a crucial role in the adherence to these measures. Among them, the willingness of individuals to share contacts in case of COVID-19 positivity plays a crucial role to secure effective contact tracing and subsequent quarantine of potentially infected persons [3–5]. Moreover, efficient contact tracing might be not only forward but both forward and backward, allowing the identification of unascertained individuals or asymptomatic carriers and thus securing the isolation of index cases who were involved in undiscovered previous transmission [6]. However, a certain proportion of individuals is not willing to cooperate with health and public authorities, and this phenomenon can be interpreted in the general context of distrust towards public institutions. Revealing the factors associated with individuals’ resistance to collaborate with contact tracing teams is important for targeting interventions which tackle this behaviour and increase the collaboration with health authorities in the current and possible future epidemics.

Timely testing leads to a substantial reduction of deaths, hospitalizations and even to the net cost-saving effect at the population level [7]. Among the pillars of anti-epidemic measures—i.e. testing, tracing, isolation, vaccination—the patterns of contact tracing that mainly depend on the readiness of infected persons to collaborate are not sufficiently studied. Of note, only appropriate contact tracing and screening might effectively identify and isolate presymptomatic or widely presented asymptomatic carriers who contribute to almost a half of all transmissions [8,9]. During the initial phase of the COVID-19 epidemic a successful contact tracing may reduce by more than 60% the number of new infections and deaths [10], and also in the modern stage of relaxation of physical distancing contact tracing may reduce the transmission rate by almost 50% [11]. This paper presents the results of a survey conducted in a representative sample of the Italian population and evaluates the demographic, social, and behavioural characteristics of persons who indicated that in case of positive SARS-CoV-2 test they would not share the names of individuals with whom they have had contacts. We also estimated the odds ratios (OR) of unwillingness to share contacts for different factors, produced several models predicting the unwillingness to share contacts, and validated these models.

## Methods

### Participants

This survey is a part of the project “Monitoring knowledge, risk perceptions, preventive behavior and trust to inform pandemic outbreak response” promoted by the WHO Regional Office for Europe. The survey study was conducted by Doxa S.p.a. by the CAWI technique (Computer Assisted Web Interviewing) on an online panel and on the Confirmit software platform used by Doxa S.p.a. In total, 7,513 respondents surveyed in 3 waves (January, February, March 2021) are included in the analyses. All participants received an invitation by e-mail to fill the online interview via a link: first, informed consent was requested and then the questionnaire was accessed. Participants freely decided to participate in the study, with no financial incentive. The average administration time was 20 min. A detailed sampling plan was developed to obtain a representative sample of the Italian adult population and achieve the generalisability of the study. The following variables were taken into account for the stratification of the participants: gender by age (four age groups: 18–34 years, 35–44 years, 45–54 years, 55–70 years), geographical area (four areas: North West, North East, Centre, South and Islands), size of living centers (two classes: above and below 100,000 inhabitants), level of education (up to lower middle school, beyond lower middle school), and employment situation (employed, not employed). The online questionnaire was administered in three waves by a survey company (BDA-Doxa) to a sample of participants representative to the national Italian population, weighted by aforementioned strata (gender, age, etc.). At the end of each survey’s wave, a weighting procedure was applied to accurately restore the proportionality of the total sample examined with the reference population, according to the data of the Italian Statistics Institute (ISTAT) updated to December 31^st^, 2019. In particular, data were weighted for the main socio-demographic and geographic variables (e.g., sex by age according to geographical area, occupation, educational qualification, geographical area by size of living centers). The sample size made it possible to maintain a sampling error of less than 2% (at the significance level of 95%) and to control the error of estimates within groups or subgroups of interest. Taking into account the similarity of numbers in both the original and the weighted samples, only unweighted results are reported here. This choice also facilitates the construction and interpretation of the regression models.

The parent project “Monitoring knowledge, risk perceptions, preventive behavior and trust to inform pandemic outbreak response” promoted by the WHO Regional Office for Europe has been approved by the Ethical Committee of the Italian coordinating institution (protocol 286/2020, registration ISRCTN 39724), and the current survey has approval from the Ethics Committee of the IRCCS San John of God Fatebenefratelli of Brescia (n° 72–2020). All survey participants provided written informed consent to participate in this study at the beginning of the web-based survey.

### COVID-19 epidemic during the survey

Italy was the first EU country heavily stroked by the COVID-19 in 2020. At the time of the survey implementation, the epidemiological situation had become more stable. Thus, in the mid-December 2020, none of the country regions were assigned as having the “red zone” status with the highest contamination rates, four regions were assigned by “orange zone”, and the remaining 16 regions by “yellow zone” status. However, during February-March 2021, some provinces and regions have transferred back to “orange” or even “red” zone status. In the considered period anti-epidemic measures (mask use, distancing, etc.) were highly recommended by the official bodies and controlled by police, employers and public places personnel. The vaccination campaign was officially started on 27^th^ December 2020, with administration of vaccine during the conduction of the survey mainly to health professionals, elderly persons, or fragile patients. Additional restrictions were introduced during the second COVID-19 wave in October 2020 and then around the Christmas holidays, but they were gradually eased in the first trimester of 2021. The number of new confirmed daily cases in Italy substantially varied during the survey conduction, accounting for about 15,000 in the first half of the January 2021, about 12,000 in the second half of the January and the first half of the February, and then increased to about 22,000 daily cases in March. Daily COVID-19 deaths were close to 500 at the beginning of January, then slowly decreased to about 300 in mid-February, and after increased again to about 430 daily deaths at the end of March. On 8^th^ March 2021 Italy passed the grim threshold of 100,000 COVID-19 deaths that has a substantial media effect.

Contact tracing was introduced by the Ministry of Health ordinance in March 2020 to reveal persons with whom the infected individual has a strict contact up to 48 hours before and until the 14 days after the positive test. For citizens the contact reporting was highly recommended by Italian law, but it was voluntary and not covered by any punishment or reward. Strict contact persons should follow the 14-day home quarantine starting from the date of the latest exposure. The Italian National Social Security Institute has defined separate rules for the insurance coverage of quarantine days for employers involved in different professional activities. In 2020 it covered the quarantine days spent by employed individuals having a contract with public or private organisations and did not cover it for self-employed individuals. Since the January 2021, this insurance for quarantine was provided only to workers who had a contract and could not execute professional activity remotely.

### Statistical analysis

We performed a cross-sectional analysis of all survey participants, with the principal division into two groups of those who, in case they were positive to SARS-CoV-2 test, express (1) unwillingness or (2) willingness to share the names of individuals with whom they had contact. We produced descriptive statistics for these two groups, and evaluated whether they had differences in demographic and social characteristics, beliefs and behavior. Fisher exact test or χ^2^-test was used to estimate the difference in contingency tables.

To reveal the independent predictors of unwillingness to share the contacts we randomly selected 70% of participants (training data set) to produce the regression models. For this purpose we selected the variables with a statistical difference less than 0.2 obtained in χ^2^-test for the inclusion in the multivariable binomial generalized linear models (GLM) with forward and backward selection. Both models revealed the same core set of independent factors, and the model with forward selection has been chosen based on the better residual deviance value in the ANOVA analysis. We used McFadden R^2^ as a measure of the proportion of variation explained by the model, and the index of concordance (c-index) as a measure of the model’s discriminatory ability.

The resulting multivariable GLM model selected a quite high number of independent predictors and some of them introduced collinearity because they were related to the same domain of COVID-19 perception reported by the respondents. To control for overfitting, we have validated the regression models using the remaining 30% of participants (testing data set). Considering the substantial class imbalance with only a minority respondents reporting the parameter of interest (unwillingness to share contacts), we have evaluated different discrimination thresholds (from 0.1 to 0.5) for classification of testing data set according to the predicted probability of reporting the parameter of interest. Taking into account the area under the curve (AUC), classification accuracy, sensitivity and specificity (S1 Fig in S1 File), the optimal discrimination probability threshold was defined as 0.25, and it was used in the evaluation of all models on the testing data set. To improve the model and eliminate the variables that introduced substantial collinearity, we have subsequently removed from the regression model the variables one-by-one considering their impact to the Akaike information criterion (AIC), and evaluated after each iteration the model residual deviance and AIC in the training data set, and the predictive accuracy of the new models in the testing data set. Finally, we kept in the resulting multivariable model only a core set of predictors that demonstrated highest predictive accuracy in the testing data set.

We also performed a sensitivity analysis using principal component (PC) analysis, obtaining PCs explaining at least 60% of the variance for each specific domain. The extracted PCs were then inserted into multivariate logistic regression analyses to find if they were associated with the willingness/unwillingness to share the names of their contacts (see details in S1 File).

P < 0.05 in two-sided test was considered statistically significant. All analyses were performed using R v. 4.1.1 (R Foundation for Statistical Computing, Vienna, Austria).

## Results

### Prevalence of unwillingness to share contacts

Overall, 5.5% (416) of the 7,513 respondents responded that in case of positive SARS-CoV-2 test they would not share the names of people with whom they had contact. This percentage did not differ between three survey waves (6.3% in the first wave, 5.1% in the second and third waves, p = 0.09).

The survey contained a direct question (responded by all 416 persons) why a person opts for this choice. The most frequent reasons (the sum exceeds 100% because of multiple answers) were thoughts that sharing names could cause inconvenience to others (42.1%), wish to communicate directly with a contact person (40.1%), distrust in authorities (29.3%), fear of income loss in case of quarantine (25.5%), fear of personal vengeance from contact persons (21.4%), belief that family or friends expect their names will not be shared (15.4%), and unwillingness that others will know about a positive result (11.1%).

### Factors associated with unwillingness to share contacts

Principal demographic and social characteristics of participants are displayed in Table 1, and extended description of all survey parameters is presented in S1 Table in S1 File. Sex, education level, and employment status have not influenced, and age only marginally influenced on the decision to share contacts. Even among health professionals, the percentage did not dramatically differ from the general population (3.8% and 5.5%, p = 0.17).

**Table 1:** Demographic and social characteristics of the survey participants (n = 7513) according to the attitude for willingness to share contacts in case of positivity.

Survey parameter	Total *	Willingness to share contacts in case of positivity **		p
		Would share	Would not share	
**Age, years**	45.7 (12.9)	45.9 (12.8)	43.5 (12.9)	<0.001
**Age group**				
18–34 years	1945 (25.9)	1814 (93.3)	131 (6.7)	0.011
35–44 years	1449 (19.3)	1365 (94.2)	84 (5.8)	
45–54 years	1783 (23.7)	1685 (94.5)	98 (5.5)	
55–70 years	2336 (31.1)	2233 (95.6)	103 (4.4)	
**Sex**				
Male	3724 (49.6)	3506 (94.1)	218 (5.9)	0.254
Female	3789 (50.4)	3591 (94.8)	198 (5.2)	
**Education**				
elementary school / junior high school	3078 (41.0)	2905 (94.4)	173 (5.6)	0.354
high school	2495 (33.2)	2369 (94.9)	126 (5.1)	
degree or more	1940 (25.8)	1823 (94.0)	117 (6.0)	
**Employment**				
Yes	3940 (52.4)	3732 (94.7)	208 (5.3)	0.329
No	3573 (47.6)	3365 (94.2)	208 (5.8)	
**Settlement type**				
Rural/suburban zone (up to 100.000 inhabitants)	5746 (76.5)	5444 (94.7)	302 (5.3)	0.063
Urban zone (more than 100.000 inhabitants)	1767 (23.5)	1653 (93.5)	114 (6.5)	

Fisher exact test or χ^2^-test was used to estimate the difference in contingency tables. T-test was used to estimate the difference in age. Numbers in brackets indicate a percentage of respondents within each group, except the age value where standard deviation is reports in brackets.

* percentage refers to columns total;

** percentage refers to rows total.

#### Personal perception of pandemic

Personal experience with COVID-19 infection influenced on the decision to share contacts, making unwillingness lower among respondents who have already had COVID-19 infection confirmed by test, and higher among those who responded their infection was not confirmed by test or those who do not know whether they have been infected or not (3.7%, 10.5% and 10.1%, respectively, p<0.0001). The presence of people in the immediate social environment who are or have been infected with COVID-19 (both in suspected or confirmed forms) has substantially reduced unwillingness to share contacts to 4.4% compared to 8.6% among individuals who did not know about COVID-19 cases in close friends or relatives (p<0.0001).

**Fig 1:**
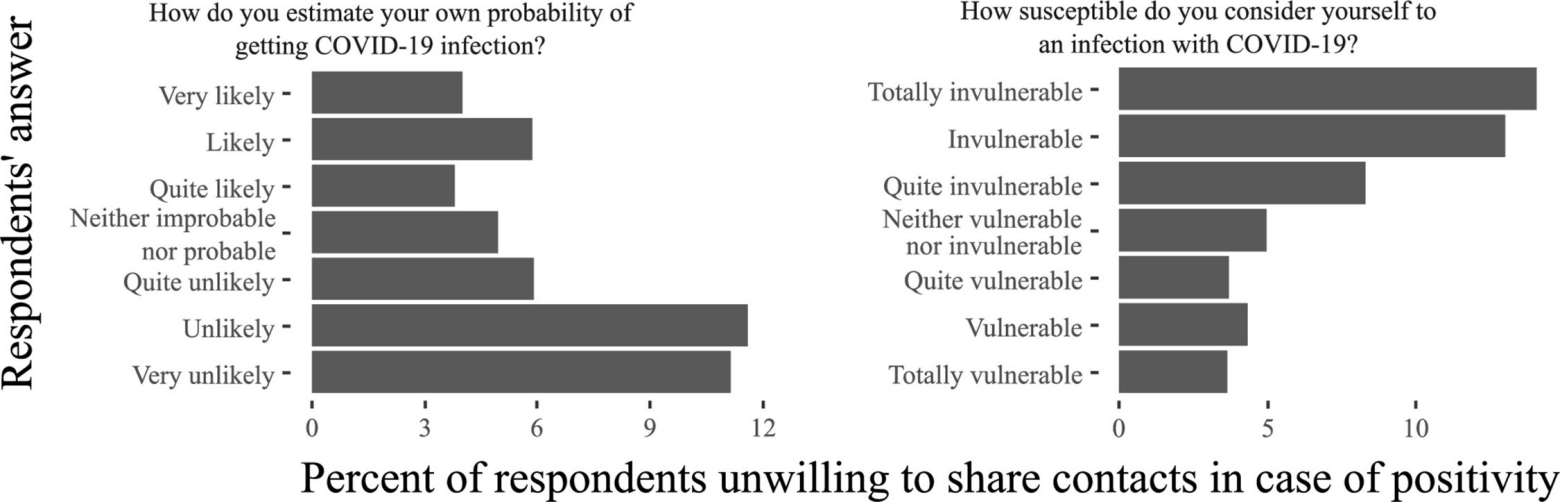
Percent of respondents who expressed unwillingness to share contacts in case of positivity, according to their response to the questions about personal probability of being infected.

Self-perception of high probability of getting COVID-19, high susceptibility or possible severe infection were related to willingness to share contacts (Fig 1), with the severity of the infection providing the most prominent difference (3.9% and 17.2% [p<0.0001] supposing it could be “very severe” and “not at all severe”, respectively). General perception of pandemic situation also played a role, and resistance to share was reported only by 2.5% of persons feeling COVID-19 is “close to her/him” compared to 12.2% feeling it is “far from her/him” (p<0.0001), with similar rates among acknowledging that pandemic is rising high or low. Respondents who reported to think frequently about pandemic were more prone to share contacts, as well as those who think that the pandemic makes a person vulnerable, is stressful or scary (2.1% vs 21.6% indicating it is scary or not, respectively, p<0.0001). Individuals who indicated that they strongly agree or disagree to have had a fast or easy recovery from stress during the pandemic tended to have higher unwillingness rates compared to those who expressed less contrasting opinions (p<0.0005), while general susceptibility to stressful events did not influence this decision (p = 0.14).

Unexpectedly, a small proportion of 2.5% of respondents who strongly disagreed of being worried about future economic consequences related to the pandemic (and thus having more optimistic view on personal economic perspectives) were less likely to share contacts (13.3% vs 4–6% in groups expressing other opinions, p<0.0001). Also, a small subgroup of 3.5% of participants who reported to feel cheerful and in good spirits all the time and a subgroup of 7.0% of participants who reported never feel cheerful had demonstrated higher unwillingness to share contacts as 10.2% and 7.0%, respectively, compared to other respondents (p<0.0001). Similarly, in a small subgroup of about 4% of respondents, unwillingness was much higher and reached almost 10.0% in subgroups of those who felt calm and relaxed all the time, or always felt active and vigorous, or always woke up feeling fresh and rested, or always felt the life filled with interesting things during the last 2 weeks.

During the pandemic some respondents have changed their habits and reported to exercise less, to eat more unhealthy food, to smoke or drink more alcohol–but this does not seem to have a substantial correlation with the intention to share contacts. However, a relatively minor group of about 15% of respondents who indicated that changes in these habits were not applicable to them, demonstrated higher rates of unwillingness to share contacts (thus, the percentage of unwillingness was about 5% in those who agreed or disagreed they eat more unhealthy food than before the pandemic, while it achieved 11.5% in those indicating this was not applicable to them, p<0.0001). Similarly rates were observed among respondents who indicated “not applicable” for the question whether they avoided going to a doctor for non-COVID reasons. To explore this unexpected phenomenon, we have evaluated the relationship with other questions and found that persons who responded “not applicable” also have substantially higher rates of reporting difficulties in finding and understanding the COVID-19 related information, understanding how to protect themselves from the infection and difficulties in avoiding infection (20%-30% respondents who answered “very difficult” on these domains indicated “not applicable” for changes in lifestyle compared to 4%-6% of those who have less or no difficulties), and lower trust in health authorities, lower adherence to wearing mask and physical distancing (10%-30% reporting lower compared to 4%-6% reporting higher levels, respectively).

Overt adherence to conspiracy theories that could be identified in 7–20% of respondents based on the estimation of different statements (like "There are secret organisations that greatly influence political decisions") was related to 2-3-fold higher unwillingness to share contacts compared to people who expressed milder agreement or disagreed with them.

#### COVID-19-specific information use

**Fig 2:**
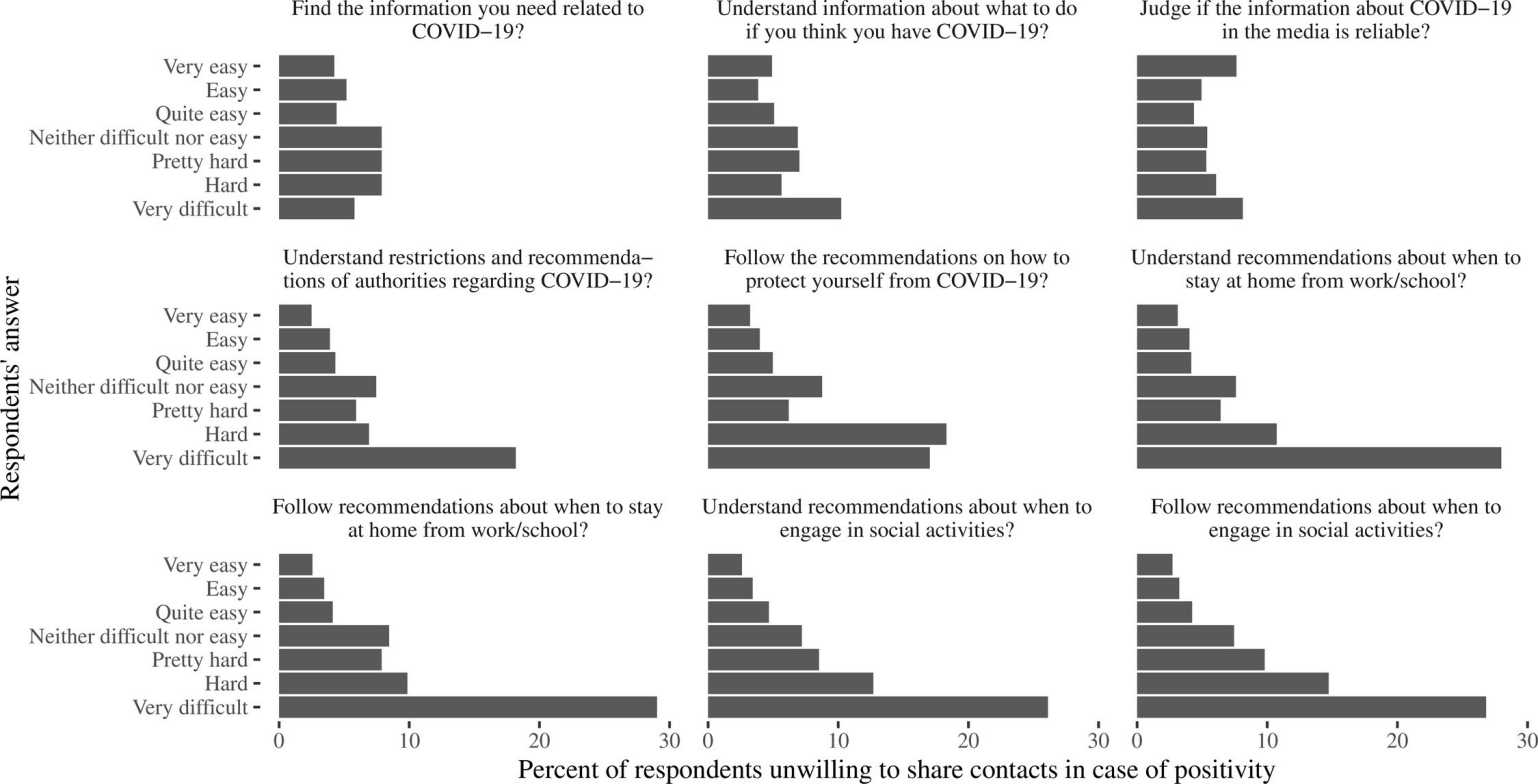
Percent of respondents who expressed unwillingness to share contacts in case of positivity, according to their response to the questions “How easy or hard it was to …”. The questions are named at each panel on the figure.

Patterns of information perception defined the people’s collaboration behavior. Importantly, a substantial proportion of the population reported difficulties in finding the COVID-19 related information (reported by 11.6%), understanding what to do in case of infection (19.0%), judging whether the information about COVID-19 in the media is reliable (38.6%), understanding restrictions and recommendations of authorities (23.8%), and these percentages were similar among respondents with any education level. All of these difficulties increased the unwillingness to share contacts (Fig 2), with a highest proportion of 18.2% among individuals indicating that understanding restrictions and recommendations of authorities was very difficult and lowest of 2.5% responding it was very easy (p<0.0001). Difficulties in practical implementation of these recommendations were reported by 10–15% of participants, with the most prominent difference in unwillingness between 29.0% and 2.6%, respectively, (p<0.0001) among reported that following the recommendations about when to stay at home was very difficult or very easy, and very similar rates in those reporting difficulties in understanding and following recommendations about when to not engage in social activities.

**Fig 3:**
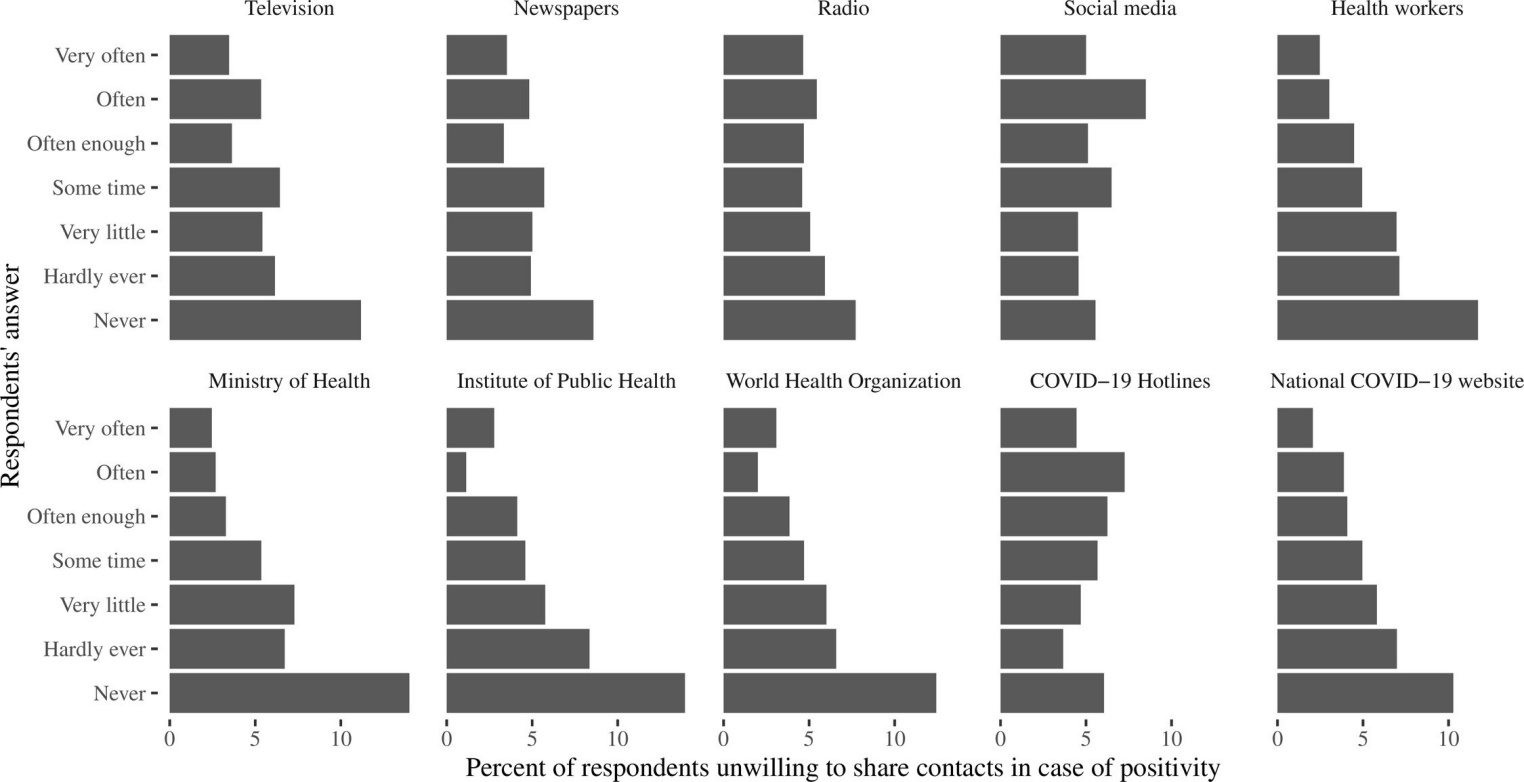
Percent of respondents who expressed unwillingness to share contacts in case of positivity, according to their response to the questions “How often you use COVID-19 related information from the following sources”. The sources of information are named at each panel on the figure.

More frequent use of mass media for obtaining COVID-19 related information was positively correlated to willingness to share contacts (Fig 3), with a little bit higher influence of television than newspapers or radio, and non-linear correlation with the use of COVID-19 hotlines, social media and information from famous persons. The highest influence had frequent information received from health authorities (health workers, Ministry of Health, Center for Disease Control, WHO, national COVID-19 information website) with a similar proportions in unwillingness to share contacts in 2–3% and 10–14% of persons receiving from them information “very frequently” and “never”. The frequency with which a person search information about COVID-19 positively correlated with willingness to share contacts, with about 3% unwilling to share in groups reported frequent search instead of 15.1% among those indicated never searched this information (p<0.0001). Of note, the proportion of respondents who never searched for the COVID-19 related information reached 6.1%, almost never– 8.1%, and very seldom– 11.4%.

**Fig 4:**
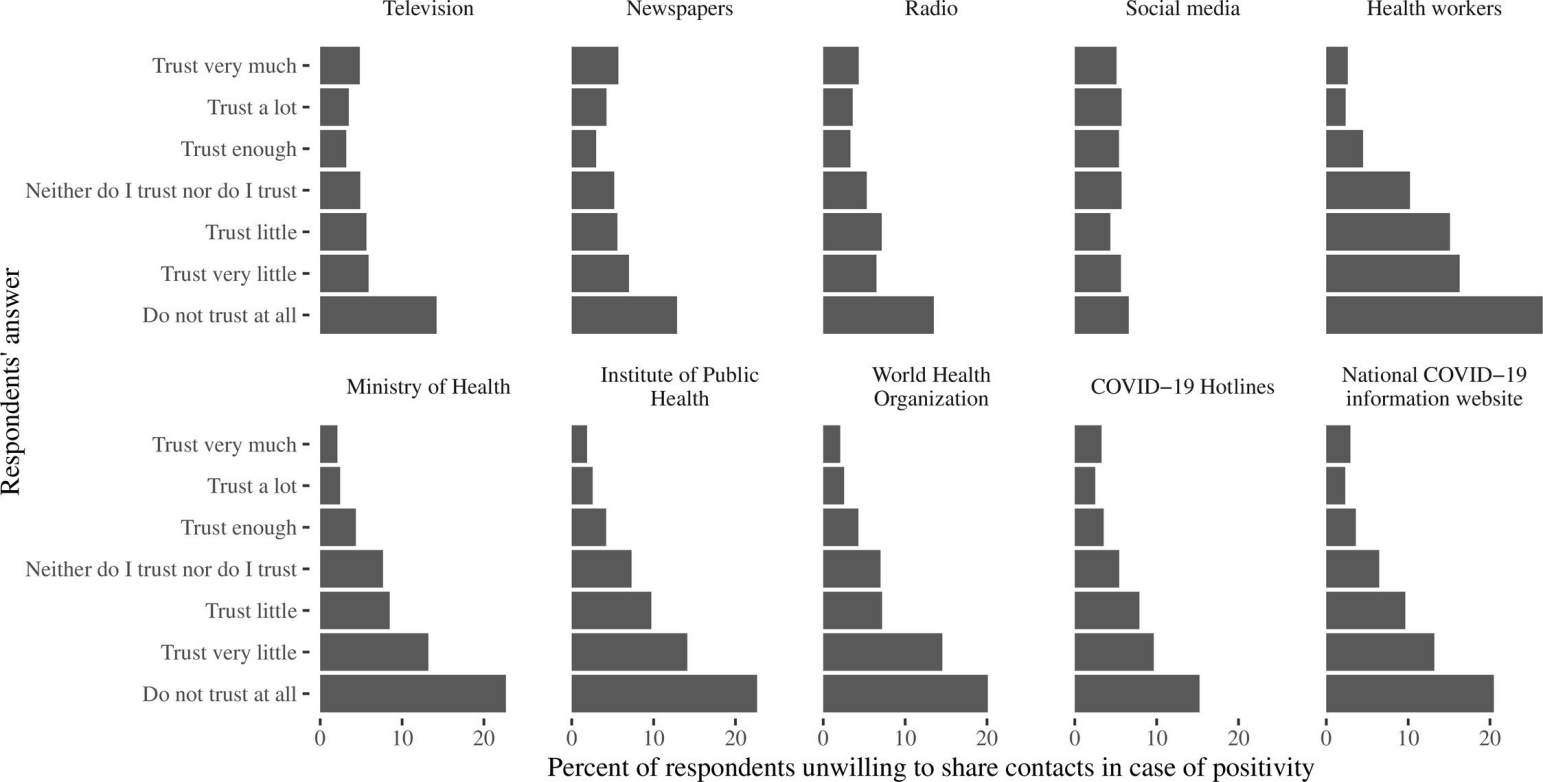
Percent of respondents who expressed unwillingness to share contacts in case of positivity, according to their response to the questions “How much you trust to the information about the COVID-19 supplied by the following sources”. The sources of information are named at each panel on the figure.

**Fig 5:**
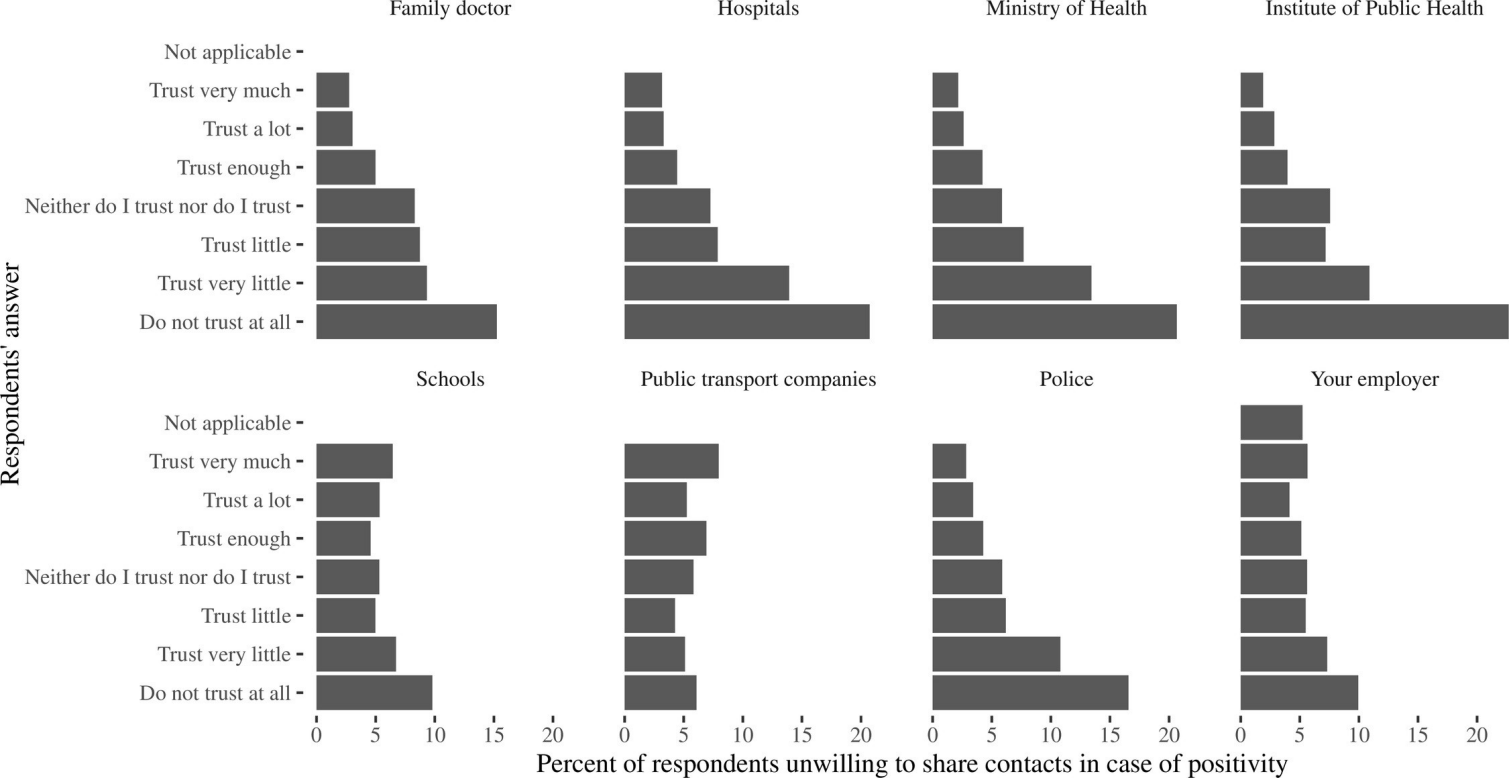
Percent of respondents who expressed unwillingness to share contacts in case of positivity, according to their response to the questions “How much trust you have that the following institutions or persons can effectively manage the COVID-19?”. The institutions are named at each panel on the figure.

Even more important was the trust in information (Fig 4) received from television, newspapers or radio (contacts would not be shared by 4–6% and 13–14% of persons trusting a lot and not trusting at all, respectively), and from health authorities (2–3% and 15–26%, respectively, based on the concrete authorities representative, see S1 Table in S1 File for exact numbers). However, this did not concern social media (5.1% and 6.7%, respectively, p = 0.22) and famous persons (unwillingness was J-shaped with more frequent percentage as 7.1–7.7% in both very trusting and very distrusting to them, p<0.01). The confidence of respondents in ability to manage the epidemic (Fig 5) by health authorities and police correlated with willingness to share contacts in case of a possible infection, and a percentage of people who would not like to share contacts was close to 2–3% among those who “trust a lot” compared to 15–20% in those who “do not have any trust”, while the confidence in the ability of other non-medical public institutions (employers, church, schools) to manage the epidemic had less prominent effect.

#### Adherence to anti-epidemic measures

**Fig 6:**
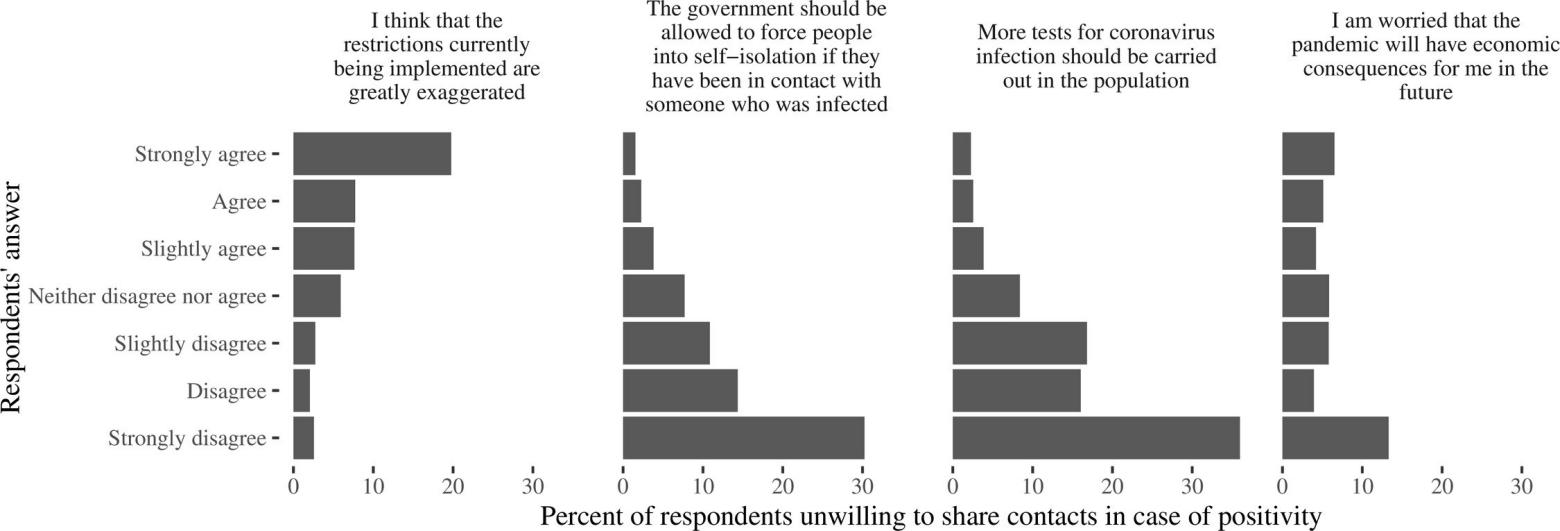
Percent of respondents who expressed unwillingness to share contacts in case of positivity, according to their response to the questions “Please express your opinion about the following phrases”. The phrases are named at each panel on the figure.

**Fig 7:**
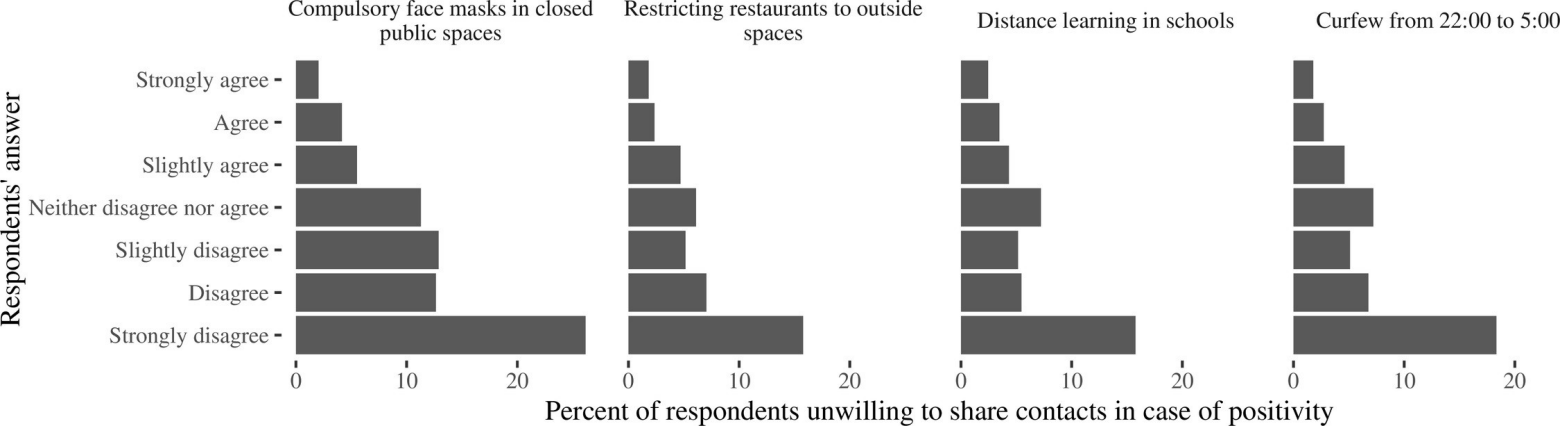
Percent of respondents who expressed unwillingness to share contacts in case of positivity, according to their response to the questions “Please indicate your opinion about the following anti-epidemic measures”. The measures are named at each panel on the figure.

Agreement with anti-epidemic measures substantially improve the collaboration in contact tracing (Figs 6 and 7). Unwillingness was more prominent in respondents who disagreed that government might force people into self-isolation in case of contact with infected person (30.2% among persons strongly disagreed and 1.5% among strongly agreed with this action, p<0.0001) or disagreed with a need in increasing number of tests carried out in the population (36.0% and 2.3%, respectively, p<0.0001). Similarly, unwillingness to share contacts was more frequent in respondents who disagreed with compulsory face masks use in closed public spaces (26.2% among strongly disagreed and 2.0% among strongly agreed, p<0.0001), restrictions on visits to restaurants (15.8% and 1.8%, respectively, p<0.0001), distance learning in schools (15.8% and 2.5%, respectively, p<0.0001), introduction of curfew (18.3% and 1.8%, respectively, p<0.0001), ban on outside mass gatherings (35.8% and 2.1%, respectively, p<0.0001), mandatory testing of school teachers (33.2% and 2.0%, respectively, p<0.0001), closing between-countries borders countries (24.1% and 3.1%, respectively, p<0.0001). Only a small proportion of respondents expressed strong disagreement (2–4%), disagreement (2–4%) or mild disagreement (3–4%) with each of these measures, except the higher proportion of criticism to restrictions on visits to restaurants (10.8% indicated strong disagreement, 7.2% disagreement and 12.4% mild disagreement), distance learning in schools (8.9%, 6.8% and 11.1%, respectively) and curfew from 22:00 to 5:00 o’clock (9.2%, 6.7% 8.8%, respectively).

**Fig 8:**
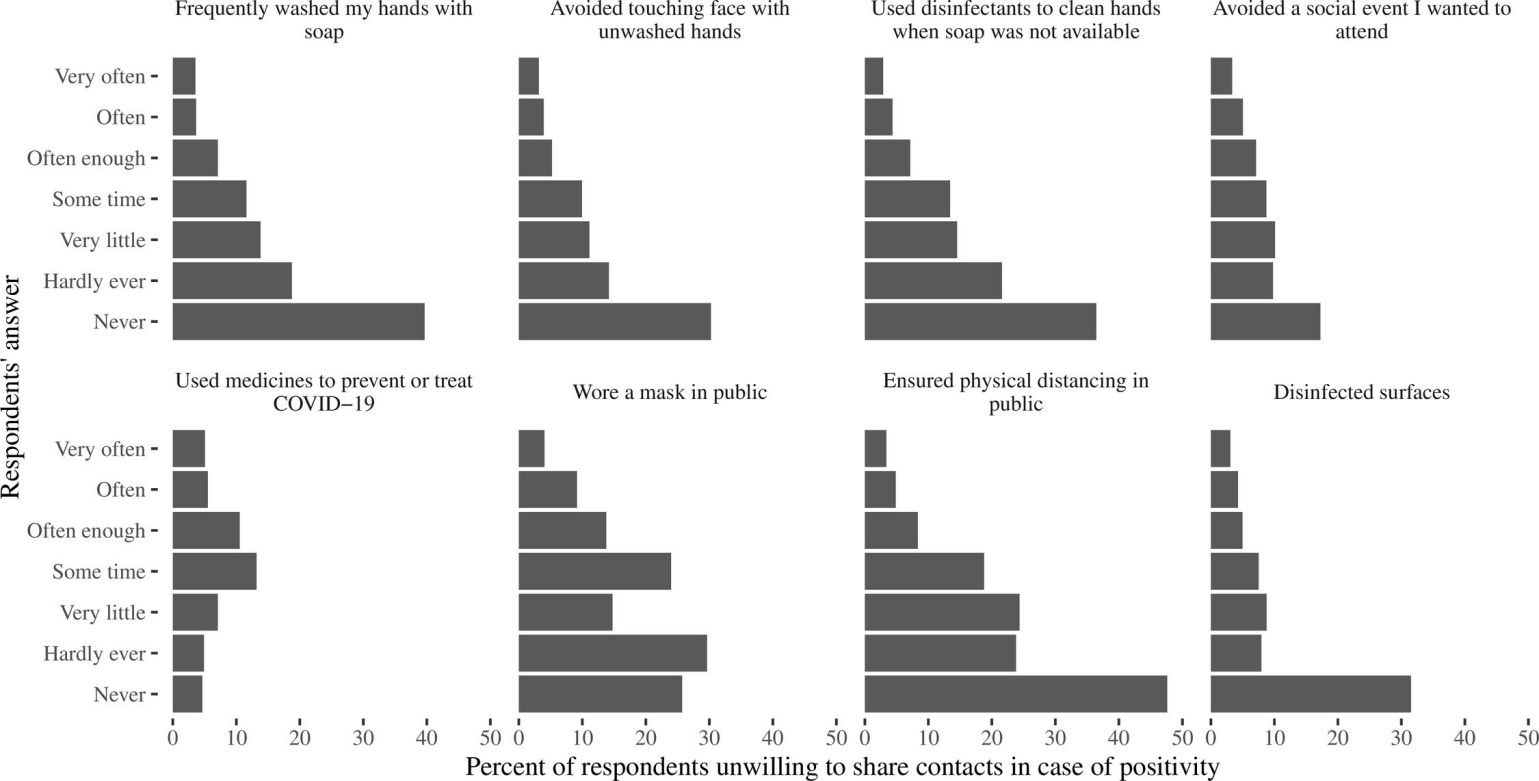
Percent of respondents who expressed unwillingness to share contacts in case of positivity, according to their response to the questions “During the past 7 days, which measures you have performed to prevent to be infected by COVID-19?”. The measures are named at each panel on the figure.

Personal adherence to preventive anti-epidemic measures (washing hands with soap, avoiding to touch face with unwashed hands, using disinfectants to clean hands, disinfecting surfaces) was related to lower prevalence of resistance to share contacts (3–4% compared to 30–40% responding “very often” and “never” following these recommendations, respectively) (Fig 8). Most importantly, those who were more resistant in sharing contacts had also statistically significant higher rates of not following collective rules to avoid attending social events (17.2% and 3.3% responded “never avoided” and “very often avoided” unwell to share contacts, respectively), wear a mask in public (25.7% and 4.0% responded “never” and “very often”, respectively), ensure physical distancing in public (47.6% and 3.4%, respectively).

#### Attitudes to testing and vaccination

A vast majority of respondents were willing to be tested in case of contact with an infected person and absence of symptoms in themselves. However, 10.8% supposed not to perform a test due to different reasons (see Supplementary section “Social behavior” in S1 File), and among them 26.5% were not willing to share contacts even if being tested positive (with higher rates of unwillingness as about 40–50% among those indicating economic or stigmatization fears), compared to 3.0% in those ready to make a test in this situation (p<0.0001).

Individuals who disagreed that all population should be vaccinated according to the national vaccination plan reported 19.6% rate of unwillingness to share contacts. The confidence that COVID-19 vaccines can help to control the epidemic spread substantially reduced the unwillingness to share contacts to 2.0% compared to 26.1% among people who strongly disagree with this (p<0.0001). The individuals who in theory do not want to become vaccinated if were already have had COVID-19 infection were also less prone to share contacts (19.5% and 2.3% of those who strongly agreed or disagreed, respectively, p<0.0001). Individuals who rely in their decision to vaccinate on family doctor or Ministry of Health expressed higher intention to contact sharing, with unwillingness in 1.5–2% among strongly agreed compared to 14–17% among strongly disagreed with this.

### Predictors of unwillingness to share contacts

To evaluate independent predictors of unwillingness, we performed univariable (S2 Table in S1 File) and then multivariable GLM analysis in the training data set representing 70% of respondents. From the 139 survey questions included in the forward selection multivariable analysis, the initial model (S3 Table in S1 File) selected 20 independent factors. This model had c-index 0.90 and McFadden R^2^ criteria 0.36, and demonstrated in the testing data set 93.2% (95% CI 92.1%-94.2%) accuracy, AUC 0.80, 31.2% sensitivity and 97.3% specificity. To reduce the number of factors, we excluded those having lower impact on the total model performance in terms of model residual deviance and AIC (See “Methods”, S2 Fig in S1 File), giving an exclusion priority to the parameters representing the same studied domain (trust, social behavior, etc). This allowed us to form a model with 10 variables that kept similar performance both in the training data set (c-index 0.87 and McFadden R^2^ criteria 0.29) and in the testing data set (93.1% (95% CI 92.0%-94.1%) accuracy, AUC 0.77, sensitivity 28.3% and specificity 97.3%). The advantage of this model was the inclusion of one factor from the aforementioned above domains (trust, etc). Additional efforts to simplify the regression model revealed four other parameters that had a relatively modest impact on the model, even if their removal led to some deterioration of the residual deviance by 17.2% and the AIC by 5.8% compared to the initial model. However, this 6-variables model kept similar performance in the training data set (c-index 0.85 and McFadden R^2^ criteria 0.25) and slightly better performance in the testing data set (93.3% (95% CI 92.1%-94.3%) accuracy, AUC 0.78, sensitivity 30.4% and specificity 97.4%). Considering these results, we have chosen the 6-variables model (Table 2) and further explored whether adding any interaction might improve the model performance. During this exploration we found statistically significant interactions between responses indicating not being aware about acquaintances infected with COVID-19 and patterns of surfaces disinfection, as well as responses indicating disagreement with the view that everyone should be vaccinated according to the national vaccination schedule and whether government should be allowed to force people into self-isolation (see Supplement section “Predictors of unwillingness to share contacts” in S1 File for details). However, we prefer not to include these interactions in the final model because they had not being reproduced in the adjacent categories that complicate their interpretation.

**Table 2:** Results of the Generalized Linear Model analysis showing variables associated with unwillingness to share contacts (see Supplementary for 20- and 10-variables models in S1 File).

	Generalized Linear Model analysis	
	Univariable	6-variables model
	OR (95%CI)	OR (95%CI)
**If you have been in contact with someone who tested positive for COVID-19 and have no symptoms yourself–will you get tested if you have the opportunity?**		
I would take the test for sure	Reference	Reference
I probably wouldn’t take the test	*13*.*03 (10*.*08–16*.*86*, *p<0*.*001)*	*5*.*60 (4*.*14–7*.*58*, *p<0*.*001)*
**The government should be allowed to force people into self-isolation if they have been in contact with someone who was infected**		
Strongly disagree	*4*.*96 (3*.*41–7*.*20*, *p<0*.*001)*	*1*.*79 (1*.*12–2*.*84*, *p = 0*.*014)*
Disagree	1.56 (0.86–2.68, p = 0.123)	0.94 (0.49–1.73, p = 0.859)
Slightly disagree	*1*.*67 (1*.*07–2*.*55*, *p = 0*.*019)*	1.60 (0.99–2.55, p = 0.051)
Neither disagree nor agree	Reference	Reference
Slightly agree	*0*.*40 (0*.*28–0*.*57*, *p<0*.*001)*	*0*.*59 (0*.*40–0*.*86*, *p = 0*.*007)*
Agree	*0*.*23 (0*.*14–0*.*38*, *p<0*.*001)*	*0*.*37 (0*.*21–0*.*62*, *p<0*.*001)*
Strongly agree	*0*.*18 (0*.*10–0*.*30*, *p<0*.*001)*	*0*.*33 (0*.*18–0*.*57*, *p<0*.*001)*
**Apart from COVID-19, I think everyone should be vaccinated according to the national vaccination schedule**		
Yes	Reference	Reference
No	*8*.*10 (6*.*12–10*.*72*, *p<0*.*001)*	*2*.*63 (1*.*86–3*.*69*, *p<0*.*001)*
Do not know	*2*.*57 (1*.*83–3*.*56*, *p<0*.*001)*	1.39 (0.96–1.99, p = 0.075)
**Which measures you performed to prevent the COVID-19 infection in the last 7 days: Disinfected surfaces**		
Never	*7*.*05 (4*.*51–11*.*02*, *p<0*.*001)*	*3*.*23 (1*.*85–5*.*59*, *p<0*.*001)*
Hardly ever	1.49 (0.76–2.73, p = 0.219)	0.81 (0.38–1.63, p = 0.576)
Very little	1.20 (0.67–2.05, p = 0.515)	0.91 (0.48–1.66, p = 0.767)
Some time	Reference	Reference
Often enough	*0*.*63 (0*.*42–0*.*94*, *p = 0*.*025)*	0.70 (0.45–1.08, p = 0.108)
Often	*0*.*66 (0*.*45–0*.*98*, *p = 0*.*040)*	0.93 (0.61–1.42, p = 0.728)
Very often	*0*.*40 (0*.*26–0*.*59*, *p<0*.*001)*	*0*.*56 (0*.*36–0*.*86*, *p = 0*.*009)*
**Ate more unhealthy food than I did before the pandemic**		
No	Reference	Reference
Yes	1.03 (0.77–1.36, p = 0.852)	1.28 (0.93–1.76, p = 0.127)
Not applicable	*2*.*55 (1*.*76–3*.*61*, *p<0*.*001)*	*2*.*63 (1*.*73–3*.*94*, *p<0*.*001)*
**Do you know people in your immediate social environment who are or have been infected with COVID-19 (suspected or confirmed)?**		
Yes	Reference	Reference
No	*2*.*14 (1*.*67–2*.*74*, *p<0*.*001)*	*1*.*66 (1*.*24–2*.*21*, *p = 0*.*001)*

In the best performing 6-variable model (Table 2) the most influential parameter was the intention to perform the test in the absence of symptoms if a person has been in contact with someone who tested positive for COVID-19. The lack of this intention increased the OR for unwillingness to share contacts by 13.03 (95% CI 10.08–16.86, p<0.001) in univariable analysis and by 5.60 (95% CI 4.14–7.58, p<0.001) in a fully adjusted multivariable analysis. The next most influential parameter was the judgment on whether the government should be allowed to force people into self-isolation if they have been in contact with someone who was infected, with an OR increased by 1.79 (95% CI 1.12–2.84, p = 0.014) among those who strongly disagree with this and significantly decreased by 0.33 (95% CI 0.18–0.57, p<0.001) among individuals who were strongly agree with this. Substantial role in unwillingness to share contacts played a position on whether everyone should be vaccinated according to the national vaccination schedule (apart from COVID-19), and people who disagree with this had 2.63 higher OR (95% CI 1.86–3.69, p<0.001) compared to those adherent to the vaccination. Lack of following to the recommendations of anti-COVID measures also increase the unwillingness rate, and in multivariable analysis persons who never disinfected surfaces in the last week had 3.23 (95% CI 1.85–5.59, p<0.001) higher OR, while those very often compliant with this practice had OR reduced by 0.56 (95% CI 0.36–0.86, p = 0.009). Of note, individuals who were just a little bit less extreme in the adherence to this practice (i.e. indicating “hardly ever” instead of “never”, or “often” instead “very often”) had similar OR for unwillingness to share contacts. Surprisingly prominent OR increase by 2.63 (95% CI 1.73–3.94, p<0.001) in unwillingness to share contacts, and a strong influence with persistence in all multivariable models demonstrated a subgroup of people who indicated that changing in eating habits were non applicable for them, in contrast to similar rates among those who agree or disagree that they ate more unhealthy food than before the pandemic. Finally, the absence of people in the immediate social environment who are or have been infected with COVID-19 (suspected or confirmed) increased OR for unwillingness to share contacts by 1.66 (95% CI 1.24–2.21, p = 0.001) compared to people who personally know someone already infected. Apart from these factors, the 10-variable model (S3 Table in S1 File) also included responses about the frequency of use of the trusted sources for COVID-related information, how easy or hard it was to understand the recommendation from authorities, perception of a speed of epidemic spread, and patterns of recovering from stress, and 20-variable model contained more than one factor from each of these domains.

A sensitivity analysis with application of PC method has shown a good representation of the original variance (S4 Table in S1 File). All PCs, except one, demonstrated mainly strong correlation with the willingness to share contact names (p<0.0001) (S3 Fig and S5 Table in S1 File). The final model comprises 9 statistically significant different variables (six of them were PCs, three were single questions) (S6 Table in S1 File). The PC analysis confirmed the importance of variables revealed in the GLM models, indicating significance of trust in authorities and in information provided by them, frequency in obtaining the COVID-19 related information and its understanding, adherence to anti-epidemic measures (including willingness to perform test), attitudes to vaccination, personal psychological patters, personal perception of the infection dangerousness, and a tendency to adhere to conspiracy theories.

## Discussion

Our analysis reveals that 5.5% of individuals would not like to share the names of contact persons in case of COVID-19 positivity. In a survey conducted in China in the early stages of the pandemic, 7.3% were not willing to report travel history to high-risk epidemic regions [12]. Other countries reported much higher rates of unwillingness to share contacts, reaching 33.7% of respondents in Nigeria [13]. Studies that investigated the willingness to use contact-tracing applications for smartphones have some similarity to the direct sharing of contact names, but they could not be compared due to more complex concerns about privacy and future use of data collected during the app-based tracing [14], a variety of information they collect [15] and lack of smartphones in a substantial proportion of population (especially high-risk older persons) even in industrialized countries [16,17].

Data on the studied topic are extremely limited in the literature, and our study provides important information about this aspect of COVID-19 related behaviour in a broader framework of anti-epidemic measures. In modelling studies, contact tracing demonstrated high effectiveness even in case of only partial non-household tracing [18], but universal collaboration and sharing contacts would provide better results. This implies important consequences to public health as one of the epidemic driving forces, and requires the development and implementation of adequate strategies to improve the willingness to collaborate for the public good.

Our analysis shows that unwillingness to share contacts is related to many reasons, and there is no single psychological or social profile that could explain this intention. However, as discussed below, it is possible to separate several major factors related to unwillingness to share contacts and address them on the population level. The major reasons indicated directly by unwilling respondents were the fear to disturb personal relationships with other persons in more than 50%, distrust in authorities in almost 30%, fear of economic losses in 25% and fear of stigmatization in 10%. Additional analyses of the factors related to unwillingness to share contacts shown a somewhat different and more complex picture.

### Trust as a key factor to promote people’s collaboration

The most prominent, almost ten-fold difference (2% vs 20%) in the percentage of those who would or would not share contacts was related to trust in health institutions (independently whether trust had to do with family physicians, Ministry of Health or National Institute of Health) and related to it willingness to follow the anti-epidemic recommendations. The multivariable analysis confirmed that the ability of government to obligate people to self-isolation, as one of the most evident manifestations of trust to authorities and one of the prominent interactions with a personal freedom, was among the core factors determining willingness to share contacts. Trust has been identified as a key factor for all aspects of COVID-19 epidemic [19,20] and in some settings even a pre-existing trust in institutions performing the tests was able to substantially increase the willingness to be tested [21]. Recent large-scale analysis revealed significantly lower COVID-19 infection rates in countries with higher measures of trust in government and interpersonal trust, and less government corruption [22]. Moreover, modeling study has shown that the global infections number would be reduced by more than 12% or 40% should the government trust and interpersonal trust, respectively, be improved to the level observed in countries with best trust indicators [22]. Different populations exhibit diverse patterns of trust, with higher rates of trust in health providers and central government than in local government [20] or higher trust in local authorities [23]. The latter seems to be especially important for contact tracing, and the UK experience indicates that local health teams trace eight times more contacts than national service [23]. At the beginning of the epidemic in Italy, the contact tracing at the local level has helped to identify almost twice more suspected COVID-19 cases compared to contact tracing managed by central regional authorities [24].

### Patterns of information perception

A proactive behaviour in search for COVID-19 related information substantially increases the rate of willingness to share contacts. Of note, most respondents were rather critical to the information received from social media or from famous persons that may suggest robust resilience to possible misinformation channeled through these sources. This indicates a leading role of expert recommendations from the public health authorities, as well as confidence of respondents in the ability to manage the epidemic by the authorities. However, almost 6% of respondents never, 8% almost never and 11% very seldom searched for this information. This population group not actively looking for the actual information requires other forms of communications, including gamification approaches for the information delivery with the use of computer games or quizzes [25–27] that proved to ameliorate the COVID-19 control practices [28]. The possibility to actively express personal thoughts and judgments about different aspects of the pandemic or frequency of discussions about them within the close social environment has not been analyzed in the survey, but might also be related to willingness to collaborate in case of infection, and appropriate strategies to facilitate such communication would increase contacts sharing.

In advance to this, the attention given to information about COVID-19 was one of the most important parameters associated with the willingness to share contacts (with a difference of about 2% vs 10%, or 5% vs 10%, depending on the question). Importantly, 10–40% of persons had difficulties in understanding the information related to the epidemic, and this percentage was not dependent on the educational attainment level. The highest influence on the decision to share contacts was represented by the difficulty in understanding restrictions and recommendations made by authorities in the novel scenario of pandemic, with an almost nine-fold difference (18% vs 2% in having or not having difficulties) in the proportion of individuals unwilling to share contacts. Difficulties in understanding government rules also was among major factors of decreased willingness to use contact-tracing app in the UK [29]. Interestingly, while some difficulties in understanding the recommendations was reported by 23.8% of respondents, the difficulty in following these recommendations was reported only by only 10.0% that may indicate the resistance to accept the sudden changes in the daily pre-epidemic lifestyle. The highest unwillingness to contact sharing was reported by subgroups that consist of people who do not understand or do not want to restrict social activities (about 27% compared to 3% in those who are ready to follow recommendations about social life). About 20% of individuals who disagreed to comply with preventive measures (e.g., mask use, physical distance, avoiding mass gathering) indicated they would not share contacts in case of known infection. Almost 10% of respondents would not perform the test even in case of contact with an infected person, and almost a quarter of them were resistant to share contacts. Moreover, the lack of intention to test for the COVID-19 has been revealed as the strongest factor of unwillingness to share contacts leading to almost 6-fold increase in odds ratio in the multivariable analysis. Thus, there was a fraction of respondents who were not willing to follow any preventive measures, did not want to test for a possible infection and would not communicate contacts even in case of positivity—that could be characterized as a “super-spreader” profile.

### Personal experience related to the COVID-19

Risk perception of contagion and awareness of the disease severity, and a more general understanding of the pandemic dynamics, were associated with a higher willingness to share contacts. Literature data indicates that worry and perceived threat substantially influence self-protective behaviors [30], and our data confirm these factors also extend to caring about others. Importantly, not only a personal experience, but also the awareness of close friends or relatives who were or had been infected with COVID-19 (both in suspected or confirmed forms), had substantially reduced the unwillingness rates. Also, a substantial part of those who were resistant to contact sharing based their choice by supposing this could cause inconvenience to contacts or because they wished to communicate directly with a contact person. These findings may indicate that uncertainty as how the infection positive would be perceived needs to be addressed by more education and experience sharing from people who already had the infection, and this approach would be extremely important especially at the earlier stages of an epidemic when the majority of people receive only formal instructions from the authorities but do not have an immediate experience with the disease. Of note, decision making by a person substantially depends on the acceptance of it in a social environment and might be considered as a part of social cohesion [31], that is also valid in the current pandemic [32]. For example, individuals who not yet willing to be vaccinated demonstrate social cohesion by indicating they would receive the vaccine when at average 64% of the general public become vaccinated, and this threshold lowered to 54% in case these individuals referred to people who they personally know [33]. From this perspective, it could be expected that the explicit knowledge about the acceptability of contact sharing in the social environment of individuals who currently express unwillingness to collaborate with the contact tracing teams would decrease the hesitancy.

Unwillingness to share contacts was high and reached almost 50% in relatively small subgroups of respondents who demonstrated overt fear of economic losses in case of quarantine or fear of stigmatization by others. A large Spanish survey has found that 28% of respondents lacked the necessary resources to properly isolate themselves [34]. In advance to this, individuals experiencing financial strain reported substantially diminished ability to test for a possible infection [35] or reduce mobility [34]. This probably indicates a need to extend some financial support programmes to the poorest strata of the population to improve adherence to diagnostic testing and isolation in the chain of COVID-19 transmission.

In our study the overt fear of stigmatization was expressed by almost 20% of those who would not share contacts (and probably this fear was masked by a part of respondents indicating they do not want to disturb contact persons), but they represented only a small part of all participants, while stigmatization fear could be expressed by 65–75% of individuals in countries of Africa [34] or Asia [36], by 57% in China [12] and by 46% (but only 7% expressed high stigma score) in the USA [37]. Reduced contacts sharing also occurs in other infections but mainly limited to highly stigmatisized sexually transmitted diseases [38,39].

### Personality traits

Our analysis also revealed several unexpected subgroups with higher rates of unwillingness to share contacts. In particular, people expressing extreme optimistic views about their economic condition, those who always feel cheerful and in good spirit, always feeling calm and relaxed, active and vigorous had higher proportion of those unwilling to share contacts. These findings are in line with the observation that persons with a self-reported "very good" health status make much lower use of a contact-tracing app [40], although another study [29] demonstrated those who felt their mood, anxiety or fear had improved were more willing to participate in contact tracing. Some other persons not willing to share contacts seem to had a predefined opinion that, as they reported, will not be influenced by any new information about different aspects of the epidemic, including the availability of information about vaccine efficiency. The nature of this rigidity should be evaluated in further studies, and could be only partially explained by adherence to conspiracy theories or vaccine hesitancy, even if the latter was among the factors leading to almost 2.5-fold higher rates of unwillingness to share contacts even in the multivariable analysis. Our findings could reflect effect of strategic ignorance that represents a universal phenomena for both communicable and non-communicable diseases [41,42] and is responsible for situation when people ignore information about risk or negative consequences of their behavior despite on availability of adequate information.

### Limitations

The study has several limitations. First, it does not cover some important aspects and has no information about income level, mobility requirement, profession, etc. These questions would hamper the response rate, especially keeping in mind the extended nature of the survey containing more than 150 questions. Second, any survey that concerns social expectations could suffer from a provision of socially acceptable answers, and the responses on unwillingness to share contacts in our analysis could be under-reported due to the social desirability of collaborative behaviour with contact sharing. This problem was minimised by the application of on-line anonymous survey. Moreover, different gender and employment status groups, and even health professionals who could feel different social pressure on expressing socially undesirable choices, reported similar unwillingness rates that most probably indicate the absence of substantial bias in responding about unwillingness of contact sharing. Third, the sampling technique was oriented to equally represent the national population and the survey has been performed using an internet-based platform that could lead to under-representation of some vulnerable groups like homeless people, persons with disabilities or migrants, each of them having their needs and requiring optimization of information provision and prevention of the disease. More studies in these important groups should be conducted to understand the patterns of contact sharing. Fourth, the multivariable model that selected independent predictors of unwillingness to share contacts had high specificity but modest sensitivity that indicates the model better classifies those who will share than those who will not, and suggests a need for further studies that would allow to reveal more factors that could better explain the unwillingness. However, the major goal of our analysis was to reveal the modifiable risk factors of unwillingness to collaborate with contact tracing teams that could be addressed by specific social interventions and lead to better control of the epidemic.

## Conclusions

In conclusion, our analysis indicated several groups in the general population that expressed unwillingness to collaborate on contact tracing and rather frequently had other features of a “super-spreader” pattern such as reluctance to mask wearing or not following rules for physical gathering. Distrust in health authorities, reluctance to perform the test, difficulties in finding or understanding the information about the infection or related recommendations, vaccine hesitancy, uncertainty on how the positivity would be perceived by others due to lack of personal experience or knowledge about other’s non-formal experience, fear of economic losses in case of quarantine were the major factors that need to be addressed in the anti-epidemic programmes. We suppose that the indicated patterns play a principal role not only in the COVID-19 epidemic but also important for possible future public health threats, and appropriate interventions for their correction should be developed and ready for the implementation.

## Supporting information

S1 File Additional information on the methods, extended description of several survey domains, S1-S3 Figs, S1-S6 Tables. (DOCX)
