# Supplementary material for: Willingness to share contacts in case of COVID-19 positivity–predictors of collaboration resistance in a nation-wide Italian survey
Source: PLoS One. 2022 Sep 27;17(9):e0274902. doi: 10.1371/journal.pone.0274902 (PMC9514658; doi:10.1371/journal.pone.0274902)
Supplement: S1 File — (DOCX) [file pone.0274902.s001.docx]

Supplementary information to the article

“Willingness to share contacts in case of COVID-19 positivity – predictors of collaboration resistance in a nation-wide Italian survey”

Content

[Supplementary information to the methods - 2 -](#_Toc108794391)

[S1 Fig. Performance of different discrimination thresholds for classification of testing data set. - 2 -](#_Toc108794392)

[Sensitivity analyses conducted using principal component (PC) analysis - 2 -](#_Toc108794393)

[Extended results description - 3 -](#_Toc108794394)

[Factors associated with unwillingness to share contacts - 3 -](#_Toc108794395)

[Demographic and social factors - 3 -](#_Toc108794396)

[Personal experience with COVID-19 infection - 4 -](#_Toc108794397)

[Patterns of information perception - 6 -](#_Toc108794398)

[Trust in information and authorities - 7 -](#_Toc108794399)

[Agreement with anti-epidemic measures - 8 -](#_Toc108794400)

[Relationship with vaccination - 9 -](#_Toc108794401)

[Social behavior - 10 -](#_Toc108794402)

[Predictors of unwillingness to share contacts - 11 -](#_Toc108794403)

[S2 Fig. Change in the Akaike information criterion of multivariable model during the elimination of separate factors. - 13 -](#_Toc108794404)

[S1 Table. Description of the survey participants (n=7513) according to the attitude for willingness to share contacts in case of positivity. - 13 -](#_Toc108794405)

[S2 Table. Results of univariable generalized linear model analysis with forward selection in the training data set (n=5259). - 32 -](#_Toc108794406)

[S3 Table. Extended results of multivariable analysis in the training data set. - 50 -](#_Toc108794407)

[S3 Fig. Results of Principal Components analysis. - 58 -](#_Toc108794408)

[S4 Table. Principal component proportion of explained variance and their association with unwillingness toward sharing names - 58 -](#_Toc108794409)

[S5 Table. Multivariate logistic model on association between principal components, summation indexes and single questions and unwillingness toward sharing names - 60 -](#_Toc108794410)

[S6 Table. Multivariate logistic model on association between principal components, summation indexes and single questions significantly related to unwillingness toward sharing names - 61 -](#_Toc108794411)

# Supplementary information to the methods

### S1 Fig. Performance of different discrimination thresholds for classification of testing data set.

Discrimination thresholds from 0.1 to 0.5 were evaluated for classification of testing data set for predicting probability of reporting the parameter of interest (unwillingness to share contacts).

### Sensitivity analyses conducted using principal component (PC) analysis

We conducted a sensitivity analysis following a completely different method, i.e. retaining the original subdivision of the questions into specific domains, in order to test again the conclusions of the primary analyses. We extracted the common underlying characteristics of the questions inside the specific domains performing a principal component (PC) analysis on each domain after transforming the ordinal responses into continuous ones, giving a value of one to the lowest category (for example, “strongly/completely disagree”) and a value of 7 to the highest category (for example, “strongly agree”). We set for a minimum proportion of explained variance of 60%, retaining only the first two PCs if the minimum was reached, or using additional PCs if the minimum was not reached. "Probability and severity" and "Preparedness and Perceived self-efficacy" were analysed within a single PC analysis due to their similarity and low number of questions (three and two respectively). In one domain with three questions ("Resilience") correlations among the variables were very poor, so the single questions were tested against willingness to share names. We employed a different strategy in two specific domains “Unwanted behaviour” and “Prevention-own behaviour”, where we computed a total from the answers to the single behaviours reported by the subjects, since the chosen questions lended themselves to use a single summation score. In four cases the domains had atypical structures: one domain ("Frequency of information seeking") had only an item that was inserted separately, two domains ("COVID-19 personal experience" and "Fairness") had only two questions, that did not have a high correlation between them, so both were inserted and one domain had a question ("National vaccination schedule should be obeyed") that was separated from the others, so it was inserted separately. Finally the "Testing and tracing" domain was not included since it contains the question on willingness/unwillingness to share names. PC extracted were unrotated orthogonal PCs. After obtaining the PCs we checked if their values were different between subjects willing/unwilling to share names using separate multivariate logistic regressions with correction for age, sex and financial situation: PCs which were significantly associated with willingness were moved on to a multivariate logistic regression analysis containing all PCs and the other summation and single questions. This model was then refined excluding PCs that did not attained a p<0.05 statistical significance. Age and sex were retained as forced variables.

# Extended results description

## Factors associated with unwillingness to share contacts

### Demographic and social factors

Sex, education level, and employment status have not influenced on the decision to share contacts (Supplementary table S1). Even among health professionals, the percentage did not dramatically differ from the general population (3.8% and 5.5%, p=0.17).

Age only marginally influenced the decision not to share contacts (6.7%, 5.8%, 5.5% and 4.4% in the age groups 15-34, 35-44, 45-54 and 55-70 years, respectively, p<0.02). Citizens of settlements with less than 100,000 inhabitants demonstrated lower frequency of unwillingness to share contacts compared to more populated areas (5.3% vs 6.5%, p=0.057). Household composition was associated with the study parameter, with higher proportion among those who live alone (6.3%) and those who live with other adults including vulnerable (older than 65 years or having chronic conditions) persons (6.6%), and lower proportion among people living in a household including both children, adults and vulnerable persons (2.2%), even if this difference did not reach statistical significance (p=0.34), probably due to a small number of respondents in these groups. The majority of households were composed of several adult persons or adults with children [but without vulnerable persons] (50.2% and 18.8%, respectively), and respondents from these households reported 5.8% and 5.1% of unwillingness, respectively.

Interestingly, 8.2% of persons who responded that they did not know whether they had chronic illness would not like to share contacts, compared to 4.5% and 5.7% among people reporting presence or absence of any chronic illness, respectively (p<0.04).

### Personal experience with COVID-19 infection

As much as 4.5% of respondents who have already had COVID-19 infection indicated they would not share contacts, compared to 5.2% among those who think never been infected, and to 10.1% who responded they do not know whether they have been infected or not (p<0.0001). However, even the subgroup of 637 persons with previous COVID-19 infection demonstrated a substantial heterogeneity in unwillingness to share contacts, reported by 3.7% persons with the disease confirmed by test and 10.5% with no test confirmation (p<0.01), and not statistically significant difference between 4.4% of having mild and 6.5% severe clinical symptoms (p=0.46). Among the total sample, even the presence of people in the immediate social environment who are or have been infected with COVID-19 (both in suspected or confirmed forms) has substantially reduced the proportion of those not willing to share contacts to 4.4% compared to 8.6% among individuals who did not know about COVID-19 cases in the nearest social environment (p<0.0001). Those who had known ill persons in their social environment expressed unwillingness to share contacts in 12.2% if the disease of others was not confirmed by the test compared to only 4.3% in case of test confirmation (p<0.002), while these percentages were 3.5% and 5.7% if they knew or did not know, respectively, someone who died from COVID-19 (p<0.0001).

Willingness to share contact names heavily relied on personal beliefs about a probability of getting COVID-19 (4.2% and 11.1% [p<0.0001] supposing “very probable” and “very improbable”, respectively, would not will to share contacts) and susceptibility to the infection (3.7% and 14.0% [p<0.0001] supposing “very susceptible” and “very insusceptible”, respectively), as well as about a probable severity of the infection (3.9% and 17.2% [p<0.0001] supposing it could be “very severe” and “not at all severe”, respectively). General perception of pandemic situation also played a role, and resistance to share was reported only by 2.5% of persons feeling COVID-19 is “close to her/him” compared to 12.2% feeling it is “far from her/him” (p<0.0001), only by 2.3% of acknowledging that pandemic is rising high compared to 9.9% supposing it rising low (p<0.0001). Respondents who reported to think about pandemic were more prone to share contacts compared to those who responded ”never think about pandemic” (3.0% vs 16.1%, p<0.0001), as well as those who think that the pandemic is scaring (2.1% indicating it is scaring vs 21.6 indicating it is not scaring, p<0.0001), stressing (4.9% indicating it is stressing vs 12.7 indicating it is not, p<0.0001) or feel it makes a person vulnerable (3.1% indicating they feel lack of control vs 10.3% feeling they could manage it, p<0.0001). The personal perception that the pandemic has been overstated/dramatized by media was correlated with unwillingness to share contacts (with gradual decrease from 11.7% in agreeing to 1.6% in not agreeing it was overstated, p<0.0001). Surprisingly, the responses to questions about the knowledge on how to protect themselves from coronavirus (5.2% had difficulties in protection knowledge) and easiness in avoiding the infection (13.1% reported difficulties) demonstrated non-uniform heterogeneity between different responses — that is hard to interpret.

Persons having responded they are strongly agree or disagree to had a fast recovery from stress (p<0.0001) and those who easily recovered from stress (p<0.0005) during the pandemic were tended to have higher unwillingness rates compared to those who expressed less contrast opinions, while general susceptibility to stressful events had not influenced this decision (p=0.14).

Unexpectedly, a small proportion of 2.5% of respondents who strongly disagreed of being worried about future economic consequences related to the pandemic (and thus having more optimistic view on personal economic perspectives) were less likely to share contacts (13.3% vs 4-6% in groups expressing other opinions, p<0.0001). Also, a small subgroup of 3.5% of participants who reported to feel cheerful and in good spirits all the time and a subgroup of 7.0% of participants who reported never feel cheerful had demonstrated higher unwillingness to share contacts as 10.2% and 7.0%, respectively, compared to other respondents (p<0.0001). Similarly, in a small subgroup of about 4% of respondents, unwillingness was much higher and reached 10.0% among those who felt calm and relaxed all the time, 10.5% in always felt active and vigorous, 8.9% among always woke up feeling fresh and rested, as 8.9% in always felt the life filled with interesting things during the last 2 weeks.

### Patterns of information perception

Difficulties in finding the information related to COVID-19 has been reported by 11.6% of respondents, and this increased the unwillingness to share contacts to 7.4% compared to 3.2% among people who find this information easily (p>0.0001). These difficulties in finding the information were faced by 13.2% of people with beginning/middle school education, by 9.9% having terminated higher school, and by 11.4% having graduate education. Difficulties in understanding information about what to do in case of having COVID-19 also increased the unwillingness to 7.0% compared to 4.7% in those who easily understand it (p<0.0002, reaching 10.2% of those who reported “very difficult”), and these difficulties were uniformly reported by about 19% of participants with any education level. Difficulties in judging whether the information about COVID-19 in the media is reliable were reported by 38.6% of individuals, but it has no direct correlation with the unwillingness to share contacts which was highest among respondents reported that the judgment was “very difficult” or “very simple” (8.1% and 7.6%, respectively). Understanding restrictions and recommendations of authorities regarding COVID-19 was difficult for 23.8% of respondents (and did not correlate with the education level), and unwillingness to share contacts steeply decreased from 18.2% among responding “very difficult” to 2.5% responding “very easy” (p<0.0001). Almost 10% had difficulties in following the recommendations to protect from COVID-19 (10.6%, 8.4% and 9.0% in groups having middle, higher and graduated education, respectively), and this substantially evaluate the proportion of persons not willing to share contacts up to 11.4%, compared with 4.2% among individuals for whom it was easy (p<0.0001). Similarly, 15.3% had difficulties in understanding recommendations about when to stay at home from work/school (16.4%, 14.0% and 15.3% for different education levels), that increased the unwillingness to 10.3% compared to 3.9% in reporting easy understanding (p<0.0001). Also, 14.5% faced difficulties in following recommendations about when to stay at home, and 29.0% of those who responded “very difficult” (representing about 2% of participants in any educational level) did not will to share contacts compared to 2.6% who responded “very easy” (p<0.0001). Very similar results in willingness were observed regarding responses about difficulties in understanding recommendations about when to not engage in social activities (26.1% and 2.6% [p<0.0001] of those who responded “very difficult” and “very easy”, respectively), and following these recommendations (26.8% and 2.7%, respectively, p<0.0001).

Persons utilizing more frequently mass media for obtaining COVID-19 related information were more prone to share contacts, with a little bit higher influence of television than newspapers or radio, and non-linear correlation with the use of COVID-19 hotlines, social media and information from famous persons. The highest influence had frequent information received from health workers (2.5% and 11.7% of persons receiving from them information “very frequently” and “never”, respectively, would not be willing to share contacts, p<0.0001), Ministry of Health (2.5% and 14.0%, respectively, p<0.0001), Center for Disease Control (2.8% and 14.0%, respectively, p<0.0001), WHO (3.0% and 12.4%, respectively, p<0.0001), national COVID-19 information website (2.1% and 10.3%, respectively, p<0.0001). In general, the frequency with which a person search information about COVID-19 positively correlated with willingness to share contacts, with about 3% unwilling to share in groups reported frequent search instead of 15.1% among those indicated never searched this information (p<0.0001). The proportion of respondents who never searched for the COVID-19 related information reached 6.1%, almost never – 8.1%, and very seldom – 11.4%.

### Trust in information and authorities

Trust in information substantially increases willingness to share contacts, whether this trust has concerned the information received from television (contacts would not be shared by 4.8% and 14.2% of persons trusting a lot and not trusting at all, respectively, p<0.0001), newspapers (5.7% and 12.9%, respectively, p<0.0001), radio (4.3% and 13.5%, respectively, p<0.0001), health workers (2.6% and 26.4%, respectively, p<0.0001), Ministry of Health (2.1% and 22.7%, respectively, p<0.0001), Center for Disease Control (1.9% and 22.6%, respectively, p<0.0001), World Health Organization (2.1% and 20.1%, respectively, p<0.0001), COVID-19 Hotlines (3.3% and 15.2%, respectively, p<0.0001), National COVID-19 information website (2.9% and 20.5%, respectively, p<0.0001), but this did not concern social media (5.1% and 6.7%, respectively, p=0.22) and famous persons (unwillingness was J-shaped with more frequent percentage as 7.1-7.7% in both very trusting and very distrusting to them, p<0.01).

The confidence of respondents in ability to manage the epidemic by any health authorities (including family doctor, hospitals, Ministry of Health, Center for disease Control) and police positively and statistically significantly correlated with willingness to share contacts in case of a possible infection, and a percentage of people who would not like to share contacts was close to 2-3% among those who “trust a lot” compared to 15-20% in those who “do not have any trust”. Confidence in the ability of other non-medical public institutions to manage the epidemic had less prominent effect on willingness to collaborate with contact tracing teams, with major difference between 9-10% in those who responded they “do not trust at all” compared to 4-5% in groups with a milder form of distrust or trust in case of confidence to employers (p<0.005) and church (p<0.005), and tended to have a J-shape form with higher proportion of unwilling to share contacts in both subgroups who “trust a lot” and “do not trust at all” in case of confidence to schools (p<0.001) and public transportation companies (p=0.06).

Overt adherence to conspiracy theories was significantly related to unwillingness to share contacts, with about 8-12% of those completely agree with statements like "Politicians usually do not tell us the true motives for their decisions", "There are secret organisations that greatly influence political decisions", "Government agencies closely monitor all citizens", "Events which superficially seem to lack a connection are often the result of secret activities" (these statements were "completely agreed" by 19.9%, 10.3%, 7.8%, 6.8% of all respondents, respectively), compared to 3-6% of expressing milder agreement or disagreed with them.

### Agreement with anti-epidemic measures

Individuals were less likely to share contacts if they expressed disagreement to the measures of the Italian authorities for curbing the epidemic (18.5% who strongly disagreed and 1.5% who strongly agreed that the decisions were fair, p<0.0001) or percept the restrictions as being exaggerated (19.8% and 2.6%, respectively, p<0.0001). Unwillingness was even more prominent in respondents who disagreed that government might force people into self-isolation in case of contact with infected person (30.2% among persons strongly disagreed and 1.5% among strongly agreed to a possibility of a forced self-isolation, p<0.0001) or disagreed with a need in increasing number of tests carried out in the population (36.0% and 2.3%, respectively, p<0.0001). Similarly, unwillingness to share contacts was more frequent in respondents who disagreed with compulsory face masks use in closed public spaces (26.2% among strongly disagreed and 2.0% among strongly agreed, p<0.0001), restrictions on visits to restaurants (15.8% and 1.8%, respectively, p<0.0001), distance learning in schools (15.8% and 2.5%, respectively, p<0.0001), introduction of curfew (18.3% and 1.8%, respectively, p<0.0001), ban on outside mass gatherings (35.8% and 2.1%, respectively, p<0.0001), mandatory testing of school teachers (33.2% and 2.0%, respectively, p<0.0001), closing between-countries borders countries (24.1% and 3.1%, respectively, p<0.0001). Only a small proportion of respondents expressed strong disagreement (2-4%), disagreement (2-4%) or mild disagreement (3-4%) with each of these measures, except the higher proportion of criticism to restrictions on visits to restaurants (10.8% indicated strong disagreement, 7.2% disagreement and 12.4% mild disagreement), distance learning in schools (8.9%, 6.8% and 11.1%, respectively) and curfew from 22:00 to 5:00 o’clock (9.2%, 6.7% 8.8%, respectively).

### Relationship with vaccination

The confidence that vaccines can help to control the COVID-19 spread substantially reduced the unwillingness to share contacts from 26.1% among people who strongly disagree to 2.0% of those strongly agree with this (p<0.0001). At the moment of the survey conduction the vaccines were not yet widely available, and the following responses reflected more the expectations of persons than the experience or awareness about accumulated knowledge considering vaccine effectiveness. In a subsample of 8.6% individuals who refused the proposed vaccination, 15.2% unwilling to share contacts in case of a possible infection compared to 2.5% among those who accepted the vaccination proposal (p<0.0005). In a general survey, the individuals who in theory do not want to become vaccinated if were already have had COVID-19 infection were also less prone to share contacts (19.5% and 2.3% of those who strongly agreed or disagreed, respectively, p<0.0001).

Individuals who rely in their decision to vaccinate on family doctor or Ministry of Health expressed more intention to collaborate with contact sharing, with unwillingness in 1.5-2% among strongly agreed and in 14-17% among strongly disagreed (p<0.0001) that the decision will depend on these recommendations. Interestingly, that compared to other respondents, unwillingness to share contacts reached 8-10% in persons who indicated their decision to vaccinate will not depend in no way on long-term experience and information about lack of serious side-effects, experience of other countries, personal risk of the infection at the moment of vaccination, commodity to receive a vaccination, type of vaccine, and even not depend on whether the vaccine will remove restrictions on travel and gathering, while even among persons indicated slightly less categorical opinion on these questions unwillingness was close to the average 5% level. To better understand why these individuals, accounting to 6-12% of the respondents (depending on the question), did not want to consider particular arguments for the anti-Covid vaccine, we evaluate their relationships with a general anti-vaccination prejudice. In general, unwillingness to share contacts was indicated by 19.6% of individuals who disagreed that all population should be vaccinated according to the national vaccination plan. However, the persons who disagreed with the national vaccination plan represented only 10-20% of those indicating their decision on anti-Covid vaccination will not depend in no way on aforementioned factors.

### Social behavior

Individuals who followed the recommendations to frequently wash hands with soap, avoiding to touch face with unwashed hands, using disinfectants to clean hands, disinfected surfaces had lower prevalence of resistance to share contacts (3-4% compared to 30-40% responding “very often” and “never” following these recommendations, respectively). Most importantly, those who were more resistant in sharing contacts had also statistically significant higher rates of not following rules to avoid attending social events (17.2% and 3.3% responded “never avoided” and “very often avoided” unwell to share contacts, respectively), wear a mask in public (25.7% and 4.0% responded “never” and “very often”, respectively), ensure physical distancing in public (47.6% and 3.4%, respectively). However, a decision to stay at home from work/school had no particular pattern in willingness to share contacts (6.0% and 5.3% [p=0.32], respectively).

In case of contact with an infected person and absence of symptoms in themselves, a vast majority were willing to be tested, but 10.8% supposed not to perform a test in this case. In a subsample of respondents (n=6704) who indicated they would certainly make a test, the majority base this decision on understanding that by performing a test they could protect others (70.8%), feeling a responsibility as a citizen (67.9%), understanding this could stop the epidemic (60.8%), or wishing to receive appropriate treatment in case of the disease (48.6%) (the sum exceeds 100% because of multiple answers). However, even among individuals expressing the commitment to perform a test, about 2% unwilled to share their contacts in case of being positive. Fewer persons grounded the decision to perform a test on their family or friends expectations (23.4%) or willing to avoid sanctions (7.3%), and among them about 3% unwilled to share the contacts.

In a subsample of individuals (n=809) who indicated they would avoid to make a test, the majority explained this intention by several reasons: because they did not think that tests were reliable (32.4%), feeling it will not be possible to do anything in case of positivity (20.9%), because making a test would require expenditures (20.5%), fear of being infected in a testing unit (20.3%) or fear that test procedure could be painful (15.1%), lack of possibility of self-isolation in case of positivity (13.8%), fear of income loss either during the waiting for test results (14.5%) or if a quarantine would be needed in case positivity (12.6%). Less percentage, not exceeding 10% for each response, would not be willing to make a test because of fear of some penalty, stigmatization by others due to the positivity or due to behavior that led to positivity, reluctance to share their personal data with authorities, the lack of information about where to perform a test or fear it would require a lot of time. The least frequent reason was the conviction that COVID-19 does not exist (4.0% among 809 of those who would avoid testing). Not surprisingly, 26.4% of people expressing any of these reasons to avoid testing were also not willing to share contacts in case of positivity (with higher rates of unwillingness as about 40-50% among those indicating economic or stigmatization fears).

During the pandemic a faction of respondents have changed their habits and reported to exercise less, eat more unhealthy food, to smoke or drink more alcohol – but this does not seem to have a substantial correlation with the intention to share contacts. Notably, a relatively minor group of about 15% of respondents who indicated that changes in these habits were not applicable to them, demonstrated higher rates of unwillingness to share contacts (for example, the percentage of unwillingness was about 5% in those who agreed or disagreed they eat more unhealthy food than before the pandemic, while it achieved 11.5% in those who indicated this was not applicable to them, p<0.0001). Similarly higher rates of unwillingness to share contacts were observed among respondents who indicated “not applicable” for the question whether they avoided going to a doctor for non-COVID reasons.

## Predictors of unwillingness to share contacts

To evaluate independent predictors of unwillingness, we performed univariable (Supplementary Table S2) and then multivariable GLM analysis in the training data set representing 70% of respondents. We have chosen for the analysis the multivariable model with forward selection because it had better performance compared to the model with backward selection (difference in residual deviance -21.987, p<0.005). From the selected in univariable analysis 139 survey questions, the initial model (Supplementary Table S3) selected 20 independent factors. This model had c-index 0.90 and McFadden R^2^ criteria 0.36, and demonstrated in the test data set 93.2% (95%CI 92.1%-94.2%) accuracy, AUC 0.80, 31.2% sensitivity and 97.3% specificity.

To reduce the number of factors, we excluded those having lower impact on the total model performance in terms of model residual deviance and AIC (See “Methods”, Supplementary Figure S2), giving an exclusion priority to the parameters representing the same studied domain (trust, social behavior, etc). This allowed us to form a model with 10 variables (Supplementary Table S3) that had higher residual deviance and higher AIC compared to the initial model (difference in residual deviance 163.62, difference in AIC 65.624, p<0.0005), that represented 10.6% of the residual deviance and 4.2% of the AIC of the initial model. This 10-variables model kept similar performance both in the training data set (c-index 0.87 and McFadden R^2^ criteria 0.29) and in the test data set (93.1% (95%CI 92.0%-94.1%) accuracy, AUC 0.77, sensitivity 28.3% and specificity 97.3%). The advantage of this model was the inclusion of one factor from the aforementioned above domains (trust, etc). Additional efforts to simplify the regression model revealed other four parameters that had a relatively modest impact on the model. However, the additional removal of these four variables demonstrated more prominent drop in performance (difference in residual deviance 237.88, difference in AIC 91.881, p<0.0005), representing 17.2% of the residual deviance and 5.8% of the AIC of the initial model. This 6-variables model kept similar performance in the training data set (c-index 0.85 and McFadden R^2^ criteria 0.25) and slightly better performance in the test data set (93.3% (95%CI 92.1%-94.3%) accuracy, AUC 0.78, sensitivity 30.4% and specificity 97.4%). Considering these results, we have chosen the 6-variables model and further explored whether adding any interaction might improve the model performance. During this exploration we found significant interactions between responses indicating not being aware about acquaints infected with COVID-19 and (1) never disinfected surfaces [OR for interaction 0.3 (95% CI 0.1-0.95, p<0.05)], (2) often enough [OR for interaction 0.4 (95% CI 0.2-1, p=0.059)] or (3) often disinfected surfaces as an anti-COVID-19 measure [OR for interaction 0.2 (95% CI 0.1-0.5, p<0.005)], as well as responses indicating being disagree that everyone should be vaccinated according to the national vaccination schedule and (4) agree [OR for interaction 5.9 (95% CI 1.6-23.6, p<0.01)] or (5) do not know [OR for interaction 4.6 (95% CI 1.1-19.4, p<0.05)] whether government should be allowed to force people into self-isolation if they have been in contact with someone who was infected. Even if these interactions were statistically significant and improve the classification (compared to the model without interaction the difference in residual deviance 39.885, the difference in AIC 3.885, p<0.005) we prefer not to include these interactions in the final model because they had not being reproduced in the adjacent categories that complicate their interpretation.

The paramenters revealed in the best performing 6-variable model (without interaction terms) are described in the main manuscript (see “Predictors of unwillingness to share contacts” section).

### S2 Fig. Change in the Akaike information criterion of multivariable model during the elimination of separate factors.

Change in the Akaike information criterion (AIC) of multivariable model (produced on the training data set) related to elimination of distinct factors. Lower values of AIC suggest the model has better fit.

### S1 Table. Description of the survey participants (n=7513) according to the attitude for willingness to share contacts in case of positivity.

| **Survey parameter** | **Willingness to share contacts in case of positivity** | | **p** | **Percentage by responding category** | |
| --- | --- | --- | --- | --- | --- |
|  | Would  share | Would not share |  | Would share | Would not share |
| **Age, years** | 45.9 (12.8) | 43.5 (12.9) | <0.001 | - | - |
| **What is your sex?** | | | | | |
| Male | 3506 (94.1) | 218 (5.9) | 0.254 | 49.4% | 52.4% |
| Female | 3591 (94.8) | 198 (5.2) |  | 50.6% | 47.6% |
| **Age group** | | | | | |
| 18-34 years | 1814 (93.3) | 131 (6.7) | 0.011 | 25.6% | 31.5% |
| 35-44 years | 1365 (94.2) | 84 (5.8) |  | 19.2% | 20.2% |
| 45-54 years | 1685 (94.5) | 98 (5.5) |  | 23.7% | 23.6% |
| 55-70 years | 2233 (95.6) | 103 (4.4) |  | 31.5% | 24.8% |
| **What is the most advanced education have you completed?** | | | | | |
| elementary school / junior high school | 2905 (94.4) | 173 (5.6) | 0.354 | 40.9% | 41.6% |
| high school | 2369 (94.9) | 126 (5.1) |  | 33.4% | 30.3% |
| degree or more | 1823 (94.0) | 117 (6.0) |  | 25.7% | 28.1% |
| **Are you employed?** | | | | | |
| Yes | 3732 (94.7) | 208 (5.3) | 0.329 | 52.6% | 50.0% |
| No | 3365 (94.2) | 208 (5.8) |  | 47.4% | 50.0% |
| **Where do you live?** | | | | | |
| Rural zone (up to 100.000 inhabitants) | 5444 (94.7) | 302 (5.3) | 0.063 | 76.7% | 72.6% |
| Urban zone (more than 100.000 inhabitants) | 1653 (93.5) | 114 (6.5) |  | 23.3% | 27.4% |
| **Do you have a chronic illness?** | | | | | |
| Yes | 1636 (95.4) | 78 (4.6) | 0.027 | 23.1% | 18.8% |
| No | 5202 (94.3) | 315 (5.7) |  | 73.3% | 75.7% |
| Do not know | 259 (91.8) | 23 (8.2) |  | 3.6% | 5.5% |
| **Please assess your private financial situation over the past three months:** | | | | | |
| Improved | 379 (92.0) | 33 (8.0) | <0.001 | 5.3% | 7.9% |
| Stayed the same | 4231 (95.2) | 211 (4.8) |  | 59.6% | 50.7% |
| Worsened | 2424 (93.8) | 160 (6.2) |  | 34.2% | 38.5% |
| Do not know | 63 (84.0) | 12 (16.0) |  | 0.9% | 2.9% |
| ***COVID-19 personal experience*** | | | | | |
| **To your knowledge, are you, or have you been, infected with COVID-19?** | | | | | |
| Yes | 608 (95.4) | 29 (4.6) | <0.001 | 8.6% | 7.0% |
| No | 5917 (94.8) | 323 (5.2) |  | 83.4% | 77.6% |
| Do not know | 572 (89.9) | 64 (10.1) |  | 8.1% | 15.4% |
| **Do you know people in your immediate social environment who are or have been infected with COVID-19 (suspected or confirmed)?** | | | | | |
| Yes | 5260 (95.6) | 243 (4.4) | <0.001 | 74.1% | 58.4% |
| No | 1837 (91.4) | 173 (8.6) |  | 25.9% | 41.6% |
| ***COVID-19 literacy*** | | | | | |
| **How easy or hard it was to:** | | | | | |
| **…find the information you need related to COVID-19?** | | | | | |
| Very difficult | 179 (94.2) | 11 (5.8) | <0.001 | 2.5% | 2.6% |
| Hard | 152 (92.1) | 13 (7.9) |  | 2.1% | 3.1% |
| Pretty hard | 479 (92.1) | 41 (7.9) |  | 6.7% | 9.9% |
| Neither difficult nor easy | 1286 (92.1) | 110 (7.9) |  | 18.1% | 26.4% |
| Quite easy | 2382 (95.6) | 110 (4.4) |  | 33.6% | 26.4% |
| Easy | 1445 (94.8) | 79 (5.2) |  | 20.4% | 19.0% |
| Very easy | 1174 (95.8) | 52 (4.2) |  | 16.5% | 12.5% |
| **…understand information about what to do if you think you have COVID-19?** | | | | | |
| Very difficult | 123 (89.8) | 14 (10.2) | 0.001 | 1.7% | 3.4% |
| Hard | 318 (94.4) | 19 (5.6) |  | 4.5% | 4.6% |
| Pretty hard | 875 (93.0) | 66 (7.0) |  | 12.3% | 15.9% |
| Neither difficult nor easy | 1337 (93.1) | 99 (6.9) |  | 18.8% | 23.8% |
| Quite easy | 2339 (94.9) | 125 (5.1) |  | 33.0% | 30.0% |
| Easy | 1349 (96.2) | 54 (3.8) |  | 19.0% | 13.0% |
| Very easy | 756 (95.1) | 39 (4.9) |  | 10.7% | 9.4% |
| **…judge if the information about COVID-19 in the media is reliable?** | | | | | |
| Very difficult | 668 (91.9) | 59 (8.1) | 0.008 | 9.4% | 14.2% |
| Hard | 557 (93.9) | 36 (6.1) |  | 7.8% | 8.7% |
| Pretty hard | 1497 (94.7) | 84 (5.3) |  | 21.1% | 20.2% |
| Neither difficult nor easy | 1826 (94.6) | 104 (5.4) |  | 25.7% | 25.0% |
| Quite easy | 1531 (95.6) | 70 (4.4) |  | 21.6% | 16.8% |
| Easy | 691 (95.0) | 36 (5.0) |  | 9.7% | 8.7% |
| Very easy | 327 (92.4) | 27 (7.6) |  | 4.6% | 6.5% |
| **…understand restrictions and recommendations of authorities regarding COVID-19?** | | | | | |
| Very difficult | 252 (81.8) | 56 (18.2) | <0.001 | 3.6% | 13.5% |
| Hard | 445 (93.1) | 33 (6.9) |  | 6.3% | 7.9% |
| Pretty hard | 940 (94.1) | 59 (5.9) |  | 13.2% | 14.2% |
| Neither difficult nor easy | 1253 (92.5) | 101 (7.5) |  | 17.7% | 24.3% |
| Quite easy | 2014 (95.7) | 91 (4.3) |  | 28.4% | 21.9% |
| Easy | 1330 (96.1) | 54 (3.9) |  | 18.7% | 13.0% |
| Very easy | 863 (97.5) | 22 (2.5) |  | 12.2% | 5.3% |
| **…follow the recommendations on how to protect yourself from COVID-19?** | | | | | |
| Very difficult | 151 (83.0) | 31 (17.0) | <0.001 | 2.1% | 7.5% |
| Hard | 116 (81.7) | 26 (18.3) |  | 1.6% | 6.2% |
| Pretty hard | 364 (93.8) | 24 (6.2) |  | 5.1% | 5.8% |
| Neither difficult nor easy | 979 (91.2) | 94 (8.8) |  | 13.8% | 22.6% |
| Quite easy | 2312 (95.0) | 121 (5.0) |  | 32.6% | 29.1% |
| Easy | 1792 (96.0) | 74 (4.0) |  | 25.3% | 17.8% |
| Very easy | 1383 (96.8) | 46 (3.2) |  | 19.5% | 11.1% |
| **…understand recommendations about when to stay at home from work/school, and when not to?** | | | | | |
| Very difficult | 121 (72.0) | 47 (28.0) | <0.001 | 1.7% | 11.3% |
| Hard | 183 (89.3) | 22 (10.7) |  | 2.6% | 5.3% |
| Pretty hard | 729 (93.6) | 50 (6.4) |  | 10.3% | 12.0% |
| Neither difficult nor easy | 1218 (92.4) | 100 (7.6) |  | 17.2% | 24.0% |
| Quite easy | 2258 (95.8) | 98 (4.2) |  | 31.8% | 23.6% |
| Easy | 1624 (96.0) | 68 (4.0) |  | 22.9% | 16.3% |
| Very easy | 964 (96.9) | 31 (3.1) |  | 13.6% | 7.5% |
| **…follow recommendations about when to stay at home from work/school, and when not to?** | | | | | |
| Very difficult | 110 (71.0) | 45 (29.0) | <0.001 | 1.5% | 10.8% |
| Hard | 256 (90.1) | 28 (9.9) |  | 3.6% | 6.7% |
| Pretty hard | 596 (92.1) | 51 (7.9) |  | 8.4% | 12.3% |
| Neither difficult nor easy | 1160 (91.6) | 107 (8.4) |  | 16.3% | 25.7% |
| Quite easy | 2330 (95.9) | 100 (4.1) |  | 32.8% | 24.0% |
| Easy | 1619 (96.5) | 58 (3.5) |  | 22.8% | 13.9% |
| Very easy | 1026 (97.4) | 27 (2.6) |  | 14.5% | 6.5% |
| **…understand recommendations about when to engage in social activities, and when not to?** | | | | | |
| Very difficult | 116 (73.9) | 41 (26.1) | <0.001 | 1.6% | 9.9% |
| Hard | 172 (87.3) | 25 (12.7) |  | 2.4% | 6.0% |
| Pretty hard | 601 (91.5) | 56 (8.5) |  | 8.5% | 13.5% |
| Neither difficult nor easy | 1198 (92.8) | 93 (7.2) |  | 16.9% | 22.4% |
| Quite easy | 2369 (95.3) | 116 (4.7) |  | 33.4% | 27.9% |
| Easy | 1668 (96.6) | 59 (3.4) |  | 23.5% | 14.2% |
| Very easy | 973 (97.4) | 26 (2.6) |  | 13.7% | 6.2% |
| **…follow recommendations about when to engage in social activities, and when not to?** | | | | | |
| Very difficult | 101 (73.2) | 37 (26.8) | <0.001 | 1.4% | 8.9% |
| Hard | 168 (85.3) | 29 (14.7) |  | 2.4% | 7.0% |
| Pretty hard | 589 (90.2) | 64 (9.8) |  | 8.3% | 15.4% |
| Neither difficult nor easy | 1243 (92.6) | 100 (7.4) |  | 17.5% | 24.0% |
| Quite easy | 2264 (95.8) | 100 (4.2) |  | 31.9% | 24.0% |
| Easy | 1732 (96.8) | 58 (3.2) |  | 24.4% | 13.9% |
| Very easy | 1000 (97.3) | 28 (2.7) |  | 14.1% | 6.7% |
| ***Getting COVID probability and severity*** | | | | | |
| **How do you estimate your own probability of getting COVID-19 infection?** | | | | | |
| Very unlikely | 335 (88.9) | 42 (11.1) | <0.001 | 4.7% | 10.1% |
| Unlikely | 305 (88.4) | 40 (11.6) |  | 4.3% | 9.6% |
| Quite unlikely | 937 (94.1) | 59 (5.9) |  | 13.2% | 14.2% |
| Neither improbable nor probable | 3882 (95.1) | 202 (4.9) |  | 54.7% | 48.6% |
| Quite likely | 1111 (96.2) | 44 (3.8) |  | 15.7% | 10.6% |
| Likely | 336 (94.1) | 21 (5.9) |  | 4.7% | 5.0% |
| Very likely | 191 (96.0) | 8 (4.0) |  | 2.7% | 1.9% |
| **How susceptible do you consider yourself to an infection with COVID-19?** | | | | | |
| Totally vulnerable | 237 (96.3) | 9 (3.7) | <0.001 | 3.3% | 2.2% |
| vulnerable | 396 (95.7) | 18 (4.3) |  | 5.6% | 4.3% |
| Quite vulnerable | 1311 (96.3) | 50 (3.7) |  | 18.5% | 12.0% |
| Neither vulnerable nor invulnerable | 3859 (95.0) | 202 (5.0) |  | 54.4% | 48.6% |
| Quite invulnerable | 993 (91.7) | 90 (8.3) |  | 14.0% | 21.6% |
| Invulnerable | 154 (87.0) | 23 (13.0) |  | 2.2% | 5.5% |
| Totally invulnerable | 147 (86.0) | 24 (14.0) |  | 2.1% | 5.8% |
| **How severe would contracting COVID-19 be for you (how seriously ill do you think you will be)?** | | | | | |
| Not at all serious | 192 (82.8) | 40 (17.2) | <0.001 | 2.7% | 9.6% |
| Little serious | 390 (91.1) | 38 (8.9) |  | 5.5% | 9.1% |
| Not particularly bad | 1152 (93.7) | 78 (6.3) |  | 16.2% | 18.8% |
| Neither serious nor serious | 2177 (94.0) | 140 (6.0) |  | 30.7% | 33.7% |
| Serious enough | 2132 (96.1) | 86 (3.9) |  | 30.0% | 20.7% |
| Serious | 561 (97.6) | 14 (2.4) |  | 7.9% | 3.4% |
| Very serious | 493 (96.1) | 20 (3.9) |  | 6.9% | 4.8% |
| ***Preparedness and perceived self-efficacy*** | | | | | |
| **I know how to protect myself from coronavirus** | | | | | |
| Strongly disagree | 111 (94.1) | 7 (5.9) | <0.001 | 1.6% | 1.7% |
| Disagree | 49 (84.5) | 9 (15.5) |  | 0.7% | 2.2% |
| Quite disagree | 200 (93.5) | 14 (6.5) |  | 2.8% | 3.4% |
| Neither disagree nor agree | 1054 (91.7) | 96 (8.3) |  | 14.9% | 23.1% |
| Agree enough | 3028 (95.1) | 155 (4.9) |  | 42.7% | 37.3% |
| Agree | 1627 (95.8) | 72 (4.2) |  | 22.9% | 17.3% |
| Strongly agree | 1028 (94.2) | 63 (5.8) |  | 14.5% | 15.1% |
| **For me avoiding an infection with COVID-19 in the current situation is…** | | | | | |
| Very difficult | 121 (90.3) | 13 (9.7) | 0.071 | 1.7% | 3.1% |
| Hard | 147 (94.8) | 8 (5.2) |  | 2.1% | 1.9% |
| Pretty hard | 659 (94.4) | 39 (5.6) |  | 9.3% | 9.4% |
| Neither difficult nor easy | 3515 (95.1) | 182 (4.9) |  | 49.5% | 43.8% |
| Quite easy | 1893 (94.3) | 115 (5.7) |  | 26.7% | 27.6% |
| Easy | 513 (92.9) | 39 (7.1) |  | 7.2% | 9.4% |
| Very easy | 249 (92.6) | 20 (7.4) |  | 3.5% | 4.8% |
| ***Implementation of preventive behaviours*** | | | | | |
| **During the past 7 days, which measures you have performed to prevent to be infected by COVID-19?** | | | | | |
| **Frequently washed my hands with soap and water for at least 20 seconds** | | | | | |
| Never | 35 (60.3) | 23 (39.7) | <0.001 | 0.5% | 5.5% |
| Hardly ever | 78 (81.2) | 18 (18.8) |  | 1.1% | 4.3% |
| Very little | 131 (86.2) | 21 (13.8) |  | 1.8% | 5.0% |
| Some time | 534 (88.4) | 70 (11.6) |  | 7.5% | 16.8% |
| Often enough | 1233 (92.9) | 94 (7.1) |  | 17.4% | 22.6% |
| Often | 1805 (96.3) | 69 (3.7) |  | 25.4% | 16.6% |
| Very often | 3281 (96.4) | 121 (3.6) |  | 46.2% | 29.1% |
| **Avoided touching my eyes, nose and mouth with unwashed hands** | | | | | |
| Never | 83 (69.7) | 36 (30.3) | <0.001 | 1.2% | 8.7% |
| Hardly ever | 115 (85.8) | 19 (14.2) |  | 1.6% | 4.6% |
| Very little | 248 (88.9) | 31 (11.1) |  | 3.5% | 7.5% |
| Some time | 843 (90.1) | 93 (9.9) |  | 11.9% | 22.4% |
| Often enough | 1521 (94.8) | 84 (5.2) |  | 21.4% | 20.2% |
| Often | 1587 (96.1) | 65 (3.9) |  | 22.4% | 15.6% |
| Very often | 2700 (96.8) | 88 (3.2) |  | 38.0% | 21.2% |
| **Used disinfectants to clean hands when soap and water were not available** | | | | | |
| Never | 68 (63.6) | 39 (36.4) | <0.001 | 1.0% | 9.4% |
| Hardly ever | 69 (78.4) | 19 (21.6) |  | 1.0% | 4.6% |
| Very little | 100 (85.5) | 17 (14.5) |  | 1.4% | 4.1% |
| Some time | 529 (86.6) | 82 (13.4) |  | 7.5% | 19.7% |
| Often enough | 961 (92.9) | 74 (7.1) |  | 13.5% | 17.8% |
| Often | 1576 (95.6) | 72 (4.4) |  | 22.2% | 17.3% |
| Very often | 3794 (97.1) | 113 (2.9) |  | 53.5% | 27.2% |
| **Avoided a social event I wanted to attend** | | | | | |
| Never | 288 (82.8) | 60 (17.2) | <0.001 | 4.1% | 14.4% |
| Hardly ever | 129 (90.2) | 14 (9.8) |  | 1.8% | 3.4% |
| Very little | 160 (89.9) | 18 (10.1) |  | 2.3% | 4.3% |
| Some time | 605 (91.3) | 58 (8.7) |  | 8.5% | 13.9% |
| Often enough | 875 (92.9) | 67 (7.1) |  | 12.3% | 16.1% |
| Often | 1318 (95.0) | 70 (5.0) |  | 18.6% | 16.8% |
| Very often | 3722 (96.7) | 129 (3.3) |  | 52.4% | 31.0% |
| **Used medicines to prevent or treat COVID-19** | | | | | |
| Never | 5194 (95.3) | 254 (4.7) | <0.001 | 73.2% | 61.1% |
| Hardly ever | 503 (95.1) | 26 (4.9) |  | 7.1% | 6.2% |
| Very little | 380 (92.9) | 29 (7.1) |  | 5.4% | 7.0% |
| Some time | 369 (86.8) | 56 (13.2) |  | 5.2% | 13.5% |
| Often enough | 238 (89.5) | 28 (10.5) |  | 3.4% | 6.7% |
| Often | 188 (94.5) | 11 (5.5) |  | 2.6% | 2.6% |
| Very often | 225 (94.9) | 12 (5.1) |  | 3.2% | 2.9% |
| **Wore a mask in public** | | | | | |
| Never | 26 (74.3) | 9 (25.7) | <0.001 | 0.4% | 2.2% |
| Hardly ever | 19 (70.4) | 8 (29.6) |  | 0.3% | 1.9% |
| Very little | 52 (85.2) | 9 (14.8) |  | 0.7% | 2.2% |
| Some time | 130 (76.0) | 41 (24.0) |  | 1.8% | 9.9% |
| Often enough | 263 (86.2) | 42 (13.8) |  | 3.7% | 10.1% |
| Often | 486 (90.8) | 49 (9.2) |  | 6.8% | 11.8% |
| Very often | 6121 (96.0) | 258 (4.0) |  | 86.2% | 62.0% |
| **Ensured physical distancing in public** | | | | | |
| Never | 22 (52.4) | 20 (47.6) | <0.001 | 0.3% | 4.8% |
| Hardly ever | 32 (76.2) | 10 (23.8) |  | 0.5% | 2.4% |
| Very little | 59 (75.6) | 19 (24.4) |  | 0.8% | 4.6% |
| Some time | 238 (81.2) | 55 (18.8) |  | 3.4% | 13.2% |
| Often enough | 889 (91.6) | 81 (8.4) |  | 12.5% | 19.5% |
| Often | 1628 (95.1) | 83 (4.9) |  | 22.9% | 20.0% |
| Very often | 4229 (96.6) | 148 (3.4) |  | 59.6% | 35.6% |
| **Disinfected surfaces** | | | | | |
| Never | 126 (68.5) | 58 (31.5) | <0.001 | 1.8% | 13.9% |
| Hardly ever | 185 (92.0) | 16 (8.0) |  | 2.6% | 3.8% |
| Very little | 312 (91.2) | 30 (8.8) |  | 4.4% | 7.2% |
| Some time | 1093 (92.5) | 89 (7.5) |  | 15.4% | 21.4% |
| Often enough | 1547 (95.0) | 81 (5.0) |  | 21.8% | 19.5% |
| Often | 1589 (95.7) | 71 (4.3) |  | 22.4% | 17.1% |
| Very often | 2245 (96.9) | 71 (3.1) |  | 31.6% | 17.1% |
| **Stayed at home from work/school** | | | | | |
| Never | 1371 (94.0) | 87 (6.0) | 0.339 | 19.3% | 20.9% |
| Hardly ever | 498 (96.1) | 20 (3.9) |  | 7.0% | 4.8% |
| Very little | 328 (95.3) | 16 (4.7) |  | 4.6% | 3.8% |
| Some time | 740 (93.3) | 53 (6.7) |  | 10.4% | 12.7% |
| Often enough | 716 (93.7) | 48 (6.3) |  | 10.1% | 11.5% |
| Often | 627 (95.4) | 30 (4.6) |  | 8.8% | 7.2% |
| Very often | 1422 (94.7) | 80 (5.3) |  | 20.0% | 19.2% |
| Not applicable | 1395 (94.4) | 82 (5.6) |  | 19.7% | 19.7% |
| ***Perception and representation of threat*** | | | | | |
| **Please select the choice that is better express your opinion about the COVID-19** | | | | | |
| **How close or far away it from you** | | | | | |
| Close to me | 1145 (97.5) | 29 (2.5) | <0.001 | 16.1% | 7.0% |
| 2 | 1028 (96.6) | 36 (3.4) |  | 14.5% | 8.7% |
| 3 | 1060 (95.1) | 55 (4.9) |  | 14.9% | 13.2% |
| 4 | 2256 (94.9) | 120 (5.1) |  | 31.8% | 28.8% |
| 5 | 644 (92.1) | 55 (7.9) |  | 9.1% | 13.2% |
| 6 | 402 (90.3) | 43 (9.7) |  | 5.7% | 10.3% |
| Away from me | 562 (87.8) | 78 (12.2) |  | 7.9% | 18.8% |
| **Whether it is spreading slowly or fast** | | | | | |
| Slowly growing | 601 (90.1) | 66 (9.9) | <0.001 | 8.5% | 15.9% |
| 2 | 572 (93.6) | 39 (6.4) |  | 8.1% | 9.4% |
| 3 | 796 (93.9) | 52 (6.1) |  | 11.2% | 12.5% |
| 4 | 1727 (93.0) | 129 (7.0) |  | 24.3% | 31.0% |
| 5 | 1122 (94.8) | 62 (5.2) |  | 15.8% | 14.9% |
| 6 | 944 (96.3) | 36 (3.7) |  | 13.3% | 8.7% |
| Fast growing | 1335 (97.7) | 32 (2.3) |  | 18.8% | 7.7% |
| **How often you think about it** | | | | | |
| Something I always think about | 1687 (97.0) | 53 (3.0) | <0.001 | 23.8% | 12.7% |
| 2 | 1281 (96.9) | 41 (3.1) |  | 18.0% | 9.9% |
| 3 | 1205 (95.4) | 58 (4.6) |  | 17.0% | 13.9% |
| 4 | 1710 (94.0) | 109 (6.0) |  | 24.1% | 26.2% |
| 5 | 617 (91.1) | 60 (8.9) |  | 8.7% | 14.4% |
| 6 | 306 (88.7) | 39 (11.3) |  | 4.3% | 9.4% |
| Something I never think about | 291 (83.9) | 56 (16.1) |  | 4.1% | 13.5% |
| **How fear-inducing it is** | | | | | |
| Scary | 2374 (97.9) | 52 (2.1) | <0.001 | 33.5% | 12.5% |
| 2 | 1398 (96.9) | 45 (3.1) |  | 19.7% | 10.8% |
| 3 | 1187 (94.3) | 72 (5.7) |  | 16.7% | 17.3% |
| 4 | 1228 (92.6) | 98 (7.4) |  | 17.3% | 23.6% |
| 5 | 384 (92.8) | 30 (7.2) |  | 5.4% | 7.2% |
| 6 | 239 (85.7) | 40 (14.3) |  | 3.4% | 9.6% |
| Not scary | 287 (78.4) | 79 (21.6) |  | 4.0% | 19.0% |
| **How media infrom about it** | | | | | |
| Enlarged by the Media | 1121 (88.3) | 149 (11.7) | <0.001 | 15.8% | 35.8% |
| 2 | 668 (93.3) | 48 (6.7) |  | 9.4% | 11.5% |
| 3 | 763 (94.0) | 49 (6.0) |  | 10.8% | 11.8% |
| 4 | 1753 (95.9) | 75 (4.1) |  | 24.7% | 18.0% |
| 5 | 735 (94.4) | 44 (5.6) |  | 10.4% | 10.6% |
| 6 | 793 (96.2) | 31 (3.8) |  | 11.2% | 7.5% |
| Not exaggerated by the media | 1264 (98.4) | 20 (1.6) |  | 17.8% | 4.8% |
| **What is your perception about it** | | | | | |
| Something that makes me feel helpless | 1852 (96.9) | 59 (3.1) | <0.001 | 26.1% | 14.2% |
| 2 | 1016 (95.9) | 43 (4.1) |  | 14.3% | 10.3% |
| 3 | 858 (93.9) | 56 (6.1) |  | 12.1% | 13.5% |
| 4 | 1234 (93.3) | 89 (6.7) |  | 17.4% | 21.4% |
| 5 | 723 (93.8) | 48 (6.2) |  | 10.2% | 11.5% |
| 6 | 671 (94.9) | 36 (5.1) |  | 9.5% | 8.7% |
| Something that I am able to cope with with my behaviors | 743 (89.7) | 85 (10.3) |  | 10.5% | 20.4% |
| **How stressful it is** | | | | | |
| Stressful | 3658 (95.1) | 187 (4.9) | <0.001 | 51.5% | 45.0% |
| 2 | 1391 (95.7) | 62 (4.3) |  | 19.6% | 14.9% |
| 3 | 766 (95.2) | 39 (4.8) |  | 10.8% | 9.4% |
| 4 | 688 (92.2) | 58 (7.8) |  | 9.7% | 13.9% |
| 5 | 278 (90.8) | 28 (9.2) |  | 3.9% | 6.7% |
| 6 | 116 (89.9) | 13 (10.1) |  | 1.6% | 3.1% |
| Not stressful | 200 (87.3) | 29 (12.7) |  | 2.8% | 7.0% |
| ***Trust in sources of information on COVID-19*** | | | | | |
| **How much you trust to the information about the COVID-19 supplied by the following sources:** | | | | | |
| **Television** | | | | | |
| Do not trust at all | 670 (85.8) | 111 (14.2) | <0.001 | 9.4% | 26.7% |
| Trust very little | 670 (94.1) | 42 (5.9) |  | 9.4% | 10.1% |
| Trust little | 1121 (94.4) | 67 (5.6) |  | 15.8% | 16.1% |
| Neither do I trust nor do I trust | 2165 (95.1) | 111 (4.9) |  | 30.5% | 26.7% |
| Trust enough | 1862 (96.8) | 61 (3.2) |  | 26.2% | 14.7% |
| Trust a lot | 471 (96.5) | 17 (3.5) |  | 6.6% | 4.1% |
| Trust very much | 138 (95.2) | 7 (4.8) |  | 1.9% | 1.7% |
| **Newspapers** | | | | | |
| Do not trust at all | 637 (87.1) | 94 (12.9) | <0.001 | 9.0% | 22.6% |
| Trust very little | 612 (93.0) | 46 (7.0) |  | 8.6% | 11.1% |
| Trust little | 1103 (94.4) | 65 (5.6) |  | 15.5% | 15.6% |
| Neither do I trust nor do I trust | 2341 (94.8) | 128 (5.2) |  | 33.0% | 30.8% |
| Trust enough | 1858 (97.0) | 57 (3.0) |  | 26.2% | 13.7% |
| Trust a lot | 430 (95.8) | 19 (4.2) |  | 6.1% | 4.6% |
| Trust very much | 116 (94.3) | 7 (5.7) |  | 1.6% | 1.7% |
| **Health workers** | | | | | |
| Do not trust at all | 103 (73.6) | 37 (26.4) | <0.001 | 1.5% | 8.9% |
| Trust very little | 108 (83.7) | 21 (16.3) |  | 1.5% | 5.0% |
| Trust little | 298 (84.9) | 53 (15.1) |  | 4.2% | 12.7% |
| Neither do I trust nor do I trust | 949 (89.8) | 108 (10.2) |  | 13.4% | 26.0% |
| Trust enough | 2560 (95.5) | 120 (4.5) |  | 36.1% | 28.8% |
| Trust a lot | 2033 (97.6) | 49 (2.4) |  | 28.6% | 11.8% |
| Trust very much | 1046 (97.4) | 28 (2.6) |  | 14.7% | 6.7% |
| **Social media** | | | | | |
| Do not trust at all | 1461 (93.4) | 103 (6.6) | 0.236 | 20.6% | 24.8% |
| Trust very little | 1039 (94.4) | 62 (5.6) |  | 14.6% | 14.9% |
| Trust little | 1567 (95.7) | 71 (4.3) |  | 22.1% | 17.1% |
| Neither do I trust nor do I trust | 1932 (94.3) | 117 (5.7) |  | 27.2% | 28.1% |
| Trust enough | 823 (94.6) | 47 (5.4) |  | 11.6% | 11.3% |
| Trust a lot | 182 (94.3) | 11 (5.7) |  | 2.6% | 2.6% |
| Trust very much | 93 (94.9) | 5 (5.1) |  | 1.3% | 1.2% |
| **Radio** | | | | | |
| Do not trust at all | 455 (86.5) | 71 (13.5) | <0.001 | 6.4% | 17.1% |
| Trust very little | 461 (93.5) | 32 (6.5) |  | 6.5% | 7.7% |
| Trust little | 989 (92.9) | 76 (7.1) |  | 13.9% | 18.3% |
| Neither do I trust nor do I trust | 2586 (94.7) | 145 (5.3) |  | 36.4% | 34.9% |
| Trust enough | 2016 (96.7) | 69 (3.3) |  | 28.4% | 16.6% |
| Trust a lot | 457 (96.4) | 17 (3.6) |  | 6.4% | 4.1% |
| Trust very much | 133 (95.7) | 6 (4.3) |  | 1.9% | 1.4% |
| **Ministry of Health** | | | | | |
| Do not trust at all | 249 (77.3) | 73 (22.7) | <0.001 | 3.5% | 17.5% |
| Trust very little | 243 (86.8) | 37 (13.2) |  | 3.4% | 8.9% |
| Trust little | 464 (91.5) | 43 (8.5) |  | 6.5% | 10.3% |
| Neither do I trust nor do I trust | 1146 (92.3) | 95 (7.7) |  | 16.1% | 22.8% |
| Trust enough | 2294 (95.7) | 104 (4.3) |  | 32.3% | 25.0% |
| Trust a lot | 1764 (97.6) | 44 (2.4) |  | 24.9% | 10.6% |
| Trust very much | 937 (97.9) | 20 (2.1) |  | 13.2% | 4.8% |
| **Institute of Public Health/Center for Disease Control** | | | | | |
| Do not trust at all | 246 (77.4) | 72 (22.6) | <0.001 | 3.5% | 17.3% |
| Trust very little | 225 (85.9) | 37 (14.1) |  | 3.2% | 8.9% |
| Trust little | 483 (90.3) | 52 (9.7) |  | 6.8% | 12.5% |
| Neither do I trust nor do I trust | 1168 (92.7) | 92 (7.3) |  | 16.5% | 22.1% |
| Trust enough | 2260 (95.8) | 99 (4.2) |  | 31.8% | 23.8% |
| Trust a lot | 1718 (97.4) | 45 (2.6) |  | 24.2% | 10.8% |
| Trust very much | 997 (98.1) | 19 (1.9) |  | 14.0% | 4.6% |
| **Celebrities and social media influencers** | | | | | |
| Do not trust at all | 2122 (92.9) | 162 (7.1) | 0.009 | 29.9% | 38.9% |
| Trust very little | 958 (95.1) | 49 (4.9) |  | 13.5% | 11.8% |
| Trust little | 1400 (95.2) | 71 (4.8) |  | 19.7% | 17.1% |
| Neither do I trust nor do I trust | 1801 (95.3) | 89 (4.7) |  | 25.4% | 21.4% |
| Trust enough | 573 (95.3) | 28 (4.7) |  | 8.1% | 6.7% |
| Trust a lot | 159 (94.1) | 10 (5.9) |  | 2.2% | 2.4% |
| Trust very much | 84 (92.3) | 7 (7.7) |  | 1.2% | 1.7% |
| **World Health Organization (WHO)** | | | | | |
| Do not trust at all | 306 (79.9) | 77 (20.1) | <0.001 | 4.3% | 18.5% |
| Trust very little | 241 (85.5) | 41 (14.5) |  | 3.4% | 9.9% |
| Trust little | 569 (92.8) | 44 (7.2) |  | 8.0% | 10.6% |
| Neither do I trust nor do I trust | 1251 (93.0) | 94 (7.0) |  | 17.6% | 22.6% |
| Trust enough | 2215 (95.7) | 99 (4.3) |  | 31.2% | 23.8% |
| Trust a lot | 1611 (97.5) | 42 (2.5) |  | 22.7% | 10.1% |
| Trust very much | 904 (97.9) | 19 (2.1) |  | 12.7% | 4.6% |
| **COVID-19 Hotlines** | | | | | |
| Do not trust at all | 406 (84.8) | 73 (15.2) | <0.001 | 5.7% | 17.5% |
| Trust very little | 328 (90.4) | 35 (9.6) |  | 4.6% | 8.4% |
| Trust little | 724 (92.1) | 62 (7.9) |  | 10.2% | 14.9% |
| Neither do I trust nor do I trust | 2442 (94.6) | 140 (5.4) |  | 34.4% | 33.7% |
| Trust enough | 1972 (96.5) | 72 (3.5) |  | 27.8% | 17.3% |
| Trust a lot | 899 (97.5) | 23 (2.5) |  | 12.7% | 5.5% |
| Trust very much | 326 (96.7) | 11 (3.3) |  | 4.6% | 2.6% |
| **National COVID-19 information website** | | | | | |
| Do not trust at all | 241 (79.5) | 62 (20.5) | <0.001 | 3.4% | 14.9% |
| Trust very little | 263 (86.8) | 40 (13.2) |  | 3.7% | 9.6% |
| Trust little | 562 (90.4) | 60 (9.6) |  | 7.9% | 14.4% |
| Neither do I trust nor do I trust | 1640 (93.6) | 113 (6.4) |  | 23.1% | 27.2% |
| Trust enough | 2461 (96.4) | 92 (3.6) |  | 34.7% | 22.1% |
| Trust a lot | 1401 (97.7) | 33 (2.3) |  | 19.7% | 7.9% |
| Trust very much | 529 (97.1) | 16 (2.9) |  | 7.5% | 3.8% |
| ***Use of sources of information on COVID-19*** | | | | | |
| **How often do you use COVID-19 related information from the following sources:** | | | | | |
| **Television** | | | | | |
| Never | 580 (88.8) | 73 (11.2) | <0.001 | 8.2% | 17.5% |
| Hardly ever | 504 (93.9) | 33 (6.1) |  | 7.1% | 7.9% |
| Very little | 751 (94.6) | 43 (5.4) |  | 10.6% | 10.3% |
| Some time | 1685 (93.6) | 116 (6.4) |  | 23.7% | 27.9% |
| Often enough | 1913 (96.4) | 72 (3.6) |  | 27.0% | 17.3% |
| Often | 940 (94.7) | 53 (5.3) |  | 13.2% | 12.7% |
| Very often | 724 (96.5) | 26 (3.5) |  | 10.2% | 6.2% |
| **Newspapers** | | | | | |
| Never | 1289 (91.4) | 121 (8.6) | <0.001 | 18.2% | 29.1% |
| Hardly ever | 851 (95.1) | 44 (4.9) |  | 12.0% | 10.6% |
| Very little | 1062 (95.0) | 56 (5.0) |  | 15.0% | 13.5% |
| Some time | 2019 (94.3) | 122 (5.7) |  | 28.4% | 29.3% |
| Often enough | 1156 (96.7) | 40 (3.3) |  | 16.3% | 9.6% |
| Often | 473 (95.2) | 24 (4.8) |  | 6.7% | 5.8% |
| Very often | 247 (96.5) | 9 (3.5) |  | 3.5% | 2.2% |
| **Health workers** | | | | | |
| Never | 678 (88.3) | 90 (11.7) | <0.001 | 9.6% | 21.6% |
| Hardly ever | 586 (92.9) | 45 (7.1) |  | 8.3% | 10.8% |
| Very little | 722 (93.0) | 54 (7.0) |  | 10.2% | 13.0% |
| Some time | 2207 (95.0) | 115 (5.0) |  | 31.1% | 27.6% |
| Often enough | 1535 (95.5) | 72 (4.5) |  | 21.6% | 17.3% |
| Often | 896 (97.0) | 28 (3.0) |  | 12.6% | 6.7% |
| Very often | 473 (97.5) | 12 (2.5) |  | 6.7% | 2.9% |
| **Social media** | | | | | |
| Never | 1974 (94.4) | 116 (5.6) | 0.033 | 27.8% | 27.9% |
| Hardly ever | 1047 (95.4) | 50 (4.6) |  | 14.8% | 12.0% |
| Very little | 1076 (95.5) | 51 (4.5) |  | 15.2% | 12.3% |
| Some time | 1585 (93.5) | 110 (6.5) |  | 22.3% | 26.4% |
| Often enough | 891 (94.9) | 48 (5.1) |  | 12.6% | 11.5% |
| Often | 334 (91.5) | 31 (8.5) |  | 4.7% | 7.5% |
| Very often | 190 (95.0) | 10 (5.0) |  | 2.7% | 2.4% |
| **Radio** | | | | | |
| Never | 1387 (92.3) | 116 (7.7) | 0.003 | 19.5% | 27.9% |
| Hardly ever | 969 (94.1) | 61 (5.9) |  | 13.7% | 14.7% |
| Very little | 1106 (94.9) | 59 (5.1) |  | 15.6% | 14.2% |
| Some time | 1994 (95.4) | 96 (4.6) |  | 28.1% | 23.1% |
| Often enough | 1037 (95.3) | 51 (4.7) |  | 14.6% | 12.3% |
| Often | 399 (94.5) | 23 (5.5) |  | 5.6% | 5.5% |
| Very often | 205 (95.3) | 10 (4.7) |  | 2.9% | 2.4% |
| **Ministry of Health** | | | | | |
| Never | 712 (86.0) | 116 (14.0) | <0.001 | 10.0% | 27.9% |
| Hardly ever | 472 (93.3) | 34 (6.7) |  | 6.7% | 8.2% |
| Very little | 661 (92.7) | 52 (7.3) |  | 9.3% | 12.5% |
| Some time | 2088 (94.7) | 118 (5.3) |  | 29.4% | 28.4% |
| Often enough | 1592 (96.7) | 54 (3.3) |  | 22.4% | 13.0% |
| Often | 1018 (97.3) | 28 (2.7) |  | 14.3% | 6.7% |
| Very often | 554 (97.5) | 14 (2.5) |  | 7.8% | 3.4% |
| **Institute of Public Health/Center for Disease Control** | | | | | |
| Never | 803 (86.1) | 130 (13.9) | <0.001 | 11.3% | 31.2% |
| Hardly ever | 549 (91.7) | 50 (8.3) |  | 7.7% | 12.0% |
| Very little | 703 (94.2) | 43 (5.8) |  | 9.9% | 10.3% |
| Some time | 2053 (95.4) | 99 (4.6) |  | 28.9% | 23.8% |
| Often enough | 1631 (95.9) | 70 (4.1) |  | 23.0% | 16.8% |
| Often | 868 (98.9) | 10 (1.1) |  | 12.2% | 2.4% |
| Very often | 490 (97.2) | 14 (2.8) |  | 6.9% | 3.4% |
| **Celebrities and social media influencers** | | | | | |
| Never | 3988 (94.4) | 236 (5.6) | <0.001 | 56.2% | 56.7% |
| Hardly ever | 993 (96.0) | 41 (4.0) |  | 14.0% | 9.9% |
| Very little | 797 (96.1) | 32 (3.9) |  | 11.2% | 7.7% |
| Some time | 692 (92.4) | 57 (7.6) |  | 9.8% | 13.7% |
| Often enough | 345 (91.0) | 34 (9.0) |  | 4.9% | 8.2% |
| Often | 179 (95.2) | 9 (4.8) |  | 2.5% | 2.2% |
| Very often | 103 (93.6) | 7 (6.4) |  | 1.5% | 1.7% |
| **World Health Organization (WHO)** | | | | | |
| Never | 923 (87.6) | 131 (12.4) | <0.001 | 13.0% | 31.5% |
| Hardly ever | 596 (93.4) | 42 (6.6) |  | 8.4% | 10.1% |
| Very little | 829 (94.0) | 53 (6.0) |  | 11.7% | 12.7% |
| Some time | 2067 (95.3) | 102 (4.7) |  | 29.1% | 24.5% |
| Often enough | 1425 (96.2) | 57 (3.8) |  | 20.1% | 13.7% |
| Often | 785 (98.0) | 16 (2.0) |  | 11.1% | 3.8% |
| Very often | 472 (96.9) | 15 (3.1) |  | 6.7% | 3.6% |
| **COVID-19 Hotlines** | | | | | |
| Never | 3571 (93.9) | 230 (6.1) | 0.051 | 50.3% | 55.3% |
| Hardly ever | 1026 (96.3) | 39 (3.7) |  | 14.5% | 9.4% |
| Very little | 813 (95.3) | 40 (4.7) |  | 11.5% | 9.6% |
| Some time | 865 (94.3) | 52 (5.7) |  | 12.2% | 12.5% |
| Often enough | 450 (93.8) | 30 (6.2) |  | 6.3% | 7.2% |
| Often | 243 (92.7) | 19 (7.3) |  | 3.4% | 4.6% |
| Very often | 129 (95.6) | 6 (4.4) |  | 1.8% | 1.4% |
| **National COVID-19 information website** | | | | | |
| Never | 934 (89.7) | 107 (10.3) | <0.001 | 13.2% | 25.7% |
| Hardly ever | 626 (93.0) | 47 (7.0) |  | 8.8% | 11.3% |
| Very little | 794 (94.2) | 49 (5.8) |  | 11.2% | 11.8% |
| Some time | 2164 (95.0) | 113 (5.0) |  | 30.5% | 27.2% |
| Often enough | 1433 (95.9) | 61 (4.1) |  | 20.2% | 14.7% |
| Often | 767 (96.1) | 31 (3.9) |  | 10.8% | 7.5% |
| Very often | 379 (97.9) | 8 (2.1) |  | 5.3% | 1.9% |
| ***Frequency of information*** | | | | | |
| **How often do you seek information about COVID-19** | | | | | |
| Never | 387 (84.9) | 69 (15.1) | <0.001 | 5.5% | 16.6% |
| Hardly ever | 554 (90.5) | 58 (9.5) |  | 7.8% | 13.9% |
| Very little | 808 (94.4) | 48 (5.6) |  | 11.4% | 11.5% |
| Some time | 2409 (94.5) | 140 (5.5) |  | 33.9% | 33.7% |
| Often enough | 1781 (96.7) | 61 (3.3) |  | 25.1% | 14.7% |
| Often | 670 (97.0) | 21 (3.0) |  | 9.4% | 5.0% |
| Very often | 488 (96.3) | 19 (3.7) |  | 6.9% | 4.6% |
| ***Trust in Institutions*** | | | | | |
| **How much trust you have that the following institutions or persons can effectively manage the COVID-19?** | | | | | |
| **Your family doctor** | | | | | |
| Do not trust at all | 189 (84.8) | 34 (15.2) | <0.001 | 2.7% | 8.2% |
| Trust very little | 204 (90.7) | 21 (9.3) |  | 2.9% | 5.0% |
| Trust little | 428 (91.3) | 41 (8.7) |  | 6.0% | 9.9% |
| Neither do I trust nor do I trust | 1149 (91.7) | 104 (8.3) |  | 16.2% | 25.0% |
| Trust enough | 2722 (95.0) | 143 (5.0) |  | 38.4% | 34.4% |
| Trust a lot | 1627 (97.0) | 51 (3.0) |  | 22.9% | 12.3% |
| Trust very much | 778 (97.2) | 22 (2.8) |  | 11.0% | 5.3% |
| **Hospitals** | | | | | |
| Do not trust at all | 153 (79.3) | 40 (20.7) | <0.001 | 2.2% | 9.6% |
| Trust very little | 204 (86.1) | 33 (13.9) |  | 2.9% | 7.9% |
| Trust little | 527 (92.1) | 45 (7.9) |  | 7.4% | 10.8% |
| Neither do I trust nor do I trust | 1214 (92.7) | 95 (7.3) |  | 17.1% | 22.8% |
| Trust enough | 2715 (95.6) | 126 (4.4) |  | 38.3% | 30.3% |
| Trust a lot | 1640 (96.7) | 56 (3.3) |  | 23.1% | 13.5% |
| Trust very much | 644 (96.8) | 21 (3.2) |  | 9.1% | 5.0% |
| **Ministry of Health** | | | | | |
| Do not trust at all | 319 (79.4) | 83 (20.6) | <0.001 | 4.5% | 20.0% |
| Trust very little | 232 (86.6) | 36 (13.4) |  | 3.3% | 8.7% |
| Trust little | 528 (92.3) | 44 (7.7) |  | 7.4% | 10.6% |
| Neither do I trust nor do I trust | 1380 (94.1) | 86 (5.9) |  | 19.4% | 20.7% |
| Trust enough | 2634 (95.8) | 116 (4.2) |  | 37.1% | 27.9% |
| Trust a lot | 1414 (97.4) | 38 (2.6) |  | 19.9% | 9.1% |
| Trust very much | 590 (97.8) | 13 (2.2) |  | 8.3% | 3.1% |
| **Institute of Public Health /Center for disease Control** | | | | | |
| Do not trust at all | 266 (77.3) | 78 (22.7) | <0.001 | 3.7% | 18.8% |
| Trust very little | 221 (89.1) | 27 (10.9) |  | 3.1% | 6.5% |
| Trust little | 504 (92.8) | 39 (7.2) |  | 7.1% | 9.4% |
| Neither do I trust nor do I trust | 1309 (92.4) | 107 (7.6) |  | 18.4% | 25.7% |
| Trust enough | 2617 (96.0) | 108 (4.0) |  | 36.9% | 26.0% |
| Trust a lot | 1507 (97.2) | 44 (2.8) |  | 21.2% | 10.6% |
| Trust very much | 673 (98.1) | 13 (1.9) |  | 9.5% | 3.1% |
| **Schools** | | | | | |
| Do not trust at all | 497 (90.2) | 54 (9.8) | <0.001 | 7.0% | 13.0% |
| Trust very little | 569 (93.3) | 41 (6.7) |  | 8.0% | 9.9% |
| Trust little | 1088 (95.0) | 57 (5.0) |  | 15.3% | 13.7% |
| Neither do I trust nor do I trust | 2103 (94.7) | 118 (5.3) |  | 29.6% | 28.4% |
| Trust enough | 1982 (95.4) | 95 (4.6) |  | 27.9% | 22.8% |
| Trust a lot | 640 (94.7) | 36 (5.3) |  | 9.0% | 8.7% |
| Trust very much | 218 (93.6) | 15 (6.4) |  | 3.1% | 3.6% |
| **Public transportation companies** | | | | | |
| Do not trust at all | 1312 (93.9) | 85 (6.1) | 0.065 | 18.5% | 20.4% |
| Trust very little | 1022 (94.9) | 55 (5.1) |  | 14.4% | 13.2% |
| Trust little | 1752 (95.7) | 78 (4.3) |  | 24.7% | 18.8% |
| Neither do I trust nor do I trust | 1794 (94.2) | 111 (5.8) |  | 25.3% | 26.7% |
| Trust enough | 861 (93.1) | 64 (6.9) |  | 12.1% | 15.4% |
| Trust a lot | 252 (94.7) | 14 (5.3) |  | 3.6% | 3.4% |
| Trust very much | 104 (92.0) | 9 (8.0) |  | 1.5% | 2.2% |
| **Police** | | | | | |
| Do not trust at all | 282 (83.4) | 56 (16.6) | <0.001 | 4.0% | 13.5% |
| Trust very little | 322 (89.2) | 39 (10.8) |  | 4.5% | 9.4% |
| Trust little | 697 (93.8) | 46 (6.2) |  | 9.8% | 11.1% |
| Neither do I trust nor do I trust | 1842 (94.1) | 115 (5.9) |  | 26.0% | 27.6% |
| Trust enough | 2485 (95.7) | 111 (4.3) |  | 35.0% | 26.7% |
| Trust a lot | 989 (96.6) | 35 (3.4) |  | 13.9% | 8.4% |
| Trust very much | 480 (97.2) | 14 (2.8) |  | 6.8% | 3.4% |
| **Your employer** | | | | | |
| Do not trust at all | 380 (90.0) | 42 (10.0) | 0.003 | 5.4% | 10.1% |
| Trust very little | 228 (92.7) | 18 (7.3) |  | 3.2% | 4.3% |
| Trust little | 481 (94.5) | 28 (5.5) |  | 6.8% | 6.7% |
| Neither do I trust nor do I trust | 1293 (94.4) | 77 (5.6) |  | 18.2% | 18.5% |
| Trust enough | 1536 (94.9) | 83 (5.1) |  | 21.6% | 20.0% |
| Trust a lot | 812 (95.9) | 35 (4.1) |  | 11.4% | 8.4% |
| Trust very much | 518 (94.4) | 31 (5.6) |  | 7.3% | 7.5% |
| Not applicable | 1849 (94.8) | 102 (5.2) |  | 26.1% | 24.5% |
| **Your church/place of worship** | | | | | |
| Do not trust at all | 969 (91.0) | 96 (9.0) | <0.001 | 13.7% | 23.1% |
| Trust very little | 367 (95.1) | 19 (4.9) |  | 5.2% | 4.6% |
| Trust little | 637 (95.8) | 28 (4.2) |  | 9.0% | 6.7% |
| Neither do I trust nor do I trust | 1844 (95.2) | 92 (4.8) |  | 26.0% | 22.1% |
| Trust enough | 1278 (94.7) | 72 (5.3) |  | 18.0% | 17.3% |
| Trust a lot | 535 (95.5) | 25 (4.5) |  | 7.5% | 6.0% |
| Trust very much | 333 (94.6) | 19 (5.4) |  | 4.7% | 4.6% |
| Not applicable | 1134 (94.6) | 65 (5.4) |  | 16.0% | 15.6% |
| ***Belief in conspiracies*** | | | | | |
| **What do you think about the decisions made in Italy during the epidemic?** | | | | | |
| **Many very important things happen in the world, which the public is never informed about** | | | | | |
| Completely false | 159 (94.1) | 10 (5.9) | <0.001 | 2.2% | 2.4% |
| False | 223 (96.1) | 9 (3.9) |  | 3.1% | 2.2% |
| Fairly false | 410 (93.8) | 27 (6.2) |  | 5.8% | 6.5% |
| Neither false nor true | 1502 (94.9) | 81 (5.1) |  | 21.2% | 19.5% |
| True enough | 2374 (95.3) | 116 (4.7) |  | 33.5% | 27.9% |
| True | 1217 (95.2) | 61 (4.8) |  | 17.1% | 14.7% |
| Completely true | 1212 (91.5) | 112 (8.5) |  | 17.1% | 26.9% |
| **Politicians usually do not tell us the true motives for their decisions** | | | | | |
| Completely false | 147 (93.6) | 10 (6.4) | <0.001 | 2.1% | 2.4% |
| False | 194 (95.6) | 9 (4.4) |  | 2.7% | 2.2% |
| Fairly false | 325 (93.7) | 22 (6.3) |  | 4.6% | 5.3% |
| Neither false nor true | 1429 (94.8) | 78 (5.2) |  | 20.1% | 18.8% |
| True enough | 2282 (95.6) | 106 (4.4) |  | 32.2% | 25.5% |
| True | 1349 (95.3) | 67 (4.7) |  | 19.0% | 16.1% |
| Completely true | 1371 (91.7) | 124 (8.3) |  | 19.3% | 29.8% |
| **Government agencies closely monitor all citizens** | | | | | |
| Completely false | 471 (95.5) | 22 (4.5) | <0.001 | 6.6% | 5.3% |
| False | 614 (96.5) | 22 (3.5) |  | 8.7% | 5.3% |
| Fairly false | 915 (95.4) | 44 (4.6) |  | 12.9% | 10.6% |
| Neither false nor true | 2207 (94.7) | 124 (5.3) |  | 31.1% | 29.8% |
| True enough | 1679 (94.6) | 95 (5.4) |  | 23.7% | 22.8% |
| True | 695 (94.3) | 42 (5.7) |  | 9.8% | 10.1% |
| Completely true | 516 (88.5) | 67 (11.5) |  | 7.3% | 16.1% |
| **Events which superficially seem to lack a connection are often the result of secret activities** | | | | | |
| Completely false | 552 (96.3) | 21 (3.7) | <0.001 | 7.8% | 5.0% |
| False | 619 (96.4) | 23 (3.6) |  | 8.7% | 5.5% |
| Fairly false | 739 (95.7) | 33 (4.3) |  | 10.4% | 7.9% |
| Neither false nor true | 2649 (95.1) | 137 (4.9) |  | 37.3% | 32.9% |
| True enough | 1466 (93.7) | 99 (6.3) |  | 20.7% | 23.8% |
| True | 627 (94.1) | 39 (5.9) |  | 8.8% | 9.4% |
| Completely true | 445 (87.4) | 64 (12.6) |  | 6.3% | 15.4% |
| **There are secret organizations that greatly influence political** | | | | | |
| Completely false | 606 (97.4) | 16 (2.6) | <0.001 | 8.5% | 3.8% |
| False | 576 (95.2) | 29 (4.8) |  | 8.1% | 7.0% |
| Fairly false | 668 (95.8) | 29 (4.2) |  | 9.4% | 7.0% |
| Neither false nor true | 2142 (94.9) | 115 (5.1) |  | 30.2% | 27.6% |
| True enough | 1666 (94.1) | 105 (5.9) |  | 23.5% | 25.2% |
| True | 757 (94.5) | 44 (5.5) |  | 10.7% | 10.6% |
| Completely true | 682 (89.7) | 78 (10.3) |  | 9.6% | 18.8% |
| ***Resilience*** | | | | | |
| **Please describe your experience during the COVID-10 epidemic:** | | | | | |
| **I have a hard time making it through stressful events** | | | | | |
| Strongly disagree | 408 (91.5) | 38 (8.5) | 0.101 | 5.7% | 9.1% |
| Disagree | 779 (94.9) | 42 (5.1) |  | 11.0% | 10.1% |
| Quite disagree | 1277 (95.2) | 65 (4.8) |  | 18.0% | 15.6% |
| Neither disagree nor agree | 2209 (94.3) | 133 (5.7) |  | 31.1% | 32.0% |
| Agree enough | 1587 (94.6) | 90 (5.4) |  | 22.4% | 21.6% |
| Agree | 506 (95.3) | 25 (4.7) |  | 7.1% | 6.0% |
| Strongly agree | 331 (93.5) | 23 (6.5) |  | 4.7% | 5.5% |
| **It does not take me long to recover from a stressful event** | | | | | |
| Strongly disagree | 356 (90.8) | 36 (9.2) | <0.001 | 5.0% | 8.7% |
| Disagree | 619 (97.2) | 18 (2.8) |  | 8.7% | 4.3% |
| Quite disagree | 1135 (95.5) | 53 (4.5) |  | 16.0% | 12.7% |
| Neither disagree nor agree | 2125 (93.4) | 149 (6.6) |  | 29.9% | 35.8% |
| Agree enough | 1916 (95.2) | 96 (4.8) |  | 27.0% | 23.1% |
| Agree | 648 (94.7) | 36 (5.3) |  | 9.1% | 8.7% |
| Strongly agree | 298 (91.4) | 28 (8.6) |  | 4.2% | 6.7% |
| **It is hard for me to snap back when something bad happens** | | | | | |
| Strongly disagree | 321 (89.9) | 36 (10.1) | <0.001 | 4.5% | 8.7% |
| Disagree | 662 (94.6) | 38 (5.4) |  | 9.3% | 9.1% |
| Quite disagree | 1166 (94.9) | 63 (5.1) |  | 16.4% | 15.1% |
| Neither disagree nor agree | 2100 (93.6) | 144 (6.4) |  | 29.6% | 34.6% |
| Agree enough | 1836 (95.5) | 86 (4.5) |  | 25.9% | 20.7% |
| Agree | 595 (95.8) | 26 (4.2) |  | 8.4% | 6.2% |
| Strongly agree | 417 (94.8) | 23 (5.2) |  | 5.9% | 5.5% |
| ***Testing and tracing*** | | | | | |
| **If you have been in contact with someone who tested positive for COVID-19 and have no symptoms yourself – will you get tested if you have the opportunity?** | | | | | |
| I would take the test for sure | 6502 (97.0) | 202 (3.0) | <0.001 | 91.6% | 48.6% |
| I probably wouldn't take the test | 595 (73.5) | 214 (26.5) |  | 8.4% | 51.4% |
| ***Fairness of measure adopted*** | | | | | |
| **Please express your opinion regarding the decisions made in Italy to reduce the spread of COVID-19:** | | | | | |
| **I think the decisions are fair** | | | | | |
| Strongly disagree | 424 (81.5) | 96 (18.5) | <0.001 | 6.0% | 23.1% |
| Disagree | 360 (91.6) | 33 (8.4) |  | 5.1% | 7.9% |
| Quite disagree | 853 (93.4) | 60 (6.6) |  | 12.0% | 14.4% |
| Neither disagree nor agree | 1738 (93.3) | 125 (6.7) |  | 24.5% | 30.0% |
| Agree enough | 2196 (96.8) | 73 (3.2) |  | 30.9% | 17.5% |
| Agree | 943 (97.9) | 20 (2.1) |  | 13.3% | 4.8% |
| Strongly agree | 583 (98.5) | 9 (1.5) |  | 8.2% | 2.2% |
| **I would convince others that the decisions are right** | | | | | |
| Strongly disagree | 409 (81.6) | 92 (18.4) | <0.001 | 5.8% | 22.1% |
| Disagree | 348 (90.6) | 36 (9.4) |  | 4.9% | 8.7% |
| Quite disagree | 665 (93.9) | 43 (6.1) |  | 9.4% | 10.3% |
| Neither disagree nor agree | 2105 (93.9) | 137 (6.1) |  | 29.7% | 32.9% |
| Agree enough | 1987 (96.2) | 78 (3.8) |  | 28.0% | 18.8% |
| Agree | 1001 (97.8) | 23 (2.2) |  | 14.1% | 5.5% |
| Strongly agree | 582 (98.8) | 7 (1.2) |  | 8.2% | 1.7% |
| ***Restriction lifting or enforcement*** | | | | | |
| **Please express your opinion about the following phrases:** | | | | | |
| **In the event of an outbreak it’s appropriate to avoid certain people on the basis of their ethnicity** | | | | | |
| Strongly disagree | 2467 (94.9) | 133 (5.1) | <0.001 | 34.8% | 32.0% |
| Disagree | 1143 (96.9) | 37 (3.1) |  | 16.1% | 8.9% |
| Quite disagree | 730 (95.3) | 36 (4.7) |  | 10.3% | 8.7% |
| Neither disagree nor agree | 1413 (91.9) | 124 (8.1) |  | 19.9% | 29.8% |
| Agree enough | 605 (93.5) | 42 (6.5) |  | 8.5% | 10.1% |
| Agree | 389 (93.5) | 27 (6.5) |  | 5.5% | 6.5% |
| Strongly agree | 350 (95.4) | 17 (4.6) |  | 4.9% | 4.1% |
| **I think that the restrictions currently being implemented are greatly exaggerated** | | | | | |
| Strongly disagree | 1099 (97.4) | 29 (2.6) | <0.001 | 15.5% | 7.0% |
| Disagree | 1242 (97.9) | 26 (2.1) |  | 17.5% | 6.2% |
| Quite disagree | 1245 (97.3) | 35 (2.7) |  | 17.5% | 8.4% |
| Neither disagree nor agree | 1672 (94.1) | 105 (5.9) |  | 23.6% | 25.2% |
| Agree enough | 968 (92.4) | 80 (7.6) |  | 13.6% | 19.2% |
| Agree | 453 (92.3) | 38 (7.7) |  | 6.4% | 9.1% |
| Strongly agree | 418 (80.2) | 103 (19.8) |  | 5.9% | 24.8% |
| **The government should be allowed to force people into self-isolation if they have been in contact with someone who was infected** | | | | | |
| Strongly disagree | 203 (69.8) | 88 (30.2) | <0.001 | 2.9% | 21.2% |
| Disagree | 167 (85.6) | 28 (14.4) |  | 2.4% | 6.7% |
| Quite disagree | 328 (89.1) | 40 (10.9) |  | 4.6% | 9.6% |
| Neither disagree nor agree | 1462 (92.3) | 122 (7.7) |  | 20.6% | 29.3% |
| Agree enough | 2090 (96.2) | 83 (3.8) |  | 29.4% | 20.0% |
| Agree | 1379 (97.7) | 32 (2.3) |  | 19.4% | 7.7% |
| Strongly agree | 1468 (98.5) | 23 (1.5) |  | 20.7% | 5.5% |
| **More tests for coronavirus infection should be carried out in the population** | | | | | |
| Strongly disagree | 105 (64.0) | 59 (36.0) | <0.001 | 1.5% | 14.2% |
| Disagree | 110 (84.0) | 21 (16.0) |  | 1.5% | 5.0% |
| Quite disagree | 198 (83.2) | 40 (16.8) |  | 2.8% | 9.6% |
| Neither disagree nor agree | 1426 (91.6) | 131 (8.4) |  | 20.1% | 31.5% |
| Agree enough | 2296 (96.1) | 92 (3.9) |  | 32.4% | 22.1% |
| Agree | 1450 (97.4) | 38 (2.6) |  | 20.4% | 9.1% |
| Strongly agree | 1512 (97.7) | 35 (2.3) |  | 21.3% | 8.4% |
| **I am worried that the pandemic will have economic consequences for me in the future** | | | | | |
| Strongly disagree | 163 (86.7) | 25 (13.3) | <0.001 | 2.3% | 6.0% |
| Disagree | 268 (96.1) | 11 (3.9) |  | 3.8% | 2.6% |
| Quite disagree | 407 (94.2) | 25 (5.8) |  | 5.7% | 6.0% |
| Neither disagree nor agree | 1807 (94.2) | 112 (5.8) |  | 25.5% | 26.9% |
| Agree enough | 1988 (95.8) | 87 (4.2) |  | 28.0% | 20.9% |
| Agree | 1002 (94.9) | 54 (5.1) |  | 14.1% | 13.0% |
| Strongly agree | 1462 (93.5) | 102 (6.5) |  | 20.6% | 24.5% |
| **Please indicate your opinion about the following anti-epidemic measures:** | | | | | |
| **Compulsory face masks in closed public spaces** | | | | | |
| Strongly disagree | 220 (73.8) | 78 (26.2) | <0.001 | 3.1% | 18.8% |
| Disagree | 152 (87.4) | 22 (12.6) |  | 2.1% | 5.3% |
| Quite disagree | 277 (87.1) | 41 (12.9) |  | 3.9% | 9.9% |
| Neither disagree nor agree | 699 (88.7) | 89 (11.3) |  | 9.8% | 21.4% |
| Agree enough | 994 (94.5) | 58 (5.5) |  | 14.0% | 13.9% |
| Agree | 1294 (95.9) | 56 (4.1) |  | 18.2% | 13.5% |
| Strongly agree | 3461 (98.0) | 72 (2.0) |  | 48.8% | 17.3% |
| **Restricting restaurants to outside spaces** | | | | | |
| Strongly disagree | 683 (84.2) | 128 (15.8) | <0.001 | 9.6% | 30.8% |
| Disagree | 502 (93.0) | 38 (7.0) |  | 7.1% | 9.1% |
| Quite disagree | 884 (94.8) | 48 (5.2) |  | 12.5% | 11.5% |
| Neither disagree nor agree | 1293 (93.9) | 84 (6.1) |  | 18.2% | 20.2% |
| Agree enough | 1380 (95.3) | 68 (4.7) |  | 19.4% | 16.3% |
| Agree | 1165 (97.7) | 28 (2.3) |  | 16.4% | 6.7% |
| Strongly agree | 1190 (98.2) | 22 (1.8) |  | 16.8% | 5.3% |
| **Distance learning in schools** | | | | | |
| Strongly disagree | 561 (84.2) | 105 (15.8) | <0.001 | 7.9% | 25.2% |
| Disagree | 485 (94.5) | 28 (5.5) |  | 6.8% | 6.7% |
| Quite disagree | 790 (94.8) | 43 (5.2) |  | 11.1% | 10.3% |
| Neither disagree nor agree | 1273 (92.8) | 99 (7.2) |  | 17.9% | 23.8% |
| Agree enough | 1457 (95.7) | 66 (4.3) |  | 20.5% | 15.9% |
| Agree | 1057 (96.5) | 38 (3.5) |  | 14.9% | 9.1% |
| Strongly agree | 1474 (97.6) | 37 (2.4) |  | 20.8% | 8.9% |
| **Curfew from 22:00 to 5:00** | | | | | |
| Strongly disagree | 566 (81.7) | 127 (18.3) | <0.001 | 8.0% | 30.5% |
| Disagree | 468 (93.2) | 34 (6.8) |  | 6.6% | 8.2% |
| Quite disagree | 631 (94.9) | 34 (5.1) |  | 8.9% | 8.2% |
| Neither disagree nor agree | 1146 (92.8) | 89 (7.2) |  | 16.1% | 21.4% |
| Agree enough | 1387 (95.4) | 67 (4.6) |  | 19.5% | 16.1% |
| Agree | 1248 (97.3) | 35 (2.7) |  | 17.6% | 8.4% |
| Strongly agree | 1651 (98.2) | 30 (1.8) |  | 23.3% | 7.2% |
| **Ban on mass gatherings in streets** | | | | | |
| Strongly disagree | 120 (64.2) | 67 (35.8) | <0.001 | 1.7% | 16.1% |
| Disagree | 106 (86.2) | 17 (13.8) |  | 1.5% | 4.1% |
| Quite disagree | 180 (86.5) | 28 (13.5) |  | 2.5% | 6.7% |
| Neither disagree nor agree | 643 (87.8) | 89 (12.2) |  | 9.1% | 21.4% |
| Agree enough | 1217 (93.9) | 79 (6.1) |  | 17.1% | 19.0% |
| Agree | 1522 (96.0) | 64 (4.0) |  | 21.4% | 15.4% |
| Strongly agree | 3309 (97.9) | 72 (2.1) |  | 46.6% | 17.3% |
| **Introduction of mandatory testing of school teachers** | | | | | |
| Strongly disagree | 123 (66.8) | 61 (33.2) | <0.001 | 1.7% | 14.7% |
| Disagree | 93 (82.3) | 20 (17.7) |  | 1.3% | 4.8% |
| Quite disagree | 153 (82.7) | 32 (17.3) |  | 2.2% | 7.7% |
| Neither disagree nor agree | 896 (89.4) | 106 (10.6) |  | 12.6% | 25.5% |
| Agree enough | 1547 (95.1) | 79 (4.9) |  | 21.8% | 19.0% |
| Agree | 1693 (96.2) | 66 (3.8) |  | 23.9% | 15.9% |
| Strongly agree | 2592 (98.0) | 52 (2.0) |  | 36.5% | 12.5% |
| **Opening borders to more countries** | | | | | |
| Strongly disagree | 170 (75.9) | 54 (24.1) | <0.001 | 2.4% | 13.0% |
| Disagree | 169 (86.2) | 27 (13.8) |  | 2.4% | 6.5% |
| Quite disagree | 267 (88.4) | 35 (11.6) |  | 3.8% | 8.4% |
| Neither disagree nor agree | 1131 (92.9) | 87 (7.1) |  | 15.9% | 20.9% |
| Agree enough | 1543 (95.7) | 69 (4.3) |  | 21.7% | 16.6% |
| Agree | 1350 (95.5) | 63 (4.5) |  | 19.0% | 15.1% |
| Strongly agree | 2467 (96.8) | 81 (3.2) |  | 34.8% | 19.5% |
| ***Unwanted behaviour*** | | | | | |
| **During the past 2 weeks, you made the following:** | | | | | |
| **Avoided people that I thought might infect me, based on their ethnicity** | | | | | |
| Yes | 897 (93.5) | 62 (6.5) | 0.015 | 12.6% | 14.9% |
| No | 5134 (94.9) | 274 (5.1) |  | 72.3% | 65.9% |
| Not applicable | 1066 (93.0) | 80 (7.0) |  | 15.0% | 19.2% |
| **Exercised less than I did before the pandemic** | | | | | |
| Yes | 3097 (95.4) | 149 (4.6) | <0.001 | 43.6% | 35.8% |
| No | 3042 (94.3) | 185 (5.7) |  | 42.9% | 44.5% |
| Not applicable | 958 (92.1) | 82 (7.9) |  | 13.5% | 19.7% |
| **Drank more alcohol than I did before the pandemic** | | | | | |
| Yes | 666 (93.0) | 50 (7.0) | <0.001 | 9.4% | 12.0% |
| No | 5408 (95.2) | 275 (4.8) |  | 76.2% | 66.1% |
| Not applicable | 1023 (91.8) | 91 (8.2) |  | 14.4% | 21.9% |
| **Ate more unhealthy food than I did before the pandemic** | | | | | |
| Yes | 1990 (94.8) | 110 (5.2) | <0.001 | 28.0% | 26.4% |
| No | 4637 (95.0) | 245 (5.0) |  | 65.3% | 58.9% |
| Not applicable | 470 (88.5) | 61 (11.5) |  | 6.6% | 14.7% |
| **Smoked more than I did before the pandemic** | | | | | |
| Yes | 826 (94.0) | 53 (6.0) | 0.739 | 11.6% | 12.7% |
| No | 3691 (94.6) | 210 (5.4) |  | 52.0% | 50.5% |
| Not applicable | 2580 (94.4) | 153 (5.6) |  | 36.4% | 36.8% |
| **Postponed vaccination for myself or my child** | | | | | |
| Yes | 437 (94.4) | 26 (5.6) | 0.032 | 6.2% | 6.2% |
| No | 4687 (94.9) | 250 (5.1) |  | 66.0% | 60.1% |
| Not applicable | 1973 (93.4) | 140 (6.6) |  | 27.8% | 33.7% |
| **Avoided going to the doctor for a non-COVID-19-related problem** | | | | | |
| Yes | 2358 (95.5) | 110 (4.5) | <0.001 | 33.2% | 26.4% |
| No | 3919 (94.6) | 224 (5.4) |  | 55.2% | 53.8% |
| Not applicable | 820 (90.9) | 82 (9.1) |  | 11.6% | 19.7% |
| **Bought drugs that I heard are good for treating COVID-19** | | | | | |
| Yes | 422 (91.7) | 38 (8.3) | <0.001 | 5.9% | 9.1% |
| No | 6018 (95.0) | 315 (5.0) |  | 84.8% | 75.7% |
| Not applicable | 657 (91.2) | 63 (8.8) |  | 9.3% | 15.1% |
| ***Feeling of wellbeing*** | | | | | |
| **Please indicate your general well-being during the past 2 weeks:** | | | | | |
| **I have felt cheerful and in good spirits** | | | | | |
| All time | 237 (89.8) | 27 (10.2) | <0.001 | 3.3% | 6.5% |
| Most of the time | 996 (96.0) | 42 (4.0) |  | 14.0% | 10.1% |
| More than half the time | 1819 (95.9) | 78 (4.1) |  | 25.6% | 18.8% |
| Less than half the time | 1690 (93.2) | 124 (6.8) |  | 23.8% | 29.8% |
| Some time | 1846 (94.5) | 107 (5.5) |  | 26.0% | 25.7% |
| In no time | 509 (93.1) | 38 (6.9) |  | 7.2% | 9.1% |
| **I have felt calm and relaxed** | | | | | |
| All time | 278 (90.0) | 31 (10.0) | 0.012 | 3.9% | 7.5% |
| Most of the time | 1106 (95.2) | 56 (4.8) |  | 15.6% | 13.5% |
| More than half the time | 1732 (94.2) | 106 (5.8) |  | 24.4% | 25.5% |
| Less than half the time | 1643 (94.6) | 94 (5.4) |  | 23.2% | 22.6% |
| Some time | 1703 (95.0) | 89 (5.0) |  | 24.0% | 21.4% |
| In no time | 635 (94.1) | 40 (5.9) |  | 8.9% | 9.6% |
| **I have felt active and vigorous** | | | | | |
| All time | 281 (89.5) | 33 (10.5) | 0.004 | 4.0% | 7.9% |
| Most of the time | 1052 (95.0) | 55 (5.0) |  | 14.8% | 13.2% |
| More than half the time | 1702 (94.9) | 92 (5.1) |  | 24.0% | 22.1% |
| Less than half the time | 1732 (94.1) | 109 (5.9) |  | 24.4% | 26.2% |
| Some time | 1673 (94.9) | 89 (5.1) |  | 23.6% | 21.4% |
| In no time | 657 (94.5) | 38 (5.5) |  | 9.3% | 9.1% |
| **I woke up feeling fresh and rested** | | | | | |
| All time | 279 (91.2) | 27 (8.8) | 0.003 | 3.9% | 6.5% |
| Most of the time | 1140 (95.8) | 50 (4.2) |  | 16.1% | 12.0% |
| More than half the time | 1492 (94.5) | 87 (5.5) |  | 21.0% | 20.9% |
| Less than half the time | 1565 (93.2) | 115 (6.8) |  | 22.1% | 27.6% |
| Some time | 1800 (95.3) | 89 (4.7) |  | 25.4% | 21.4% |
| In no time | 821 (94.5) | 48 (5.5) |  | 11.6% | 11.5% |
| **My daily life has been filled with things that interest me** | | | | | |
| All time | 339 (91.1) | 33 (8.9) | 0.032 | 4.8% | 7.9% |
| Most of the time | 1110 (95.5) | 52 (4.5) |  | 15.6% | 12.5% |
| More than half the time | 1790 (94.7) | 100 (5.3) |  | 25.2% | 24.0% |
| Less than half the time | 1656 (94.2) | 102 (5.8) |  | 23.3% | 24.5% |
| Some time | 1718 (94.8) | 95 (5.2) |  | 24.2% | 22.8% |
| In no time | 484 (93.4) | 34 (6.6) |  | 6.8% | 8.2% |
| ***COVID-19 vaccines*** | | | | | |
| **I believe a vaccine can help control the spread of COVID-19** | | | | | |
| Strongly disagree | 184 (73.9) | 65 (26.1) | <0.001 | 2.6% | 15.6% |
| Disagree | 158 (86.3) | 25 (13.7) |  | 2.2% | 6.0% |
| Quite disagree | 215 (89.2) | 26 (10.8) |  | 3.0% | 6.2% |
| Neither disagree nor agree | 1011 (90.1) | 111 (9.9) |  | 14.2% | 26.7% |
| Agree enough | 1338 (94.6) | 76 (5.4) |  | 18.9% | 18.3% |
| Agree | 1320 (96.0) | 55 (4.0) |  | 18.6% | 13.2% |
| Strongly agree | 2871 (98.0) | 58 (2.0) |  | 40.5% | 13.9% |
| **Apart from COVID-19, I think everyone should be vaccinated according to the national vaccination schedule** | | | | | |
| Yes | 5347 (97.0) | 167 (3.0) | <0.001 | 75.3% | 40.1% |
| No | 668 (80.4) | 163 (19.6) |  | 9.4% | 39.2% |
| Do not know | 1082 (92.6) | 86 (7.4) |  | 15.2% | 20.7% |

Fisher exact test or χ^2^-test was used to estimate the difference in contingency tables.

### S2 Table. Results of univariable generalized linear model analysis with forward selection in the training data set (n=5259).

Only variables that demonstrated between-group statistical difference at p<0.2 in the initial analysis are included (see “Methods” of the main manuscript for details).

| **Survey parameter** | **Willingness to share contacts in case of positivity** | | **OR (95% CI)** |
| --- | --- | --- | --- |
|  | Would share | Would not share |  |
| **Age, years** | 45.8 (12.9) | 43.8 (13.1) | 0.99 (0.98-1.00, p=0.015) |
| **Age group** | | | |
| 18-34 years | 1302 (93.8) | 86 (6.2) | Reference |
| 35-44 years | 951 (94.9) | 51 (5.1) | 0.81 (0.57-1.15, p=0.252) |
| 45-54 years | 1149 (94.4) | 68 (5.6) | 0.90 (0.64-1.24, p=0.511) |
| 55-70 years | 1579 (95.6) | 73 (4.4) | 0.70 (0.51-0.96, p=0.029) |
| **Do you have a chronic illness?** | | | |
| Yes | 1149 (95.8) | 50 (4.2) | Reference |
| No | 3652 (94.5) | 211 (5.5) | 1.33 (0.98-1.84, p=0.078) |
| Do not know | 180 (91.4) | 17 (8.6) | 2.17 (1.19-3.77, p=0.008) |
| **Please assess your private financial situation over the past three months:** | | | |
| Improved | 275 (94.5) | 16 (5.5) | Reference |
| Stayed the same | 2973 (95.3) | 148 (4.7) | 0.86 (0.52-1.51, p=0.564) |
| Worsened | 1685 (94.1) | 105 (5.9) | 1.07 (0.64-1.91, p=0.804) |
| Do not know | 48 (84.2) | 9 (15.8) | 3.22 (1.30-7.58, p=0.009) |
| **To your knowledge, are you, or have you been, infected with COVID-19?** | | | |
| Yes | 430 (95.8) | 19 (4.2) | Reference |
| No | 4156 (95.0) | 219 (5.0) | 1.19 (0.76-1.99, p=0.471) |
| Do not know | 395 (90.8) | 40 (9.2) | 2.29 (1.32-4.11, p=0.004) |
| **Do you know people in your immediate social environment who are or have been infected with COVID-19 (suspected or confirmed)?** | | | |
| Yes | 3678 (95.9) | 158 (4.1) | Reference |
| No | 1303 (91.6) | 120 (8.4) | 2.14 (1.67-2.74, p<0.001) |
| **How easy or hard it was to:** | | | |
| **…find the information you need related to COVID-19?** | | | |
| Neither difficult nor easy | 900 (91.8) | 80 (8.2) | Reference |
| Very difficult | 120 (93.8) | 8 (6.2) | 0.75 (0.33-1.50, p=0.453) |
| Hard | 100 (92.6) | 8 (7.4) | 0.90 (0.39-1.81, p=0.785) |
| Pretty hard | 343 (93.0) | 26 (7.0) | 0.85 (0.53-1.33, p=0.497) |
| Quite easy | 1665 (96.0) | 70 (4.0) | 0.47 (0.34-0.66, p<0.001) |
| Easy | 1033 (95.3) | 51 (4.7) | 0.56 (0.38-0.80, p=0.001) |
| Very easy | 820 (95.9) | 35 (4.1) | 0.48 (0.32-0.72, p<0.001) |
| **…understand information about what to do if you think you have COVID-19?** | | | |
| Neither difficult nor easy | 933 (93.7) | 63 (6.3) | Reference |
| Very difficult | 87 (90.6) | 9 (9.4) | 1.53 (0.69-3.04, p=0.253) |
| Hard | 230 (94.3) | 14 (5.7) | 0.90 (0.48-1.59, p=0.733) |
| Pretty hard | 592 (93.1) | 44 (6.9) | 1.10 (0.74-1.63, p=0.637) |
| Quite easy | 1648 (95.1) | 85 (4.9) | 0.76 (0.55-1.07, p=0.116) |
| Easy | 967 (96.4) | 36 (3.6) | 0.55 (0.36-0.83, p=0.005) |
| Very easy | 524 (95.1) | 27 (4.9) | 0.76 (0.47-1.20, p=0.253) |
| **…judge if the information about COVID-19 in the media is reliable?** | | | |
| Neither difficult nor easy | 1298 (95.2) | 66 (4.8) | Reference |
| Very difficult | 463 (91.3) | 44 (8.7) | 1.87 (1.25-2.77, p=0.002) |
| Hard | 396 (94.3) | 24 (5.7) | 1.19 (0.72-1.90, p=0.474) |
| Pretty hard | 1031 (94.8) | 56 (5.2) | 1.07 (0.74-1.54, p=0.723) |
| Quite easy | 1053 (96.0) | 44 (4.0) | 0.82 (0.55-1.21, p=0.324) |
| Easy | 510 (95.0) | 27 (5.0) | 1.04 (0.65-1.63, p=0.863) |
| Very easy | 230 (93.1) | 17 (6.9) | 1.45 (0.81-2.47, p=0.183) |
| **…understand restrictions and recommendations of authorities regarding COVID-19?** | | | |
| Neither difficult nor easy | 864 (93.6) | 59 (6.4) | Reference |
| Very difficult | 182 (82.4) | 39 (17.6) | 3.14 (2.02-4.83, p<0.001) |
| Hard | 309 (91.7) | 28 (8.3) | 1.33 (0.82-2.10, p=0.236) |
| Pretty hard | 637 (94.4) | 38 (5.6) | 0.87 (0.57-1.32, p=0.529) |
| Quite easy | 1427 (95.8) | 62 (4.2) | 0.64 (0.44-0.92, p=0.016) |
| Easy | 954 (96.5) | 35 (3.5) | 0.54 (0.35-0.82, p=0.004) |
| Very easy | 608 (97.3) | 17 (2.7) | 0.41 (0.23-0.69, p=0.001) |
| **…follow the recommendations on how to protect yourself from COVID-19?** | | | |
| Neither difficult nor easy | 674 (92.0) | 59 (8.0) | Reference |
| Very difficult | 111 (82.2) | 24 (17.8) | 2.47 (1.45-4.09, p=0.001) |
| Hard | 77 (78.6) | 21 (21.4) | 3.12 (1.76-5.34, p<0.001) |
| Pretty hard | 248 (94.7) | 14 (5.3) | 0.64 (0.34-1.14, p=0.152) |
| Quite easy | 1635 (95.7) | 74 (4.3) | 0.52 (0.36-0.74, p<0.001) |
| Easy | 1263 (95.8) | 56 (4.2) | 0.51 (0.35-0.74, p<0.001) |
| Very easy | 973 (97.0) | 30 (3.0) | 0.35 (0.22-0.55, p<0.001) |
| **…understand recommendations about when to stay at home from work/school, and when not to?** | | | |
| Neither difficult nor easy | 844 (93.6) | 58 (6.4) | Reference |
| Very difficult | 90 (71.4) | 36 (28.6) | 5.82 (3.62-9.28, p<0.001) |
| Hard | 133 (89.3) | 16 (10.7) | 1.75 (0.95-3.07, p=0.060) |
| Pretty hard | 496 (93.6) | 34 (6.4) | 1.00 (0.64-1.54, p=0.991) |
| Quite easy | 1579 (96.3) | 61 (3.7) | 0.56 (0.39-0.81, p=0.002) |
| Easy | 1148 (95.4) | 55 (4.6) | 0.70 (0.48-1.02, p=0.062) |
| Very easy | 691 (97.5) | 18 (2.5) | 0.38 (0.22-0.64, p<0.001) |
| **…follow recommendations about when to stay at home from work/school, and when not to?** | | | |
| Neither difficult nor easy | 804 (92.4) | 66 (7.6) | Reference |
| Very difficult | 87 (73.1) | 32 (26.9) | 4.48 (2.76-7.18, p<0.001) |
| Hard | 170 (89.0) | 21 (11.0) | 1.50 (0.88-2.49, p=0.122) |
| Pretty hard | 420 (91.7) | 38 (8.3) | 1.10 (0.72-1.66, p=0.647) |
| Quite easy | 1628 (96.4) | 61 (3.6) | 0.46 (0.32-0.65, p<0.001) |
| Easy | 1137 (96.3) | 44 (3.7) | 0.47 (0.32-0.70, p<0.001) |
| Very easy | 735 (97.9) | 16 (2.1) | 0.27 (0.15-0.45, p<0.001) |
| **…understand recommendations about when to engage in social activities, and when not to?** | | | |
| Neither difficult nor easy | 843 (93.9) | 55 (6.1) | Reference |
| Very difficult | 89 (74.2) | 31 (25.8) | 5.34 (3.24-8.69, p<0.001) |
| Hard | 120 (87.6) | 17 (12.4) | 2.17 (1.19-3.79, p=0.008) |
| Pretty hard | 403 (90.8) | 41 (9.2) | 1.56 (1.02-2.37, p=0.039) |
| Quite easy | 1644 (95.7) | 73 (4.3) | 0.68 (0.48-0.98, p=0.036) |
| Easy | 1189 (96.5) | 43 (3.5) | 0.55 (0.37-0.83, p=0.005) |
| Very easy | 693 (97.5) | 18 (2.5) | 0.40 (0.23-0.67, p=0.001) |
| **…follow recommendations about when to engage in social activities, and when not to?** | | | |
| Neither difficult nor easy | 847 (93.6) | 58 (6.4) | Reference |
| Very difficult | 76 (74.5) | 26 (25.5) | 5.00 (2.94-8.33, p<0.001) |
| Hard | 113 (81.9) | 25 (18.1) | 3.23 (1.92-5.32, p<0.001) |
| Pretty hard | 414 (90.0) | 46 (10.0) | 1.62 (1.08-2.43, p=0.019) |
| Quite easy | 1597 (96.4) | 60 (3.6) | 0.55 (0.38-0.80, p=0.001) |
| Easy | 1223 (96.5) | 44 (3.5) | 0.53 (0.35-0.78, p=0.002) |
| Very easy | 711 (97.4) | 19 (2.6) | 0.39 (0.22-0.65, p<0.001) |
| **How do you estimate your own probability of getting COVID-19 infection?** | | | |
| Neither improbable nor probable | 2717 (95.1) | 139 (4.9) | Reference |
| Very unlikely | 237 (88.8) | 30 (11.2) | 2.47 (1.61-3.70, p<0.001) |
| Unlikely | 207 (86.6) | 32 (13.4) | 3.02 (1.98-4.50, p<0.001) |
| Quite unlikely | 667 (95.0) | 35 (5.0) | 1.03 (0.69-1.48, p=0.896) |
| Quite likely | 772 (97.1) | 23 (2.9) | 0.58 (0.36-0.89, p=0.018) |
| Likely | 235 (94.8) | 13 (5.2) | 1.08 (0.58-1.87, p=0.793) |
| Very likely | 146 (96.1) | 6 (3.9) | 0.80 (0.31-1.70, p=0.607) |
| **How susceptible do you consider yourself to an infection with COVID-19?** | | | |
| Neither vulnerable nor invulnerable | 2721 (95.1) | 139 (4.9) | Reference |
| Totally vulnerable | 161 (98.2) | 3 (1.8) | 0.36 (0.09-0.98, p=0.087) |
| vulnerable | 285 (95.6) | 13 (4.4) | 0.89 (0.48-1.54, p=0.703) |
| Quite vulnerable | 898 (97.2) | 26 (2.8) | 0.57 (0.36-0.85, p=0.009) |
| Quite invulnerable | 694 (91.8) | 62 (8.2) | 1.75 (1.27-2.37, p<0.001) |
| Invulnerable | 112 (86.8) | 17 (13.2) | 2.97 (1.68-4.96, p<0.001) |
| Totally invulnerable | 110 (85.9) | 18 (14.1) | 3.20 (1.84-5.30, p<0.001) |
| **How severe would contracting COVID-19 be for you (how seriously ill do you think you will be)?** | | | |
| Neither serious nor serious | 1516 (93.8) | 101 (6.2) | Reference |
| Not at all serious | 137 (83.0) | 28 (17.0) | 3.07 (1.92-4.77, p<0.001) |
| Little serious | 277 (93.3) | 20 (6.7) | 1.08 (0.64-1.74, p=0.751) |
| Not particularly bad | 802 (94.0) | 51 (6.0) | 0.95 (0.67-1.34, p=0.793) |
| Serious enough | 1500 (96.5) | 55 (3.5) | 0.55 (0.39-0.77, p<0.001) |
| Serious | 391 (97.8) | 9 (2.2) | 0.35 (0.16-0.65, p=0.003) |
| Very serious | 358 (96.2) | 14 (3.8) | 0.59 (0.32-1.00, p=0.067) |
| **I know how to protect myself from coronavirus** | | | |
| Neither disagree nor agree | 733 (92.6) | 59 (7.4) | Reference |
| Strongly disagree | 74 (93.7) | 5 (6.3) | 0.84 (0.29-1.97, p=0.716) |
| Disagree | 33 (82.5) | 7 (17.5) | 2.64 (1.03-5.89, p=0.027) |
| Quite disagree | 129 (92.1) | 11 (7.9) | 1.06 (0.52-1.99, p=0.866) |
| Agree enough | 2131 (95.3) | 106 (4.7) | 0.62 (0.45-0.86, p=0.004) |
| Agree | 1160 (96.0) | 48 (4.0) | 0.51 (0.35-0.76, p=0.001) |
| Strongly agree | 721 (94.5) | 42 (5.5) | 0.72 (0.48-1.09, p=0.121) |
| **For me avoiding an infection with COVID-19 in the current situation is…** | | | |
| Neither difficult nor easy | 2429 (95.1) | 126 (4.9) | Reference |
| Very difficult | 83 (89.2) | 10 (10.8) | 2.32 (1.11-4.38, p=0.015) |
| Hard | 103 (96.3) | 4 (3.7) | 0.75 (0.23-1.82, p=0.576) |
| Pretty hard | 446 (95.1) | 23 (4.9) | 0.99 (0.62-1.54, p=0.980) |
| Quite easy | 1381 (94.7) | 77 (5.3) | 1.07 (0.80-1.43, p=0.627) |
| Easy | 351 (92.4) | 29 (7.6) | 1.59 (1.03-2.39, p=0.029) |
| Very easy | 188 (95.4) | 9 (4.6) | 0.92 (0.43-1.74, p=0.820) |
| **During the past 7 days, which measures you have performed to prevent to be infected by COVID-19?** | | | |
| **Frequently washed my hands with soap and water for at least 20 seconds** | | | |
| Some time | 353 (89.1) | 43 (10.9) | Reference |
| Never | 23 (53.5) | 20 (46.5) | 7.14 (3.61-14.10, p<0.001) |
| Hardly ever | 61 (81.3) | 14 (18.7) | 1.88 (0.94-3.58, p=0.061) |
| Very little | 86 (85.1) | 15 (14.9) | 1.43 (0.74-2.65, p=0.267) |
| Often enough | 863 (93.1) | 64 (6.9) | 0.61 (0.41-0.92, p=0.017) |
| Often | 1241 (96.3) | 48 (3.7) | 0.32 (0.21-0.49, p<0.001) |
| Very often | 2354 (97.0) | 74 (3.0) | 0.26 (0.18-0.38, p<0.001) |
| **Avoided touching my eyes, nose and mouth with unwashed hands** | | | |
| Some time | 596 (91.8) | 53 (8.2) | Reference |
| Never | 57 (66.3) | 29 (33.7) | 5.72 (3.35-9.67, p<0.001) |
| Hardly ever | 77 (86.5) | 12 (13.5) | 1.75 (0.86-3.32, p=0.101) |
| Very little | 163 (87.2) | 24 (12.8) | 1.66 (0.98-2.73, p=0.054) |
| Often enough | 1046 (94.7) | 59 (5.3) | 0.63 (0.43-0.93, p=0.020) |
| Often | 1110 (96.3) | 43 (3.7) | 0.44 (0.29-0.66, p<0.001) |
| Very often | 1932 (97.1) | 58 (2.9) | 0.34 (0.23-0.50, p<0.001) |
| **Used disinfectants to clean hands when soap and water were not available** | | | |
| Some time | 362 (87.9) | 50 (12.1) | Reference |
| Never | 52 (63.4) | 30 (36.6) | 4.18 (2.43-7.14, p<0.001) |
| Hardly ever | 47 (75.8) | 15 (24.2) | 2.31 (1.17-4.36, p=0.012) |
| Very little | 74 (85.1) | 13 (14.9) | 1.27 (0.63-2.40, p=0.475) |
| Often enough | 663 (93.6) | 45 (6.4) | 0.49 (0.32-0.75, p=0.001) |
| Often | 1104 (96.0) | 46 (4.0) | 0.30 (0.20-0.46, p<0.001) |
| Very often | 2679 (97.1) | 79 (2.9) | 0.21 (0.15-0.31, p<0.001) |
| **Avoided a social event I wanted to attend** | | | |
| Some time | 401 (92.4) | 33 (7.6) | Reference |
| Never | 191 (82.7) | 40 (17.3) | 2.54 (1.56-4.18, p<0.001) |
| Hardly ever | 97 (88.2) | 13 (11.8) | 1.63 (0.80-3.14, p=0.159) |
| Very little | 112 (89.6) | 13 (10.4) | 1.41 (0.70-2.71, p=0.318) |
| Often enough | 612 (93.2) | 45 (6.8) | 0.89 (0.56-1.43, p=0.636) |
| Often | 928 (94.9) | 50 (5.1) | 0.65 (0.42-1.04, p=0.068) |
| Very often | 2640 (96.9) | 84 (3.1) | 0.39 (0.26-0.59, p<0.001) |
| **Used medicines to prevent or treat COVID-19** | | | |
| Some time | 247 (88.5) | 32 (11.5) | Reference |
| Never | 3632 (95.4) | 174 (4.6) | 0.37 (0.25-0.56, p<0.001) |
| Hardly ever | 359 (97.0) | 11 (3.0) | 0.24 (0.11-0.46, p<0.001) |
| Very little | 271 (92.2) | 23 (7.8) | 0.66 (0.37-1.15, p=0.141) |
| Often enough | 163 (89.1) | 20 (10.9) | 0.95 (0.52-1.70, p=0.857) |
| Often | 139 (94.6) | 8 (5.4) | 0.44 (0.19-0.95, p=0.047) |
| Very often | 170 (94.4) | 10 (5.6) | 0.45 (0.21-0.92, p=0.036) |
| **Wore a mask in public** | | | |
| Some time | 83 (78.3) | 23 (21.7) | Reference |
| Never | 19 (73.1) | 7 (26.9) | 1.33 (0.47-3.45, p=0.570) |
| Hardly ever | 13 (61.9) | 8 (38.1) | 2.22 (0.80-5.95, p=0.116) |
| Very little | 33 (84.6) | 6 (15.4) | 0.66 (0.23-1.67, p=0.402) |
| Often enough | 175 (86.2) | 28 (13.8) | 0.58 (0.31-1.07, p=0.078) |
| Often | 358 (90.6) | 37 (9.4) | 0.37 (0.21-0.67, p=0.001) |
| Very often | 4300 (96.2) | 169 (3.8) | 0.14 (0.09-0.24, p<0.001) |
| **Ensured physical distancing in public** | | | |
| Some time | 167 (83.1) | 34 (16.9) | Reference |
| Never | 16 (55.2) | 13 (44.8) | 3.99 (1.74-9.07, p=0.001) |
| Hardly ever | 18 (66.7) | 9 (33.3) | 2.46 (0.98-5.82, p=0.046) |
| Very little | 42 (76.4) | 13 (23.6) | 1.52 (0.72-3.08, p=0.256) |
| Often enough | 599 (91.3) | 57 (8.7) | 0.47 (0.30-0.74, p=0.001) |
| Often | 1124 (95.4) | 54 (4.6) | 0.24 (0.15-0.38, p<0.001) |
| Very often | 3015 (96.9) | 98 (3.1) | 0.16 (0.11-0.25, p<0.001) |
| **Disinfected surfaces** | | | |
| Some time | 761 (93.4) | 54 (6.6) | Reference |
| Never | 94 (66.7) | 47 (33.3) | 7.05 (4.51-11.02, p<0.001) |
| Hardly ever | 123 (90.4) | 13 (9.6) | 1.49 (0.76-2.73, p=0.219) |
| Very little | 211 (92.1) | 18 (7.9) | 1.20 (0.67-2.05, p=0.515) |
| Often enough | 1069 (95.7) | 48 (4.3) | 0.63 (0.42-0.94, p=0.025) |
| Often | 1125 (95.5) | 53 (4.5) | 0.66 (0.45-0.98, p=0.040) |
| Very often | 1598 (97.3) | 45 (2.7) | 0.40 (0.26-0.59, p<0.001) |
| **Please select the choice that is better express your opinion about the COVID-19** | | | |
| **How close or far away it from you** | | | |
| Close to me | 772 (97.7) | 18 (2.3) | 0.46 (0.26-0.75, p=0.003) |
| 2 | 729 (97.1) | 22 (2.9) | 0.59 (0.36-0.94, p=0.031) |
| 3 | 761 (95.5) | 36 (4.5) | 0.92 (0.61-1.37, p=0.699) |
| 4 | 1562 (95.1) | 80 (4.9) | Reference |
| 5 | 448 (92.0) | 39 (8.0) | 1.70 (1.13-2.51, p=0.009) |
| 6 | 296 (90.0) | 33 (10.0) | 2.18 (1.41-3.30, p<0.001) |
| Away from me | 413 (89.2) | 50 (10.8) | 2.36 (1.63-3.41, p<0.001) |
| **Whether it is spreading slowly or fast** | | | |
| Slowly growing | 430 (90.5) | 45 (9.5) | 1.51 (1.03-2.20, p=0.032) |
| 2 | 405 (93.8) | 27 (6.2) | 0.96 (0.61-1.49, p=0.871) |
| 3 | 545 (94.0) | 35 (6.0) | 0.93 (0.61-1.38, p=0.720) |
| 4 | 1214 (93.5) | 84 (6.5) | Reference |
| 5 | 771 (94.6) | 44 (5.4) | 0.82 (0.56-1.19, p=0.315) |
| 6 | 654 (96.3) | 25 (3.7) | 0.55 (0.34-0.86, p=0.011) |
| Fast growing | 962 (98.2) | 18 (1.8) | 0.27 (0.16-0.44, p<0.001) |
| **How often you think about it** | | | |
| Something I always think about | 1170 (97.3) | 33 (2.7) | 0.47 (0.31-0.71, p<0.001) |
| 2 | 925 (97.8) | 21 (2.2) | 0.38 (0.23-0.61, p<0.001) |
| 3 | 827 (94.9) | 44 (5.1) | 0.89 (0.60-1.30, p=0.538) |
| 4 | 1199 (94.3) | 72 (5.7) | Reference |
| 5 | 429 (91.3) | 41 (8.7) | 1.59 (1.06-2.36, p=0.022) |
| 6 | 219 (88.7) | 28 (11.3) | 2.13 (1.33-3.34, p=0.001) |
| Something I never think about | 212 (84.5) | 39 (15.5) | 3.06 (2.01-4.62, p<0.001) |
| **How fear-inducing it is** | | | |
| Scary | 1635 (98.1) | 32 (1.9) | 0.25 (0.16-0.38, p<0.001) |
| 2 | 1014 (97.4) | 27 (2.6) | 0.34 (0.21-0.53, p<0.001) |
| 3 | 824 (94.5) | 48 (5.5) | 0.74 (0.51-1.09, p=0.130) |
| 4 | 869 (92.7) | 68 (7.3) | Reference |
| 5 | 282 (92.8) | 22 (7.2) | 1.00 (0.59-1.62, p=0.991) |
| 6 | 159 (85.9) | 26 (14.1) | 2.09 (1.27-3.35, p=0.003) |
| Not scary | 198 (78.3) | 55 (21.7) | 3.55 (2.40-5.23, p<0.001) |
| **How media infrom about it** | | | |
| Enlarged by the Media | 798 (88.6) | 103 (11.4) | 3.38 (2.39-4.85, p<0.001) |
| 2 | 455 (94.6) | 26 (5.4) | 1.50 (0.91-2.42, p=0.106) |
| 3 | 545 (93.3) | 39 (6.7) | 1.87 (1.21-2.89, p=0.005) |
| 4 | 1257 (96.3) | 48 (3.7) | Reference |
| 5 | 498 (94.9) | 27 (5.1) | 1.42 (0.87-2.28, p=0.155) |
| 6 | 552 (96.3) | 21 (3.7) | 1.00 (0.58-1.66, p=0.989) |
| Not exaggerated by the media | 876 (98.4) | 14 (1.6) | 0.42 (0.22-0.74, p=0.005) |
| **What is your perception about it** | | | |
| Something that makes me feel helpless | 1288 (97.1) | 39 (2.9) | 0.42 (0.28-0.64, p<0.001) |
| 2 | 705 (96.3) | 27 (3.7) | 0.54 (0.33-0.84, p=0.009) |
| 3 | 584 (94.5) | 34 (5.5) | 0.82 (0.53-1.25, p=0.358) |
| 4 | 870 (93.3) | 62 (6.7) | Reference |
| 5 | 531 (94.5) | 31 (5.5) | 0.82 (0.52-1.27, p=0.379) |
| 6 | 479 (94.9) | 26 (5.1) | 0.76 (0.47-1.21, p=0.258) |
| Something that I am able to cope with with my behaviors | 524 (89.9) | 59 (10.1) | 1.58 (1.09-2.29, p=0.016) |
| **How stressful it is** | | | |
| Stressful | 2579 (95.2) | 129 (4.8) | 0.60 (0.42-0.88, p=0.007) |
| 2 | 960 (96.4) | 36 (3.6) | 0.45 (0.28-0.72, p=0.001) |
| 3 | 539 (96.1) | 22 (3.9) | 0.49 (0.28-0.83, p=0.009) |
| 4 | 483 (92.4) | 40 (7.6) | Reference |
| 5 | 199 (90.0) | 22 (10.0) | 1.33 (0.76-2.28, p=0.300) |
| 6 | 82 (89.1) | 10 (10.9) | 1.47 (0.67-2.95, p=0.300) |
| Not stressful | 139 (88.0) | 19 (12.0) | 1.65 (0.91-2.90, p=0.089) |
| **How much you trust to the information about the COVID-19 supplied by the following sources:** | | | |
| **Television** | | | |
| Neither do I trust nor do I trust | 1533 (95.9) | 65 (4.1) | Reference |
| Do not trust at all | 469 (85.9) | 77 (14.1) | 3.87 (2.74-5.48, p<0.001) |
| Trust very little | 470 (93.4) | 33 (6.6) | 1.66 (1.06-2.53, p=0.022) |
| Trust little | 770 (94.8) | 42 (5.2) | 1.29 (0.86-1.91, p=0.214) |
| Trust enough | 1299 (96.9) | 42 (3.1) | 0.76 (0.51-1.13, p=0.179) |
| Trust a lot | 332 (96.2) | 13 (3.8) | 0.92 (0.48-1.64, p=0.797) |
| Trust very much | 108 (94.7) | 6 (5.3) | 1.31 (0.50-2.86, p=0.537) |
| **Newspapers** | | | |
| Neither do I trust nor do I trust | 1636 (95.1) | 85 (4.9) | Reference |
| Do not trust at all | 467 (87.8) | 65 (12.2) | 2.68 (1.90-3.75, p<0.001) |
| Trust very little | 427 (92.4) | 35 (7.6) | 1.58 (1.04-2.35, p=0.028) |
| Trust little | 746 (95.2) | 38 (4.8) | 0.98 (0.66-1.44, p=0.921) |
| Trust enough | 1312 (97.3) | 36 (2.7) | 0.53 (0.35-0.78, p=0.002) |
| Trust a lot | 302 (95.9) | 13 (4.1) | 0.83 (0.44-1.45, p=0.536) |
| Trust very much | 91 (93.8) | 6 (6.2) | 1.27 (0.48-2.75, p=0.585) |
| **Health workers** | | | |
| Neither do I trust nor do I trust | 667 (90.5) | 70 (9.5) | Reference |
| Do not trust at all | 74 (74.0) | 26 (26.0) | 3.35 (1.99-5.53, p<0.001) |
| Trust very little | 82 (83.7) | 16 (16.3) | 1.86 (1.00-3.28, p=0.039) |
| Trust little | 207 (84.1) | 39 (15.9) | 1.80 (1.17-2.72, p=0.007) |
| Trust enough | 1809 (96.2) | 71 (3.8) | 0.37 (0.27-0.53, p<0.001) |
| Trust a lot | 1422 (97.4) | 38 (2.6) | 0.25 (0.17-0.38, p<0.001) |
| Trust very much | 720 (97.6) | 18 (2.4) | 0.24 (0.14-0.40, p<0.001) |
| **Radio** | | | |
| Neither do I trust nor do I trust | 1832 (95.3) | 90 (4.7) | Reference |
| Do not trust at all | 323 (86.1) | 52 (13.9) | 3.28 (2.27-4.69, p<0.001) |
| Trust very little | 323 (93.4) | 23 (6.6) | 1.45 (0.88-2.29, p=0.124) |
| Trust little | 679 (93.3) | 49 (6.7) | 1.47 (1.02-2.09, p=0.036) |
| Trust enough | 1395 (96.9) | 44 (3.1) | 0.64 (0.44-0.92, p=0.018) |
| Trust a lot | 323 (95.6) | 15 (4.4) | 0.95 (0.52-1.60, p=0.844) |
| Trust very much | 106 (95.5) | 5 (4.5) | 0.96 (0.33-2.19, p=0.931) |
| **Ministry of Health** | | | |
| Neither do I trust nor do I trust | 795 (92.8) | 62 (7.2) | Reference |
| Do not trust at all | 187 (76.3) | 58 (23.7) | 3.98 (2.69-5.89, p<0.001) |
| Trust very little | 176 (87.1) | 26 (12.9) | 1.89 (1.15-3.05, p=0.010) |
| Trust little | 324 (91.8) | 29 (8.2) | 1.15 (0.72-1.80, p=0.557) |
| Trust enough | 1614 (96.6) | 56 (3.4) | 0.44 (0.31-0.64, p<0.001) |
| Trust a lot | 1235 (97.6) | 30 (2.4) | 0.31 (0.20-0.48, p<0.001) |
| Trust very much | 650 (97.5) | 17 (2.5) | 0.34 (0.19-0.57, p<0.001) |
| **Institute of Public Health/Center for Disease Control** | | | |
| Neither do I trust nor do I trust | 792 (93.2) | 58 (6.8) | Reference |
| Do not trust at all | 181 (77.0) | 54 (23.0) | 4.07 (2.72-6.11, p<0.001) |
| Trust very little | 151 (85.3) | 26 (14.7) | 2.35 (1.42-3.82, p=0.001) |
| Trust little | 340 (90.7) | 35 (9.3) | 1.41 (0.90-2.17, p=0.128) |
| Trust enough | 1639 (96.4) | 61 (3.6) | 0.51 (0.35-0.74, p<0.001) |
| Trust a lot | 1213 (97.7) | 28 (2.3) | 0.32 (0.20-0.49, p<0.001) |
| Trust very much | 665 (97.7) | 16 (2.3) | 0.33 (0.18-0.56, p<0.001) |
| **Celebrities and social media influencers** | | | |
| Neither do I trust nor do I trust | 1247 (95.6) | 58 (4.4) | Reference |
| Do not trust at all | 1465 (92.7) | 116 (7.3) | 1.70 (1.24-2.37, p=0.001) |
| Trust very little | 673 (95.9) | 29 (4.1) | 0.93 (0.58-1.45, p=0.742) |
| Trust little | 999 (95.8) | 44 (4.2) | 0.95 (0.63-1.41, p=0.790) |
| Trust enough | 413 (96.0) | 17 (4.0) | 0.88 (0.49-1.50, p=0.664) |
| Trust a lot | 115 (94.3) | 7 (5.7) | 1.31 (0.53-2.75, p=0.514) |
| Trust very much | 69 (90.8) | 7 (9.2) | 2.18 (0.88-4.66, p=0.063) |
| **World Health Organization (WHO)** | | | |
| Neither do I trust nor do I trust | 869 (94.3) | 53 (5.7) | Reference |
| Do not trust at all | 215 (77.9) | 61 (22.1) | 4.65 (3.13-6.94, p<0.001) |
| Trust very little | 166 (86.5) | 26 (13.5) | 2.57 (1.54-4.19, p<0.001) |
| Trust little | 407 (92.5) | 33 (7.5) | 1.33 (0.84-2.07, p=0.215) |
| Trust enough | 1560 (96.4) | 58 (3.6) | 0.61 (0.42-0.89, p=0.011) |
| Trust a lot | 1153 (97.4) | 31 (2.6) | 0.44 (0.28-0.69, p<0.001) |
| Trust very much | 611 (97.4) | 16 (2.6) | 0.43 (0.24-0.74, p=0.004) |
| **COVID-19 Hotlines** | | | |
| Neither do I trust nor do I trust | 1709 (95.3) | 85 (4.7) | Reference |
| Do not trust at all | 294 (84.2) | 55 (15.8) | 3.76 (2.61-5.38, p<0.001) |
| Trust very little | 234 (90.0) | 26 (10.0) | 2.23 (1.39-3.49, p=0.001) |
| Trust little | 504 (93.0) | 38 (7.0) | 1.52 (1.01-2.23, p=0.039) |
| Trust enough | 1389 (96.6) | 49 (3.4) | 0.71 (0.49-1.01, p=0.060) |
| Trust a lot | 612 (97.6) | 15 (2.4) | 0.49 (0.27-0.83, p=0.013) |
| Trust very much | 239 (96.0) | 10 (4.0) | 0.84 (0.41-1.57, p=0.613) |
| **National COVID-19 information website** | | | |
| Neither do I trust nor do I trust | 1140 (94.3) | 69 (5.7) | Reference |
| Do not trust at all | 177 (79.4) | 46 (20.6) | 4.29 (2.85-6.42, p<0.001) |
| Trust very little | 187 (85.4) | 32 (14.6) | 2.83 (1.79-4.39, p<0.001) |
| Trust little | 393 (90.8) | 40 (9.2) | 1.68 (1.11-2.51, p=0.012) |
| Trust enough | 1735 (97.0) | 53 (3.0) | 0.50 (0.35-0.73, p<0.001) |
| Trust a lot | 989 (97.6) | 24 (2.4) | 0.40 (0.25-0.63, p<0.001) |
| Trust very much | 360 (96.3) | 14 (3.7) | 0.64 (0.34-1.12, p=0.139) |
| **How often do you use COVID-19 related information from the following sources:** | | | |
| **Television** | | | |
| Some time | 1209 (94.2) | 75 (5.8) | Reference |
| Never | 399 (88.1) | 54 (11.9) | 2.18 (1.50-3.14, p<0.001) |
| Hardly ever | 343 (95.3) | 17 (4.7) | 0.80 (0.45-1.34, p=0.415) |
| Very little | 525 (94.6) | 30 (5.4) | 0.92 (0.59-1.41, p=0.712) |
| Often enough | 1306 (96.6) | 46 (3.4) | 0.57 (0.39-0.82, p=0.003) |
| Often | 661 (94.6) | 38 (5.4) | 0.93 (0.61-1.38, p=0.710) |
| Very often | 538 (96.8) | 18 (3.2) | 0.54 (0.31-0.89, p=0.021) |
| **Newspapers** | | | |
| Some time | 1383 (94.6) | 79 (5.4) | Reference |
| Never | 912 (91.4) | 86 (8.6) | 1.65 (1.20-2.27, p=0.002) |
| Hardly ever | 605 (95.1) | 31 (4.9) | 0.90 (0.58-1.36, p=0.617) |
| Very little | 744 (94.9) | 40 (5.1) | 0.94 (0.63-1.38, p=0.761) |
| Often enough | 817 (96.9) | 26 (3.1) | 0.56 (0.35-0.86, p=0.011) |
| Often | 336 (96.6) | 12 (3.4) | 0.63 (0.32-1.12, p=0.137) |
| Very often | 184 (97.9) | 4 (2.1) | 0.38 (0.12-0.93, p=0.062) |
| **Health workers** | | | |
| Some time | 1535 (95.4) | 74 (4.6) | Reference |
| Never | 482 (88.1) | 65 (11.9) | 2.80 (1.97-3.96, p<0.001) |
| Hardly ever | 419 (93.5) | 29 (6.5) | 1.44 (0.91-2.21, p=0.109) |
| Very little | 515 (93.5) | 36 (6.5) | 1.45 (0.95-2.17, p=0.076) |
| Often enough | 1063 (95.9) | 46 (4.1) | 0.90 (0.61-1.30, p=0.574) |
| Often | 637 (97.1) | 19 (2.9) | 0.62 (0.36-1.01, p=0.066) |
| Very often | 330 (97.3) | 9 (2.7) | 0.57 (0.26-1.08, p=0.112) |
| **Social media** | | | |
| Some time | 1112 (94.6) | 63 (5.4) | Reference |
| Never | 1350 (94.0) | 86 (6.0) | 1.12 (0.81-1.58, p=0.492) |
| Hardly ever | 735 (96.0) | 31 (4.0) | 0.74 (0.47-1.15, p=0.189) |
| Very little | 774 (95.8) | 34 (4.2) | 0.78 (0.50-1.18, p=0.243) |
| Often enough | 642 (94.8) | 35 (5.2) | 0.96 (0.62-1.46, p=0.859) |
| Often | 230 (91.6) | 21 (8.4) | 1.61 (0.94-2.65, p=0.069) |
| Very often | 138 (94.5) | 8 (5.5) | 1.02 (0.44-2.06, p=0.953) |
| **Radio** | | | |
| Some time | 1383 (95.8) | 60 (4.2) | Reference |
| Never | 955 (91.9) | 84 (8.1) | 2.03 (1.44-2.86, p<0.001) |
| Hardly ever | 690 (94.5) | 40 (5.5) | 1.34 (0.88-2.01, p=0.166) |
| Very little | 798 (95.2) | 40 (4.8) | 1.16 (0.76-1.73, p=0.489) |
| Often enough | 720 (95.7) | 32 (4.3) | 1.02 (0.65-1.58, p=0.914) |
| Often | 298 (95.2) | 15 (4.8) | 1.16 (0.63-2.02, p=0.615) |
| Very often | 137 (95.1) | 7 (4.9) | 1.18 (0.48-2.46, p=0.689) |
| **Ministry of Health** | | | |
| Some time | 1436 (94.9) | 77 (5.1) | Reference |
| Never | 517 (85.9) | 85 (14.1) | 3.07 (2.22-4.25, p<0.001) |
| Hardly ever | 341 (93.7) | 23 (6.3) | 1.26 (0.76-2.00, p=0.349) |
| Very little | 456 (92.7) | 36 (7.3) | 1.47 (0.97-2.20, p=0.064) |
| Often enough | 1113 (97.5) | 29 (2.5) | 0.49 (0.31-0.74, p=0.001) |
| Often | 728 (97.8) | 16 (2.2) | 0.41 (0.23-0.69, p=0.001) |
| Very often | 390 (97.0) | 12 (3.0) | 0.57 (0.29-1.02, p=0.078) |
| **Institute of Public Health/Center for Disease Control** | | | |
| Some time | 1416 (95.7) | 64 (4.3) | Reference |
| Never | 565 (85.7) | 94 (14.3) | 3.68 (2.65-5.15, p<0.001) |
| Hardly ever | 391 (92.2) | 33 (7.8) | 1.87 (1.20-2.86, p=0.005) |
| Very little | 495 (95.0) | 26 (5.0) | 1.16 (0.72-1.83, p=0.528) |
| Often enough | 1147 (96.2) | 45 (3.8) | 0.87 (0.59-1.28, p=0.476) |
| Often | 618 (99.2) | 5 (0.8) | 0.18 (0.06-0.40, p<0.001) |
| Very often | 349 (96.9) | 11 (3.1) | 0.70 (0.34-1.28, p=0.277) |
| **Celebrities and social media influencers** | | | |
| Some time | 498 (93.6) | 34 (6.4) | Reference |
| Never | 2758 (94.5) | 162 (5.5) | 0.86 (0.59-1.28, p=0.440) |
| Hardly ever | 705 (96.2) | 28 (3.8) | 0.58 (0.35-0.97, p=0.039) |
| Very little | 574 (96.6) | 20 (3.4) | 0.51 (0.29-0.89, p=0.020) |
| Often enough | 245 (91.8) | 22 (8.2) | 1.32 (0.74-2.28, p=0.336) |
| Often | 129 (95.6) | 6 (4.4) | 0.68 (0.25-1.55, p=0.398) |
| Very often | 72 (92.3) | 6 (7.7) | 1.22 (0.45-2.81, p=0.665) |
| **World Health Organization (WHO)** | | | |
| Some time | 1425 (96.0) | 59 (4.0) | Reference |
| Never | 639 (87.1) | 95 (12.9) | 3.59 (2.57-5.06, p<0.001) |
| Hardly ever | 443 (94.1) | 28 (5.9) | 1.53 (0.95-2.40, p=0.073) |
| Very little | 589 (94.8) | 32 (5.2) | 1.31 (0.84-2.02, p=0.227) |
| Often enough | 995 (96.1) | 40 (3.9) | 0.97 (0.64-1.46, p=0.888) |
| Often | 560 (97.9) | 12 (2.1) | 0.52 (0.26-0.94, p=0.040) |
| Very often | 330 (96.5) | 12 (3.5) | 0.88 (0.45-1.59, p=0.687) |
| **COVID-19 Hotlines** | | | |
| Some time | 576 (94.9) | 31 (5.1) | Reference |
| Never | 2513 (94.0) | 160 (6.0) | 1.18 (0.81-1.79, p=0.404) |
| Hardly ever | 727 (96.9) | 23 (3.1) | 0.59 (0.34-1.02, p=0.058) |
| Very little | 583 (95.0) | 31 (5.0) | 0.99 (0.59-1.65, p=0.963) |
| Often enough | 309 (96.0) | 13 (4.0) | 0.78 (0.39-1.48, p=0.466) |
| Often | 177 (92.2) | 15 (7.8) | 1.57 (0.81-2.94, p=0.164) |
| Very often | 96 (95.0) | 5 (5.0) | 0.97 (0.32-2.35, p=0.947) |
| **National COVID-19 information website** | | | |
| Some time | 1484 (95.4) | 72 (4.6) | Reference |
| Never | 648 (89.4) | 77 (10.6) | 2.45 (1.75-3.43, p<0.001) |
| Hardly ever | 453 (93.8) | 30 (6.2) | 1.36 (0.87-2.10, p=0.165) |
| Very little | 562 (94.5) | 33 (5.5) | 1.21 (0.78-1.83, p=0.377) |
| Often enough | 1008 (96.0) | 42 (4.0) | 0.86 (0.58-1.26, p=0.443) |
| Often | 563 (96.7) | 19 (3.3) | 0.70 (0.40-1.14, p=0.167) |
| Very often | 263 (98.1) | 5 (1.9) | 0.39 (0.14-0.89, p=0.045) |
| **How often do you seek information about COVID-19** | | | |
| Some time | 1673 (94.9) | 90 (5.1) | Reference |
| Never | 275 (83.8) | 53 (16.2) | 3.58 (2.48-5.13, p<0.001) |
| Hardly ever | 402 (92.2) | 34 (7.8) | 1.57 (1.03-2.34, p=0.030) |
| Very little | 569 (94.5) | 33 (5.5) | 1.08 (0.71-1.61, p=0.719) |
| Often enough | 1246 (96.7) | 43 (3.3) | 0.64 (0.44-0.92, p=0.019) |
| Often | 463 (97.3) | 13 (2.7) | 0.52 (0.28-0.91, p=0.031) |
| Very often | 353 (96.7) | 12 (3.3) | 0.63 (0.33-1.12, p=0.142) |
| **How much trust you have that the following institutions or persons can effectively manage the COVID-19?** | | | |
| **Your family doctor** | | | |
| Neither do I trust nor do I trust | 817 (92.1) | 70 (7.9) | Reference |
| Do not trust at all | 125 (82.2) | 27 (17.8) | 2.52 (1.54-4.04, p<0.001) |
| Trust very little | 146 (89.0) | 18 (11.0) | 1.44 (0.81-2.44, p=0.192) |
| Trust little | 323 (92.8) | 25 (7.2) | 0.90 (0.55-1.43, p=0.675) |
| Trust enough | 1899 (95.5) | 89 (4.5) | 0.55 (0.40-0.76, p<0.001) |
| Trust a lot | 1126 (96.7) | 38 (3.3) | 0.39 (0.26-0.59, p<0.001) |
| Trust very much | 545 (98.0) | 11 (2.0) | 0.24 (0.12-0.43, p<0.001) |
| **Hospitals** | | | |
| Neither do I trust nor do I trust | 856 (92.5) | 69 (7.5) | Reference |
| Do not trust at all | 113 (78.5) | 31 (21.5) | 3.40 (2.11-5.39, p<0.001) |
| Trust very little | 142 (86.6) | 22 (13.4) | 1.92 (1.13-3.16, p=0.012) |
| Trust little | 371 (93.0) | 28 (7.0) | 0.94 (0.59-1.46, p=0.777) |
| Trust enough | 1892 (95.8) | 82 (4.2) | 0.54 (0.39-0.75, p<0.001) |
| Trust a lot | 1149 (97.0) | 36 (3.0) | 0.39 (0.25-0.58, p<0.001) |
| Trust very much | 458 (97.9) | 10 (2.1) | 0.27 (0.13-0.51, p<0.001) |
| **Ministry of Health** | | | |
| Neither do I trust nor do I trust | 962 (94.0) | 61 (6.0) | Reference |
| Do not trust at all | 218 (77.3) | 64 (22.7) | 4.63 (3.17-6.78, p<0.001) |
| Trust very little | 164 (87.2) | 24 (12.8) | 2.31 (1.38-3.76, p=0.001) |
| Trust little | 387 (93.3) | 28 (6.7) | 1.14 (0.71-1.79, p=0.576) |
| Trust enough | 1843 (96.6) | 64 (3.4) | 0.55 (0.38-0.79, p=0.001) |
| Trust a lot | 994 (97.3) | 28 (2.7) | 0.44 (0.28-0.69, p<0.001) |
| Trust very much | 413 (97.9) | 9 (2.1) | 0.34 (0.16-0.66, p=0.003) |
| **Institute of Public Health /Center for disease Control** | | | |
| Neither do I trust nor do I trust | 901 (92.7) | 71 (7.3) | Reference |
| Do not trust at all | 183 (75.0) | 61 (25.0) | 4.23 (2.90-6.17, p<0.001) |
| Trust very little | 155 (90.1) | 17 (9.9) | 1.39 (0.78-2.37, p=0.244) |
| Trust little | 373 (94.4) | 22 (5.6) | 0.75 (0.45-1.21, p=0.250) |
| Trust enough | 1848 (96.4) | 69 (3.6) | 0.47 (0.34-0.67, p<0.001) |
| Trust a lot | 1054 (97.4) | 28 (2.6) | 0.34 (0.21-0.52, p<0.001) |
| Trust very much | 467 (97.9) | 10 (2.1) | 0.27 (0.13-0.51, p<0.001) |
| **Schools** | | | |
| Neither do I trust nor do I trust | 1462 (94.9) | 78 (5.1) | Reference |
| Do not trust at all | 348 (89.9) | 39 (10.1) | 2.10 (1.39-3.12, p<0.001) |
| Trust very little | 400 (93.0) | 30 (7.0) | 1.41 (0.90-2.15, p=0.125) |
| Trust little | 762 (95.7) | 34 (4.3) | 0.84 (0.55-1.25, p=0.395) |
| Trust enough | 1386 (95.7) | 63 (4.3) | 0.85 (0.60-1.20, p=0.356) |
| Trust a lot | 464 (94.5) | 27 (5.5) | 1.09 (0.68-1.69, p=0.705) |
| Trust very much | 159 (95.8) | 7 (4.2) | 0.83 (0.34-1.70, p=0.634) |
| **Public transportation companies** | | | |
| Neither do I trust nor do I trust | 1229 (94.1) | 77 (5.9) | Reference |
| Do not trust at all | 942 (93.7) | 63 (6.3) | 1.07 (0.76-1.50, p=0.710) |
| Trust very little | 722 (95.5) | 34 (4.5) | 0.75 (0.49-1.13, p=0.176) |
| Trust little | 1228 (96.2) | 49 (3.8) | 0.64 (0.44-0.92, p=0.016) |
| Trust enough | 593 (94.0) | 38 (6.0) | 1.02 (0.68-1.52, p=0.912) |
| Trust a lot | 189 (95.0) | 10 (5.0) | 0.84 (0.40-1.59, p=0.624) |
| Trust very much | 78 (91.8) | 7 (8.2) | 1.43 (0.58-3.01, p=0.383) |
| **Police** | | | |
| Neither do I trust nor do I trust | 1297 (94.5) | 76 (5.5) | Reference |
| Do not trust at all | 189 (80.1) | 47 (19.9) | 4.24 (2.85-6.28, p<0.001) |
| Trust very little | 241 (89.9) | 27 (10.1) | 1.91 (1.19-2.99, p=0.006) |
| Trust little | 488 (94.9) | 26 (5.1) | 0.91 (0.57-1.42, p=0.683) |
| Trust enough | 1704 (96.1) | 69 (3.9) | 0.69 (0.49-0.96, p=0.030) |
| Trust a lot | 709 (96.6) | 25 (3.4) | 0.60 (0.37-0.94, p=0.031) |
| Trust very much | 353 (97.8) | 8 (2.2) | 0.39 (0.17-0.76, p=0.012) |
| **Your employer** | | | |
| Neither do I trust nor do I trust | 897 (94.7) | 50 (5.3) | Reference |
| Do not trust at all | 266 (89.0) | 33 (11.0) | 2.23 (1.39-3.51, p=0.001) |
| Trust very little | 164 (93.2) | 12 (6.8) | 1.31 (0.65-2.44, p=0.413) |
| Trust little | 324 (94.7) | 18 (5.3) | 1.00 (0.56-1.70, p=0.991) |
| Trust enough | 1053 (95.3) | 52 (4.7) | 0.89 (0.59-1.32, p=0.551) |
| Trust a lot | 573 (96.3) | 22 (3.7) | 0.69 (0.41-1.13, p=0.154) |
| Trust very much | 369 (94.6) | 21 (5.4) | 1.02 (0.59-1.70, p=0.938) |
| Not applicable | 1335 (95.0) | 70 (5.0) | 0.94 (0.65-1.37, p=0.748) |
| **Your church/place of worship** | | | |
| Neither do I trust nor do I trust | 1257 (95.4) | 60 (4.6) | Reference |
| Do not trust at all | 673 (91.1) | 66 (8.9) | 2.05 (1.43-2.96, p<0.001) |
| Trust very little | 249 (95.0) | 13 (5.0) | 1.09 (0.57-1.96, p=0.775) |
| Trust little | 443 (95.9) | 19 (4.1) | 0.90 (0.52-1.49, p=0.691) |
| Trust enough | 892 (94.7) | 50 (5.3) | 1.17 (0.80-1.72, p=0.413) |
| Trust a lot | 382 (96.2) | 15 (3.8) | 0.82 (0.45-1.43, p=0.507) |
| Trust very much | 250 (95.4) | 12 (4.6) | 1.01 (0.51-1.83, p=0.986) |
| Not applicable | 835 (95.1) | 43 (4.9) | 1.08 (0.72-1.61, p=0.711) |
| **What do you think about the decisions made in Italy during the epidemic?** | | | |
| **Many very important things happen in the world, which the public is never informed about** | | | |
| Neither false nor true | 1052 (95.0) | 55 (5.0) | Reference |
| Completely false | 111 (94.1) | 7 (5.9) | 1.21 (0.49-2.54, p=0.650) |
| False | 160 (96.4) | 6 (3.6) | 0.72 (0.27-1.57, p=0.448) |
| Fairly false | 293 (94.5) | 17 (5.5) | 1.11 (0.62-1.90, p=0.715) |
| True enough | 1639 (96.0) | 68 (4.0) | 0.79 (0.55-1.15, p=0.213) |
| True | 854 (95.4) | 41 (4.6) | 0.92 (0.60-1.39, p=0.687) |
| Completely true | 872 (91.2) | 84 (8.8) | 1.84 (1.30-2.63, p=0.001) |
| **Politicians usually do not tell us the true motives for their decisions** | | | |
| Neither false nor true | 995 (95.3) | 49 (4.7) | Reference |
| Completely false | 105 (93.8) | 7 (6.2) | 1.35 (0.55-2.88, p=0.468) |
| False | 130 (94.2) | 8 (5.8) | 1.25 (0.54-2.56, p=0.570) |
| Fairly false | 241 (94.1) | 15 (5.9) | 1.26 (0.67-2.24, p=0.441) |
| True enough | 1574 (96.2) | 63 (3.8) | 0.81 (0.56-1.20, p=0.287) |
| True | 960 (95.7) | 43 (4.3) | 0.91 (0.60-1.38, p=0.657) |
| Completely true | 976 (91.3) | 93 (8.7) | 1.93 (1.36-2.78, p<0.001) |
| **Government agencies closely monitor all citizens** | | | |
| Neither false nor true | 1518 (94.9) | 81 (5.1) | Reference |
| Completely false | 341 (95.8) | 15 (4.2) | 0.82 (0.45-1.41, p=0.502) |
| False | 450 (96.8) | 15 (3.2) | 0.62 (0.34-1.06, p=0.100) |
| Fairly false | 634 (95.1) | 33 (4.9) | 0.98 (0.64-1.46, p=0.907) |
| True enough | 1171 (95.7) | 52 (4.3) | 0.83 (0.58-1.18, p=0.313) |
| True | 491 (93.7) | 33 (6.3) | 1.26 (0.82-1.89, p=0.279) |
| Completely true | 376 (88.5) | 49 (11.5) | 2.44 (1.67-3.53, p<0.001) |
| **Events which superficially seem to lack a connection are often the result of secret activities** | | | |
| Neither false nor true | 1824 (95.3) | 89 (4.7) | Reference |
| Completely false | 385 (95.8) | 17 (4.2) | 0.90 (0.52-1.50, p=0.712) |
| False | 453 (97.0) | 14 (3.0) | 0.63 (0.34-1.09, p=0.118) |
| Fairly false | 504 (95.6) | 23 (4.4) | 0.94 (0.57-1.47, p=0.780) |
| True enough | 1042 (94.6) | 59 (5.4) | 1.16 (0.82-1.62, p=0.388) |
| True | 445 (93.9) | 29 (6.1) | 1.34 (0.85-2.03, p=0.189) |
| Completely true | 328 (87.5) | 47 (12.5) | 2.94 (2.01-4.24, p<0.001) |
| **There are secret organizations that greatly influence political** | | | |
| Neither false nor true | 1474 (95.5) | 70 (4.5) | Reference |
| Completely false | 446 (97.2) | 13 (2.8) | 0.61 (0.32-1.08, p=0.112) |
| False | 412 (96.0) | 17 (4.0) | 0.87 (0.49-1.46, p=0.611) |
| Fairly false | 459 (95.4) | 22 (4.6) | 1.01 (0.60-1.62, p=0.971) |
| True enough | 1142 (94.3) | 69 (5.7) | 1.27 (0.90-1.79, p=0.167) |
| True | 552 (94.8) | 30 (5.2) | 1.14 (0.73-1.76, p=0.547) |
| Completely true | 496 (89.7) | 57 (10.3) | 2.42 (1.68-3.48, p<0.001) |
| **Please describe your experience during the COVID-10 epidemic:** | | | |
| **I have a hard time making it through stressful events** | | | |
| Neither disagree nor agree | 1530 (94.4) | 90 (5.6) | Reference |
| Strongly disagree | 301 (92.0) | 26 (8.0) | 1.47 (0.92-2.28, p=0.097) |
| Disagree | 535 (95.0) | 28 (5.0) | 0.89 (0.57-1.36, p=0.599) |
| Quite disagree | 887 (95.1) | 46 (4.9) | 0.88 (0.61-1.26, p=0.498) |
| Agree enough | 1132 (95.2) | 57 (4.8) | 0.86 (0.61-1.20, p=0.371) |
| Agree | 351 (95.6) | 16 (4.4) | 0.77 (0.43-1.30, p=0.358) |
| Strongly agree | 245 (94.2) | 15 (5.8) | 1.04 (0.57-1.77, p=0.889) |
| **It does not take me long to recover from a stressful event** | | | |
| Neither disagree nor agree | 1458 (93.5) | 102 (6.5) | Reference |
| Strongly disagree | 241 (90.9) | 24 (9.1) | 1.42 (0.88-2.23, p=0.137) |
| Disagree | 438 (98.2) | 8 (1.8) | 0.26 (0.12-0.51, p<0.001) |
| Quite disagree | 802 (95.7) | 36 (4.3) | 0.64 (0.43-0.94, p=0.026) |
| Agree enough | 1370 (95.5) | 64 (4.5) | 0.67 (0.48-0.92, p=0.014) |
| Agree | 452 (94.6) | 26 (5.4) | 0.82 (0.52-1.26, p=0.387) |
| Strongly agree | 220 (92.4) | 18 (7.6) | 1.17 (0.67-1.92, p=0.556) |
| **It is hard for me to snap back when something bad happens** | | | |
| Neither disagree nor agree | 1463 (93.5) | 102 (6.5) | Reference |
| Strongly disagree | 234 (90.3) | 25 (9.7) | 1.53 (0.95-2.39, p=0.068) |
| Disagree | 450 (95.3) | 22 (4.7) | 0.70 (0.43-1.10, p=0.141) |
| Quite disagree | 818 (95.0) | 43 (5.0) | 0.75 (0.52-1.08, p=0.131) |
| Agree enough | 1299 (95.8) | 57 (4.2) | 0.63 (0.45-0.87, p=0.006) |
| Agree | 407 (96.7) | 14 (3.3) | 0.49 (0.27-0.84, p=0.015) |
| Strongly agree | 310 (95.4) | 15 (4.6) | 0.69 (0.38-1.17, p=0.198) |
| **If you have been in contact with someone who tested positive for COVID-19 and have no symptoms yourself – will you get tested if you have the opportunity?** | | | |
| I would take the test for sure | 4586 (97.2) | 131 (2.8) | Reference |
| I probably wouldn't take the test | 395 (72.9) | 147 (27.1) | 13.03 (10.08-16.86, p<0.001) |
| **Please express your opinion regarding the decisions made in Italy to reduce the spread of COVID-19:** | | | |
| **I think the decisions are fair** | | | |
| Neither disagree nor agree | 1216 (93.9) | 79 (6.1) | Reference |
| Strongly disagree | 310 (81.4) | 71 (18.6) | 3.53 (2.50-4.97, p<0.001) |
| Disagree | 256 (91.4) | 24 (8.6) | 1.44 (0.88-2.29, p=0.131) |
| Quite disagree | 607 (94.3) | 37 (5.7) | 0.94 (0.62-1.39, p=0.756) |
| Agree enough | 1522 (97.2) | 44 (2.8) | 0.44 (0.30-0.64, p<0.001) |
| Agree | 647 (97.9) | 14 (2.1) | 0.33 (0.18-0.57, p<0.001) |
| Strongly agree | 423 (97.9) | 9 (2.1) | 0.33 (0.15-0.62, p=0.002) |
| **I would convince others that the decisions are right** | | | |
| Neither disagree nor agree | 1474 (94.4) | 87 (5.6) | Reference |
| Strongly disagree | 292 (80.0) | 73 (20.0) | 4.24 (3.02-5.92, p<0.001) |
| Disagree | 250 (90.9) | 25 (9.1) | 1.69 (1.05-2.66, p=0.026) |
| Quite disagree | 460 (94.3) | 28 (5.7) | 1.03 (0.65-1.58, p=0.890) |
| Agree enough | 1399 (97.0) | 43 (3.0) | 0.52 (0.36-0.75, p=0.001) |
| Agree | 684 (97.6) | 17 (2.4) | 0.42 (0.24-0.70, p=0.001) |
| Strongly agree | 422 (98.8) | 5 (1.2) | 0.20 (0.07-0.45, p=0.001) |
| **Please express your opinion about the following phrases:** | | | |
| **In the event of an outbreak it’s appropriate to avoid certain people on the basis of their ethnicity** | | | |
| Neither disagree nor agree | 997 (92.2) | 84 (7.8) | Reference |
| Strongly disagree | 1741 (95.1) | 89 (4.9) | 0.61 (0.45-0.83, p=0.001) |
| Disagree | 782 (97.4) | 21 (2.6) | 0.32 (0.19-0.51, p<0.001) |
| Quite disagree | 518 (95.6) | 24 (4.4) | 0.55 (0.34-0.86, p=0.012) |
| Agree enough | 426 (93.4) | 30 (6.6) | 0.84 (0.53-1.27, p=0.416) |
| Agree | 266 (93.7) | 18 (6.3) | 0.80 (0.46-1.33, p=0.415) |
| Strongly agree | 251 (95.4) | 12 (4.6) | 0.57 (0.29-1.02, p=0.073) |
| **I think that the restrictions currently being implemented are greatly exaggerated** | | | |
| Neither disagree nor agree | 1171 (94.5) | 68 (5.5) | Reference |
| Strongly disagree | 762 (97.4) | 20 (2.6) | 0.45 (0.27-0.74, p=0.002) |
| Disagree | 862 (98.4) | 14 (1.6) | 0.28 (0.15-0.49, p<0.001) |
| Quite disagree | 884 (97.4) | 24 (2.6) | 0.47 (0.29-0.74, p=0.002) |
| Agree enough | 682 (92.5) | 55 (7.5) | 1.39 (0.96-2.00, p=0.080) |
| Agree | 321 (92.8) | 25 (7.2) | 1.34 (0.82-2.13, p=0.226) |
| Strongly agree | 299 (80.6) | 72 (19.4) | 4.15 (2.91-5.92, p<0.001) |
| **The government should be allowed to force people into self-isolation if they have been in contact with someone who was infected** | | | |
| Neither disagree nor agree | 1024 (92.3) | 86 (7.7) | Reference |
| Strongly disagree | 144 (70.6) | 60 (29.4) | 4.96 (3.41-7.20, p<0.001) |
| Disagree | 122 (88.4) | 16 (11.6) | 1.56 (0.86-2.68, p=0.123) |
| Quite disagree | 228 (87.7) | 32 (12.3) | 1.67 (1.07-2.55, p=0.019) |
| Agree enough | 1479 (96.7) | 50 (3.3) | 0.40 (0.28-0.57, p<0.001) |
| Agree | 970 (98.1) | 19 (1.9) | 0.23 (0.14-0.38, p<0.001) |
| Strongly agree | 1014 (98.5) | 15 (1.5) | 0.18 (0.10-0.30, p<0.001) |
| **More tests for coronavirus infection should be carried out in the population** | | | |
| Neither disagree nor agree | 985 (92.2) | 83 (7.8) | Reference |
| Strongly disagree | 71 (62.8) | 42 (37.2) | 7.02 (4.49-10.91, p<0.001) |
| Disagree | 81 (83.5) | 16 (16.5) | 2.34 (1.27-4.10, p=0.004) |
| Quite disagree | 133 (84.2) | 25 (15.8) | 2.23 (1.35-3.57, p=0.001) |
| Agree enough | 1619 (96.4) | 60 (3.6) | 0.44 (0.31-0.62, p<0.001) |
| Agree | 1021 (97.5) | 26 (2.5) | 0.30 (0.19-0.47, p<0.001) |
| Strongly agree | 1071 (97.6) | 26 (2.4) | 0.29 (0.18-0.44, p<0.001) |
| **I am worried that the pandemic will have economic consequences for me in the future** | | | |
| Neither disagree nor agree | 1292 (94.5) | 75 (5.5) | Reference |
| Strongly disagree | 121 (85.8) | 20 (14.2) | 2.85 (1.64-4.74, p<0.001) |
| Disagree | 174 (95.6) | 8 (4.4) | 0.79 (0.35-1.57, p=0.540) |
| Quite disagree | 291 (95.1) | 15 (4.9) | 0.89 (0.48-1.52, p=0.682) |
| Agree enough | 1385 (96.3) | 53 (3.7) | 0.66 (0.46-0.94, p=0.023) |
| Agree | 702 (94.4) | 42 (5.6) | 1.03 (0.69-1.51, p=0.879) |
| Strongly agree | 1016 (94.0) | 65 (6.0) | 1.10 (0.78-1.55, p=0.578) |
| **Please indicate your opinion about the following anti-epidemic measures:** | | | |
| **Compulsory face masks in closed public spaces** | | | |
| Neither disagree nor agree | 492 (89.5) | 58 (10.5) | Reference |
| Strongly disagree | 144 (72.4) | 55 (27.6) | 3.24 (2.14-4.90, p<0.001) |
| Disagree | 119 (89.5) | 14 (10.5) | 1.00 (0.52-1.80, p=0.995) |
| Quite disagree | 189 (87.9) | 26 (12.1) | 1.17 (0.70-1.89, p=0.539) |
| Agree enough | 692 (94.3) | 42 (5.7) | 0.51 (0.34-0.78, p=0.002) |
| Agree | 930 (96.2) | 37 (3.8) | 0.34 (0.22-0.51, p<0.001) |
| Strongly agree | 2415 (98.1) | 46 (1.9) | 0.16 (0.11-0.24, p<0.001) |
| **Restricting restaurants to outside spaces** | | | |
| Neither disagree nor agree | 889 (94.5) | 52 (5.5) | Reference |
| Strongly disagree | 496 (84.9) | 88 (15.1) | 3.03 (2.12-4.37, p<0.001) |
| Disagree | 362 (93.8) | 24 (6.2) | 1.13 (0.68-1.85, p=0.623) |
| Quite disagree | 624 (94.7) | 35 (5.3) | 0.96 (0.61-1.48, p=0.852) |
| Agree enough | 949 (95.4) | 46 (4.6) | 0.83 (0.55-1.24, p=0.366) |
| Agree | 805 (98.1) | 16 (1.9) | 0.34 (0.19-0.59, p<0.001) |
| Strongly agree | 856 (98.1) | 17 (1.9) | 0.34 (0.19-0.58, p<0.001) |
| **Distance learning in schools** | | | |
| Neither disagree nor agree | 888 (93.4) | 63 (6.6) | Reference |
| Strongly disagree | 396 (84.3) | 74 (15.7) | 2.63 (1.85-3.77, p<0.001) |
| Disagree | 341 (94.5) | 20 (5.5) | 0.83 (0.48-1.36, p=0.472) |
| Quite disagree | 549 (95.0) | 29 (5.0) | 0.74 (0.47-1.16, p=0.201) |
| Agree enough | 1044 (96.3) | 40 (3.7) | 0.54 (0.36-0.81, p=0.003) |
| Agree | 737 (96.6) | 26 (3.4) | 0.50 (0.31-0.78, p=0.003) |
| Strongly agree | 1026 (97.5) | 26 (2.5) | 0.36 (0.22-0.56, p<0.001) |
| **Curfew from 22:00 to 5:00** | | | |
| Neither disagree nor agree | 787 (92.9) | 60 (7.1) | Reference |
| Strongly disagree | 403 (81.4) | 92 (18.6) | 2.99 (2.12-4.25, p<0.001) |
| Disagree | 338 (94.7) | 19 (5.3) | 0.74 (0.42-1.23, p=0.261) |
| Quite disagree | 459 (95.4) | 22 (4.6) | 0.63 (0.37-1.02, p=0.070) |
| Agree enough | 956 (95.5) | 45 (4.5) | 0.62 (0.41-0.92, p=0.018) |
| Agree | 885 (97.3) | 25 (2.7) | 0.37 (0.23-0.59, p<0.001) |
| Strongly agree | 1153 (98.7) | 15 (1.3) | 0.17 (0.09-0.29, p<0.001) |
| **Ban on mass gatherings in streets** | | | |
| Neither disagree nor agree | 463 (88.5) | 60 (11.5) | Reference |
| Strongly disagree | 82 (64.6) | 45 (35.4) | 4.23 (2.69-6.66, p<0.001) |
| Disagree | 73 (83.9) | 14 (16.1) | 1.48 (0.76-2.72, p=0.224) |
| Quite disagree | 127 (90.1) | 14 (9.9) | 0.85 (0.44-1.53, p=0.606) |
| Agree enough | 846 (94.3) | 51 (5.7) | 0.47 (0.31-0.69, p<0.001) |
| Agree | 1067 (95.7) | 48 (4.3) | 0.35 (0.23-0.51, p<0.001) |
| Strongly agree | 2323 (98.1) | 46 (1.9) | 0.15 (0.10-0.23, p<0.001) |
| **Introduction of mandatory testing of school teachers** | | | |
| Neither disagree nor agree | 618 (90.2) | 67 (9.8) | Reference |
| Strongly disagree | 88 (65.2) | 47 (34.8) | 4.93 (3.18-7.60, p<0.001) |
| Disagree | 64 (84.2) | 12 (15.8) | 1.73 (0.85-3.26, p=0.107) |
| Quite disagree | 108 (84.4) | 20 (15.6) | 1.71 (0.97-2.88, p=0.052) |
| Agree enough | 1074 (95.2) | 54 (4.8) | 0.46 (0.32-0.67, p<0.001) |
| Agree | 1201 (96.2) | 48 (3.8) | 0.37 (0.25-0.54, p<0.001) |
| Strongly agree | 1828 (98.4) | 30 (1.6) | 0.15 (0.10-0.23, p<0.001) |
| **Opening borders to more countries** | | | |
| Neither disagree nor agree | 753 (93.3) | 54 (6.7) | Reference |
| Strongly disagree | 119 (75.3) | 39 (24.7) | 4.57 (2.89-7.19, p<0.001) |
| Disagree | 117 (87.3) | 17 (12.7) | 2.03 (1.11-3.55, p=0.017) |
| Quite disagree | 186 (90.3) | 20 (9.7) | 1.50 (0.86-2.53, p=0.140) |
| Agree enough | 1111 (95.9) | 48 (4.1) | 0.60 (0.40-0.90, p=0.013) |
| Agree | 972 (95.7) | 44 (4.3) | 0.63 (0.42-0.95, p=0.028) |
| Strongly agree | 1723 (96.9) | 56 (3.1) | 0.45 (0.31-0.67, p<0.001) |
| **During the past 2 weeks, you made the following:** | | | |
| **Avoided people that I thought might infect me, based on their ethnicity** | | | |
| No | 3598 (95.1) | 187 (4.9) | Reference |
| Yes | 636 (94.2) | 39 (5.8) | 1.18 (0.82-1.66, p=0.361) |
| Not applicable | 747 (93.5) | 52 (6.5) | 1.34 (0.97-1.83, p=0.071) |
| **Exercised less than I did before the pandemic** | | | |
| No | 2144 (94.3) | 130 (5.7) | Reference |
| Yes | 2164 (95.9) | 92 (4.1) | 0.70 (0.53-0.92, p=0.011) |
| Not applicable | 673 (92.3) | 56 (7.7) | 1.37 (0.98-1.89, p=0.056) |
| **Drank more alcohol than I did before the pandemic** | | | |
| No | 3793 (95.3) | 187 (4.7) | Reference |
| Yes | 460 (93.7) | 31 (6.3) | 1.37 (0.91-1.99, p=0.118) |
| Not applicable | 728 (92.4) | 60 (7.6) | 1.67 (1.23-2.25, p=0.001) |
| **Ate more unhealthy food than I did before the pandemic** | | | |
| No | 3260 (95.2) | 164 (4.8) | Reference |
| Yes | 1393 (95.1) | 72 (4.9) | 1.03 (0.77-1.36, p=0.852) |
| Not applicable | 328 (88.6) | 42 (11.4) | 2.55 (1.76-3.61, p<0.001) |
| **Postponed vaccination for myself or my child** | | | |
| Yes | 302 (94.7) | 17 (5.3) | Reference |
| No | 3292 (95.4) | 160 (4.6) | 0.86 (0.53-1.49, p=0.575) |
| Not applicable | 1387 (93.2) | 101 (6.8) | 1.29 (0.78-2.27, p=0.340) |
| **Avoided going to the doctor for a non-COVID-19-related problem** | | | |
| Yes | 1678 (96.1) | 68 (3.9) | Reference |
| No | 2743 (94.7) | 153 (5.3) | 1.38 (1.03-1.85, p=0.032) |
| Not applicable | 560 (90.8) | 57 (9.2) | 2.51 (1.74-3.61, p<0.001) |
| **Bought drugs that I heard are good for treating COVID-19** | | | |
| Yes | 307 (92.5) | 25 (7.5) | Reference |
| No | 4223 (95.3) | 210 (4.7) | 0.61 (0.40-0.96, p=0.025) |
| Not applicable | 451 (91.3) | 43 (8.7) | 1.17 (0.71-1.98, p=0.547) |
| **Please indicate your general well-being during the past 2 weeks:** | | | |
| **I have felt cheerful and in good spirits** | | | |
| More than half the time | 1247 (95.9) | 53 (4.1) | Reference |
| All time | 166 (90.7) | 17 (9.3) | 2.41 (1.33-4.18, p=0.002) |
| Most of the time | 690 (96.0) | 29 (4.0) | 0.99 (0.62-1.56, p=0.962) |
| Less than half the time | 1225 (93.9) | 79 (6.1) | 1.52 (1.07-2.18, p=0.022) |
| Some time | 1306 (94.6) | 74 (5.4) | 1.33 (0.93-1.92, p=0.119) |
| In no time | 347 (93.0) | 26 (7.0) | 1.76 (1.07-2.83, p=0.022) |
| **I have felt calm and relaxed** | | | |
| Some time | 1218 (95.3) | 60 (4.7) | Reference |
| All time | 191 (89.7) | 22 (10.3) | 2.34 (1.38-3.85, p=0.001) |
| Most of the time | 754 (94.8) | 41 (5.2) | 1.10 (0.73-1.65, p=0.635) |
| More than half the time | 1204 (94.7) | 68 (5.3) | 1.15 (0.80-1.64, p=0.452) |
| Less than half the time | 1185 (95.1) | 61 (4.9) | 1.04 (0.72-1.51, p=0.813) |
| In no time | 429 (94.3) | 26 (5.7) | 1.23 (0.75-1.95, p=0.391) |
| **I have felt active and vigorous** | | | |
| More than half the time | 1175 (94.8) | 64 (5.2) | Reference |
| All time | 207 (91.2) | 20 (8.8) | 1.77 (1.03-2.94, p=0.032) |
| Most of the time | 714 (94.3) | 43 (5.7) | 1.11 (0.74-1.64, p=0.620) |
| Less than half the time | 1254 (94.9) | 68 (5.1) | 1.00 (0.70-1.42, p=0.980) |
| Some time | 1186 (95.1) | 61 (4.9) | 0.94 (0.66-1.35, p=0.755) |
| In no time | 445 (95.3) | 22 (4.7) | 0.91 (0.54-1.47, p=0.702) |
| **I woke up feeling fresh and rested** | | | |
| Some time | 1298 (95.6) | 60 (4.4) | Reference |
| All time | 204 (91.1) | 20 (8.9) | 2.12 (1.22-3.53, p=0.005) |
| Most of the time | 793 (95.7) | 36 (4.3) | 0.98 (0.64-1.49, p=0.933) |
| More than half the time | 1031 (94.9) | 55 (5.1) | 1.15 (0.79-1.68, p=0.454) |
| Less than half the time | 1091 (93.5) | 76 (6.5) | 1.51 (1.07-2.14, p=0.021) |
| In no time | 564 (94.8) | 31 (5.2) | 1.19 (0.75-1.84, p=0.445) |
| **My daily life has been filled with things that interest me** | | | |
| Some time | 1214 (95.1) | 62 (4.9) | Reference |
| All time | 245 (90.7) | 25 (9.3) | 2.00 (1.21-3.20, p=0.005) |
| Most of the time | 768 (95.5) | 36 (4.5) | 0.92 (0.60-1.39, p=0.689) |
| More than half the time | 1247 (95.1) | 64 (4.9) | 1.00 (0.70-1.44, p=0.978) |
| Less than half the time | 1186 (94.6) | 68 (5.4) | 1.12 (0.79-1.60, p=0.521) |
| In no time | 321 (93.3) | 23 (6.7) | 1.40 (0.84-2.27, p=0.179) |
| **I believe a vaccine can help control the spread of COVID-19** | | | |
| Neither disagree nor agree | 696 (91.1) | 68 (8.9) | Reference |
| Strongly disagree | 128 (72.3) | 49 (27.7) | 3.92 (2.59-5.91, p<0.001) |
| Disagree | 116 (85.9) | 19 (14.1) | 1.68 (0.95-2.84, p=0.063) |
| Quite disagree | 148 (89.2) | 18 (10.8) | 1.24 (0.70-2.11, p=0.434) |
| Agree enough | 934 (95.2) | 47 (4.8) | 0.52 (0.35-0.75, p=0.001) |
| Agree | 943 (96.1) | 38 (3.9) | 0.41 (0.27-0.62, p<0.001) |
| Strongly agree | 2016 (98.1) | 39 (1.9) | 0.20 (0.13-0.29, p<0.001) |
| **Apart from COVID-19, I think everyone should be vaccinated according to the national vaccination schedule** | | | |
| Yes | 3750 (97.2) | 109 (2.8) | Reference |
| No | 480 (80.9) | 113 (19.1) | 8.10 (6.12-10.72, p<0.001) |
| Do not know | 751 (93.1) | 56 (6.9) | 2.57 (1.83-3.56, p<0.001) |

### S3 Table. Extended results of multivariable analysis in the training data set.

|  | **Response frequency, n(%)** | | **Generalized Linear Model analysis** | | | |
| --- | --- | --- | --- | --- | --- | --- |
|  | Would share | Would not share | **Univariable** | **20-variables model** | **10-variables model** | **6-variables model** |
|  |  |  | OR (95%CI) | OR (95%CI) | OR (95%CI) | OR (95%CI) |
| **If you have been in contact with someone who tested positive for COVID-19 and have no symptoms yourself – will you get tested if you have the opportunity?** | | | | | | |
| I would take the test for sure | 4586 (97.2) | 131 (2.8) | Reference | Reference | Reference | Reference |
| I probably wouldn't take the test | 395 (72.9) | 147 (27.1) | 13.03 (10.08-16.86, p<0.001) | 6.42 (4.54-9.08, p<0.001) | 5.40 (3.93-7.41, p<0.001) | 5.60 (4.14-7.58, p<0.001) |
| **The government should be allowed to force people into self-isolation if they have been in contact with someone who was infected** | | | | | | |
| Strongly disagree | 144 (70.6) | 60 (29.4) | 4.96 (3.41-7.20, p<0.001) | 1.92 (1.06-3.43, p=0.029) | 1.60 (0.95-2.67, p=0.076) | 1.79 (1.12-2.84, p=0.014) |
| Disagree | 122 (88.4) | 16 (11.6) | 1.56 (0.86-2.68, p=0.123) | 1.05 (0.50-2.09, p=0.896) | 0.90 (0.45-1.71, p=0.761) | 0.94 (0.49-1.73, p=0.859) |
| Quite disagree | 228 (87.7) | 32 (12.3) | 1.67 (1.07-2.55, p=0.019) | 1.82 (1.06-3.08, p=0.027) | 1.68 (1.02-2.71, p=0.036) | 1.60 (0.99-2.55, p=0.051) |
| Neither disagree nor agree | 1024 (92.3) | 86 (7.7) | Reference | Reference | Reference | Reference |
| Agree enough | 1479 (96.7) | 50 (3.3) | 0.40 (0.28-0.57, p<0.001) | 0.65 (0.42-0.99, p=0.044) | 0.62 (0.41-0.91, p=0.016) | 0.59 (0.40-0.86, p=0.007) |
| Agree | 970 (98.1) | 19 (1.9) | 0.23 (0.14-0.38, p<0.001) | 0.35 (0.19-0.63, p=0.001) | 0.39 (0.22-0.66, p=0.001) | 0.37 (0.21-0.62, p<0.001) |
| Strongly agree | 1014 (98.5) | 15 (1.5) | 0.18 (0.10-0.30, p<0.001) | 0.42 (0.21-0.80, p=0.011) | 0.38 (0.20-0.68, p=0.002) | 0.33 (0.18-0.57, p<0.001) |
| **Apart from COVID-19, I think everyone should be vaccinated according to the national vaccination schedule** | | | | | | |
| Yes | 3750 (97.2) | 109 (2.8) | Reference | Reference | Reference | Reference |
| No | 480 (80.9) | 113 (19.1) | 8.10 (6.12-10.72, p<0.001) | 2.07 (1.40-3.06, p<0.001) | 2.45 (1.70-3.52, p<0.001) | 2.63 (1.86-3.69, p<0.001) |
| Do not know | 751 (93.1) | 56 (6.9) | 2.57 (1.83-3.56, p<0.001) | 1.18 (0.79-1.76, p=0.415) | 1.27 (0.86-1.85, p=0.216) | 1.39 (0.96-1.99, p=0.075) |
| **Which measures you performed to prevent the COVID-19 infection in the last 7 days: Disinfected surfaces** | | | | |  |  |
| Never | 94 (66.7) | 47 (33.3) | 7.05 (4.51-11.02, p<0.001) | 4.79 (2.54-9.02, p<0.001) | 3.43 (1.91-6.15, p<0.001) | 3.23 (1.85-5.59, p<0.001) |
| Hardly ever | 123 (90.4) | 13 (9.6) | 1.49 (0.76-2.73, p=0.219) | 0.74 (0.31-1.62, p=0.464) | 0.73 (0.34-1.52, p=0.420) | 0.81 (0.38-1.63, p=0.576) |
| Very little | 211 (92.1) | 18 (7.9) | 1.20 (0.67-2.05, p=0.515) | 1.06 (0.51-2.07, p=0.879) | 0.97 (0.51-1.79, p=0.926) | 0.91 (0.48-1.66, p=0.767) |
| Some time | 761 (93.4) | 54 (6.6) | Reference | Reference | Reference | Reference |
| Often enough | 1069 (95.7) | 48 (4.3) | 0.63 (0.42-0.94, p=0.025) | 0.76 (0.47-1.23, p=0.265) | 0.74 (0.48-1.16, p=0.193) | 0.70 (0.45-1.08, p=0.108) |
| Often | 1125 (95.5) | 53 (4.5) | 0.66 (0.45-0.98, p=0.040) | 1.06 (0.66-1.69, p=0.819) | 0.98 (0.63-1.51, p=0.912) | 0.93 (0.61-1.42, p=0.728) |
| Very often | 1598 (97.3) | 45 (2.7) | 0.40 (0.26-0.59, p<0.001) | 0.64 (0.39-1.06, p=0.084) | 0.62 (0.39-0.97, p=0.038) | 0.56 (0.36-0.86, p=0.009) |
| **Ate more unhealthy food than I did before the pandemic** | | | |  |  |  |
| No | 3260 (95.2) | 164 (4.8) | Reference | Reference | Reference | Reference |
| Yes | 1393 (95.1) | 72 (4.9) | 1.03 (0.77-1.36, p=0.852) | 0.97 (0.67-1.39, p=0.855) | 1.26 (0.90-1.75, p=0.172) | 1.28 (0.93-1.76, p=0.127) |
| Not applicable | 328 (88.6) | 42 (11.4) | 2.55 (1.76-3.61, p<0.001) | 2.26 (1.37-3.66, p=0.001) | 2.64 (1.72-4.00, p<0.001) | 2.63 (1.73-3.94, p<0.001) |
| **Do you know people in your immediate social environment who are or have been infected with COVID-19 (suspected or confirmed)?** | | | | | | |
| Yes | 3678 (95.9) | 158 (4.1) | Reference | Reference | Reference | Reference |
| No | 1303 (91.6) | 120 (8.4) | 2.14 (1.67-2.74, p<0.001) | 1.62 (1.18-2.23, p=0.003) | 1.67 (1.24-2.23, p=0.001) | 1.66 (1.24-2.21, p=0.001) |
| **How often you use information from the Institute of Public Health/Center for Disease Control** | | | | |  |  |
| Never | 565 (85.7) | 94 (14.3) | 3.68 (2.65-5.15, p<0.001) | 1.83 (1.14-2.95, p=0.013) | 1.46 (0.96-2.20, p=0.073) |  |
| Hardly ever | 391 (92.2) | 33 (7.8) | 1.87 (1.20-2.86, p=0.005) | 1.39 (0.78-2.44, p=0.256) | 1.16 (0.69-1.90, p=0.563) |  |
| Very little | 495 (95.0) | 26 (5.0) | 1.16 (0.72-1.83, p=0.528) | 0.85 (0.48-1.48, p=0.585) | 0.75 (0.44-1.24, p=0.268) |  |
| Some time | 1416 (95.7) | 64 (4.3) | Reference | Reference | Reference |  |
| Often enough | 1147 (96.2) | 45 (3.8) | 0.87 (0.59-1.28, p=0.476) | 1.21 (0.75-1.94, p=0.442) | 1.33 (0.87-2.03, p=0.188) |  |
| Often | 618 (99.2) | 5 (0.8) | 0.18 (0.06-0.40, p<0.001) | 0.22 (0.07-0.57, p=0.004) | 0.33 (0.11-0.78, p=0.022) |  |
| Very often | 349 (96.9) | 11 (3.1) | 0.70 (0.34-1.28, p=0.277) | 1.19 (0.46-2.81, p=0.709) | 1.70 (0.77-3.44, p=0.162) |  |
| **I feel that epidemic spreading:** | |  |  |  |  |  |
| Slowly growing | 430 (90.5) | 45 (9.5) | 1.51 (1.03-2.20, p=0.032) | 0.73 (0.42-1.24, p=0.256) | 0.81 (0.49-1.32, p=0.406) |  |
| 2 | 405 (93.8) | 27 (6.2) | 0.96 (0.61-1.49, p=0.871) | 0.89 (0.50-1.53, p=0.683) | 0.97 (0.57-1.61, p=0.911) |  |
| 3 | 545 (94.0) | 35 (6.0) | 0.93 (0.61-1.38, p=0.720) | 1.15 (0.70-1.87, p=0.565) | 1.21 (0.76-1.91, p=0.405) |  |
| 4 | 1214 (93.5) | 84 (6.5) | Reference | Reference | Reference |  |
| 5 | 771 (94.6) | 44 (5.4) | 0.82 (0.56-1.19, p=0.315) | 1.20 (0.76-1.87, p=0.420) | 1.18 (0.77-1.79, p=0.437) |  |
| 6 | 654 (96.3) | 25 (3.7) | 0.55 (0.34-0.86, p=0.011) | 0.82 (0.46-1.40, p=0.475) | 0.85 (0.50-1.41, p=0.546) |  |
| Fast growing | 962 (98.2) | 18 (1.8) | 0.27 (0.16-0.44, p<0.001) | 0.33 (0.17-0.62, p=0.001) | 0.39 (0.21-0.69, p=0.002) |  |
| **It does not take me long to recover from a stressful event** | | | |  |  |  |
| Strongly disagree | 241 (90.9) | 24 (9.1) | 1.42 (0.88-2.23, p=0.137) | 1.72 (0.86-3.32, p=0.115) | 1.65 (0.89-2.96, p=0.100) |  |
| Disagree | 438 (98.2) | 8 (1.8) | 0.26 (0.12-0.51, p<0.001) | 0.27 (0.10-0.62, p=0.004) | 0.29 (0.12-0.62, p=0.003) |  |
| Quite disagree | 802 (95.7) | 36 (4.3) | 0.64 (0.43-0.94, p=0.026) | 0.90 (0.55-1.43, p=0.646) | 0.84 (0.53-1.29, p=0.428) |  |
| Neither disagree nor agree | 1458 (93.5) | 102 (6.5) | Reference | Reference | Reference |  |
| Agree enough | 1370 (95.5) | 64 (4.5) | 0.67 (0.48-0.92, p=0.014) | 0.93 (0.63-1.38, p=0.730) | 0.86 (0.59-1.24, p=0.420) |  |
| Agree | 452 (94.6) | 26 (5.4) | 0.82 (0.52-1.26, p=0.387) | 0.96 (0.52-1.72, p=0.900) | 1.02 (0.60-1.71, p=0.929) |  |
| Strongly agree | 220 (92.4) | 18 (7.6) | 1.17 (0.67-1.92, p=0.556) | 0.49 (0.20-1.10, p=0.092) | 0.45 (0.20-0.92, p=0.035) |  |
| **Estimate how easy/hard it was to understand recommendations about when to stay at home from work/school, and when not to?** | | | | | |  |
| Very difficult | 90 (71.4) | 36 (28.6) | 5.82 (3.62-9.28, p<0.001) | 3.46 (1.48-7.95, p=0.004) | 2.42 (1.28-4.53, p=0.006) |  |
| Hard | 133 (89.3) | 16 (10.7) | 1.75 (0.95-3.07, p=0.060) | 0.96 (0.40-2.19, p=0.925) | 1.63 (0.79-3.18, p=0.166) |  |
| Pretty hard | 496 (93.6) | 34 (6.4) | 1.00 (0.64-1.54, p=0.991) | 0.86 (0.48-1.55, p=0.629) | 1.05 (0.63-1.74, p=0.836) |  |
| Neither difficult nor easy | 844 (93.6) | 58 (6.4) | Reference | Reference | Reference |  |
| Quite easy | 1579 (96.3) | 61 (3.7) | 0.56 (0.39-0.81, p=0.002) | 1.18 (0.72-1.95, p=0.516) | 0.82 (0.54-1.25, p=0.352) |  |
| Easy | 1148 (95.4) | 55 (4.6) | 0.70 (0.48-1.02, p=0.062) | 2.20 (1.23-3.95, p=0.008) | 1.35 (0.87-2.09, p=0.186) |  |
| Very easy | 691 (97.5) | 18 (2.5) | 0.38 (0.22-0.64, p<0.001) | 0.87 (0.36-2.05, p=0.758) | 0.74 (0.38-1.37, p=0.352) |  |
| **Which measures you performed to prevent the COVID-19 infection in the last 7 days: Used medicines to prevent or treat COVID-19** | | | | | |  |
| Never | 3632 (95.4) | 174 (4.6) | 0.37 (0.25-0.56, p<0.001) | 0.32 (0.19-0.54, p<0.001) |  |  |
| Hardly ever | 359 (97.0) | 11 (3.0) | 0.24 (0.11-0.46, p<0.001) | 0.28 (0.12-0.62, p=0.002) |  |  |
| Very little | 271 (92.2) | 23 (7.8) | 0.66 (0.37-1.15, p=0.141) | 0.50 (0.24-1.00, p=0.053) |  |  |
| Some time | 247 (88.5) | 32 (11.5) | Reference | Reference |  |  |
| Often enough | 163 (89.1) | 20 (10.9) | 0.95 (0.52-1.70, p=0.857) | 0.81 (0.38-1.69, p=0.579) |  |  |
| Often | 139 (94.6) | 8 (5.4) | 0.44 (0.19-0.95, p=0.047) | 0.35 (0.12-0.92, p=0.041) |  |  |
| Very often | 170 (94.4) | 10 (5.6) | 0.45 (0.21-0.92, p=0.036) | 0.70 (0.27-1.67, p=0.433) |  |  |
| **What do you consider to be your own probability of getting infected with COVID-19?** | | | | |  |  |
| Very unlikely | 237 (88.8) | 30 (11.2) | 2.47 (1.61-3.70, p<0.001) | 0.68 (0.35-1.30, p=0.256) |  |  |
| Unlikely | 207 (86.6) | 32 (13.4) | 3.02 (1.98-4.50, p<0.001) | 1.86 (1.06-3.20, p=0.028) |  |  |
| Quite unlikely | 667 (95.0) | 35 (5.0) | 1.03 (0.69-1.48, p=0.896) | 0.77 (0.47-1.21, p=0.270) |  |  |
| Neither improbable nor probable | 2717 (95.1) | 139 (4.9) | Reference | Reference |  |  |
| Quite likely | 772 (97.1) | 23 (2.9) | 0.58 (0.36-0.89, p=0.018) | 0.67 (0.39-1.12, p=0.137) |  |  |
| Likely | 235 (94.8) | 13 (5.2) | 1.08 (0.58-1.87, p=0.793) | 1.51 (0.72-2.94, p=0.247) |  |  |
| Very likely | 146 (96.1) | 6 (3.9) | 0.80 (0.31-1.70, p=0.607) | 0.50 (0.14-1.48, p=0.244) |  |  |
| **How often you use information from the COVID-19 Hotlines** | | | |  |  |  |
| Never | 2513 (94.0) | 160 (6.0) | 1.18 (0.81-1.79, p=0.404) | 0.96 (0.55-1.71, p=0.887) |  |  |
| Hardly ever | 727 (96.9) | 23 (3.1) | 0.59 (0.34-1.02, p=0.058) | 0.87 (0.44-1.73, p=0.703) |  |  |
| Very little | 583 (95.0) | 31 (5.0) | 0.99 (0.59-1.65, p=0.963) | 1.42 (0.76-2.67, p=0.267) |  |  |
| Some time | 576 (94.9) | 31 (5.1) | Reference | Reference |  |  |
| Often enough | 309 (96.0) | 13 (4.0) | 0.78 (0.39-1.48, p=0.466) | 1.00 (0.45-2.17, p=0.992) |  |  |
| Often | 177 (92.2) | 15 (7.8) | 1.57 (0.81-2.94, p=0.164) | 3.41 (1.42-7.99, p=0.005) |  |  |
| Very often | 96 (95.0) | 5 (5.0) | 0.97 (0.32-2.35, p=0.947) | 1.33 (0.35-4.42, p=0.661) |  |  |
| **Age, years** |  |  |  |  |  |  |
|  | 45.8 (12.9) | 43.8 (13.1) | 0.99 (0.98-1.00, p=0.015) | 0.98 (0.97-1.00, p=0.007) |  |  |
| **Estimate how easy/hard it was to follow recommendations about when to engage in social activities, and when not to?** | | | | | |  |
| Very difficult | 76 (74.5) | 26 (25.5) | 5.00 (2.94-8.33, p<0.001) | 0.56 (0.20-1.49, p=0.249) |  |  |
| Hard | 113 (81.9) | 25 (18.1) | 3.23 (1.92-5.32, p<0.001) | 2.10 (0.95-4.54, p=0.062) |  |  |
| Pretty hard | 414 (90.0) | 46 (10.0) | 1.62 (1.08-2.43, p=0.019) | 1.49 (0.85-2.59, p=0.161) |  |  |
| Neither difficult nor easy | 847 (93.6) | 58 (6.4) | Reference | Reference |  |  |
| Quite easy | 1597 (96.4) | 60 (3.6) | 0.55 (0.38-0.80, p=0.001) | 0.62 (0.37-1.03, p=0.062) |  |  |
| Easy | 1223 (96.5) | 44 (3.5) | 0.53 (0.35-0.78, p=0.002) | 0.64 (0.35-1.17, p=0.147) |  |  |
| Very easy | 711 (97.4) | 19 (2.6) | 0.39 (0.22-0.65, p<0.001) | 1.02 (0.44-2.31, p=0.960) |  |  |
| **Please indicate you general perception during the last 2 weeks: I have felt cheerful and in good spirits** | | | | |  |  |
| All time | 166 (90.7) | 17 (9.3) | 2.41 (1.33-4.18, p=0.002) | 1.39 (0.56-3.32, p=0.467) |  |  |
| Most of the time | 690 (96.0) | 29 (4.0) | 0.99 (0.62-1.56, p=0.962) | 0.69 (0.37-1.27, p=0.238) |  |  |
| More than half the time | 1247 (95.9) | 53 (4.1) | Reference | Reference |  |  |
| Less than half the time | 1225 (93.9) | 79 (6.1) | 1.52 (1.07-2.18, p=0.022) | 1.91 (1.21-3.04, p=0.006) |  |  |
| Some time | 1306 (94.6) | 74 (5.4) | 1.33 (0.93-1.92, p=0.119) | 2.17 (1.30-3.63, p=0.003) |  |  |
| In no time | 347 (93.0) | 26 (7.0) | 1.76 (1.07-2.83, p=0.022) | 3.03 (1.36-6.61, p=0.006) |  |  |
| **Please indicate you general perception during the last 2 weeks: I have felt active and vigorous** | | | | |  |  |
| All time | 207 (91.2) | 20 (8.8) | 1.77 (1.03-2.94, p=0.032) | 0.82 (0.33-1.95, p=0.659) |  |  |
| Most of the time | 714 (94.3) | 43 (5.7) | 1.11 (0.74-1.64, p=0.620) | 1.28 (0.74-2.18, p=0.373) |  |  |
| More than half the time | 1175 (94.8) | 64 (5.2) | Reference | Reference |  |  |
| Less than half the time | 1254 (94.9) | 68 (5.1) | 1.00 (0.70-1.42, p=0.980) | 0.74 (0.47-1.16, p=0.187) |  |  |
| Some time | 1186 (95.1) | 61 (4.9) | 0.94 (0.66-1.35, p=0.755) | 0.81 (0.49-1.35, p=0.421) |  |  |
| In no time | 445 (95.3) | 22 (4.7) | 0.91 (0.54-1.47, p=0.702) | 0.28 (0.12-0.60, p=0.001) |  |  |
| **How often you use COVID-19 related information from the Television** | | | |  |  |  |
| Never | 399 (88.1) | 54 (11.9) | 2.18 (1.50-3.14, p<0.001) | 0.83 (0.47-1.43, p=0.508) |  |  |
| Hardly ever | 343 (95.3) | 17 (4.7) | 0.80 (0.45-1.34, p=0.415) | 0.82 (0.40-1.58, p=0.566) |  |  |
| Very little | 525 (94.6) | 30 (5.4) | 0.92 (0.59-1.41, p=0.712) | 0.69 (0.39-1.19, p=0.190) |  |  |
| Some time | 1209 (94.2) | 75 (5.8) | Reference | Reference |  |  |
| Often enough | 1306 (96.6) | 46 (3.4) | 0.57 (0.39-0.82, p=0.003) | 0.84 (0.53-1.33, p=0.466) |  |  |
| Often | 661 (94.6) | 38 (5.4) | 0.93 (0.61-1.38, p=0.710) | 2.08 (1.24-3.45, p=0.005) |  |  |
| Very often | 538 (96.8) | 18 (3.2) | 0.54 (0.31-0.89, p=0.021) | 1.50 (0.76-2.86, p=0.231) |  |  |
| **Please express whether you approve introduction of curfew from 22:00 to 5:00** | | | |  |  |  |
| Strongly disagree | 403 (81.4) | 92 (18.6) | 2.99 (2.12-4.25, p<0.001) | 1.56 (0.92-2.64, p=0.096) |  |  |
| Disagree | 338 (94.7) | 19 (5.3) | 0.74 (0.42-1.23, p=0.261) | 0.63 (0.32-1.20, p=0.171) |  |  |
| Quite disagree | 459 (95.4) | 22 (4.6) | 0.63 (0.37-1.02, p=0.070) | 0.64 (0.35-1.16, p=0.151) |  |  |
| Neither disagree nor agree | 787 (92.9) | 60 (7.1) | Reference | Reference |  |  |
| Agree enough | 956 (95.5) | 45 (4.5) | 0.62 (0.41-0.92, p=0.018) | 1.02 (0.63-1.65, p=0.938) |  |  |
| Agree | 885 (97.3) | 25 (2.7) | 0.37 (0.23-0.59, p<0.001) | 0.79 (0.44-1.40, p=0.424) |  |  |
| Strongly agree | 1153 (98.7) | 15 (1.3) | 0.17 (0.09-0.29, p<0.001) | 0.53 (0.26-1.02, p=0.063) |  |  |
| **In the last 2 weeks I avoided persons who could infect me, based on their ethnicity** | | | | |  |  |
| No | 3598 (95.1) | 187 (4.9) | Reference | Reference |  |  |
| Yes | 636 (94.2) | 39 (5.8) | 1.18 (0.82-1.66, p=0.361) | 1.60 (1.02-2.49, p=0.038) |  |  |
| Not applicable | 747 (93.5) | 52 (6.5) | 1.34 (0.97-1.83, p=0.071) | 1.09 (0.70-1.67, p=0.702) |  |  |

### S3 Fig. Results of Principal Components analysis.


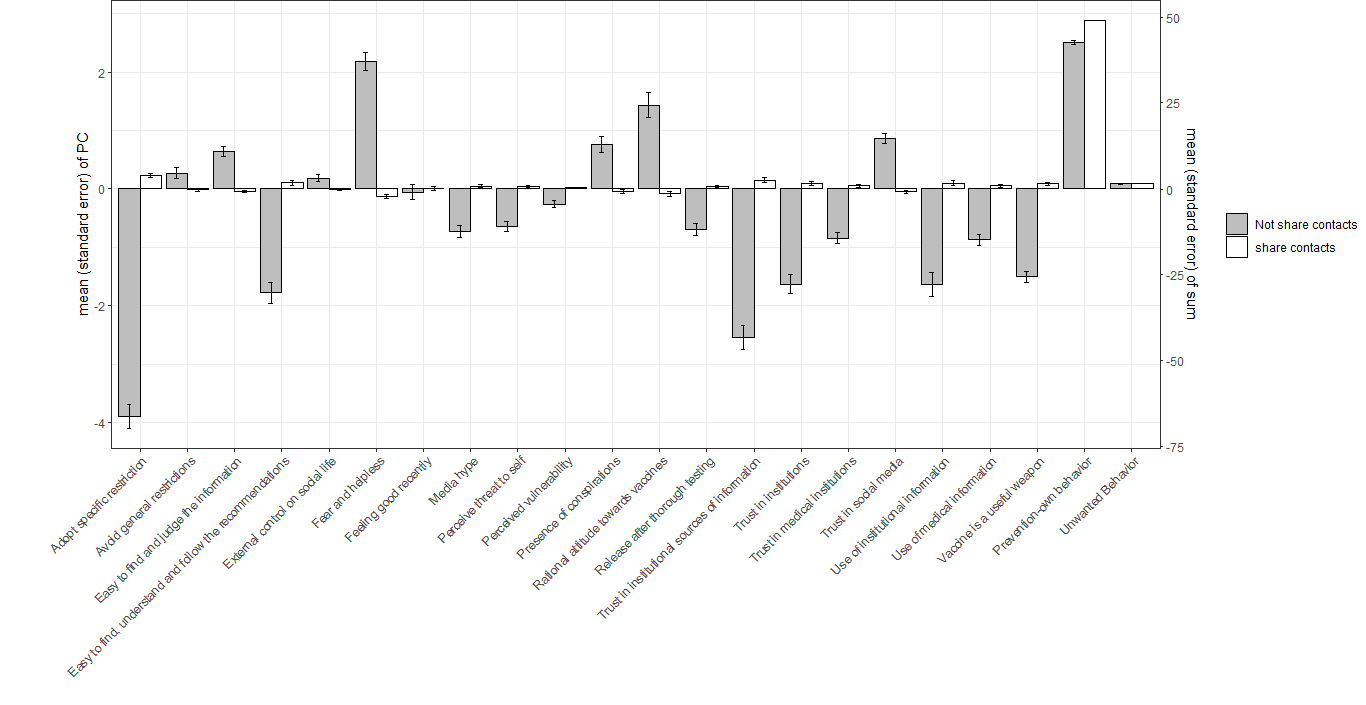


### S4 Table. Principal component proportion of explained variance and their association with unwillingness toward sharing names

|  | Proportion of Variance % | OR (95% CI) adjusted for age, sex, financial situation | Adjusted p-value |
| --- | --- | --- | --- |
| *Health literacy* |  |  |  |
| PC1= Easy to find, understand and follow the recommendations | 60.92685 | 0.858 (0.834-0.883) | <0.0001 |
| PC2= Easy to find and judge the information | 11.81213 | 1.364 (1.275-1.460) | <0.0001 |
| *Probability and Severity + Preparedness and Perceived self-efficacy* |  |  |  |
| PC1= Perceive threat to self | 32.55162 | 0.754 (0.708-0.802) | <0.0001 |
| PC2= Perceived vulnerability | 29.33412 | 0.907 (0.850-0.967) | 0.0031 |
| *Affect* |  |  |  |
| PC1= Fear and helpless | 38.33982 | 1.290 (1.247-1.334) | <0.0001 |
| PC2=Media hype | 19.93765 | 0.840 (0.800-0.880) | <0.0001 |
| *Trust in sources information* |  |  |  |
| PC1= Trust in institutional sources of information | 53.93415 | 0.826 (0.805-0.847) | <0.0001 |
| PC2= Trust in social media | 16.60585 | 1.298 (1.227-1.374) | <0.0001 |
| *Use of sources of information* |  |  |  |
| PC1= Use of institutional information | 46.24551 | 0.876 (0.852-0.900) | <0.0001 |
| PC2= Use of medical information | 14.85224 | 0.803 (0.763-0.845) | <0.0001 |
| *Trust in Institutions* |  |  |  |
| PC1= Trust in institutions | 51.51658 | 0.830 (0.804-0.857) | <0.0001 |
| PC2= Trust in medical institutions | 15.93397 | 0.703 (0.659-0.749) | <0.0001 |
| *Conspiracy* |  |  |  |
| PC1= Presence of conspirations | 64.71986 | 1.124 (1.083-1.168) | <0.0001 |
| PC2= External control on social life | 12.53040 | 1.146 (1.052-1.250) | 0.002 |
| *Lifting restrictions* |  |  |  |
| PC1= Adopt specific restriction | 38.21627 | 0.753 (0.733-0.774) | <0.0001 |
| PC2= Avoid general restrictions | 11.95583 | 1.064 (1.013-1.116) | 0.0125 |
| PC3= Release after thorough testing | 10.32342 | 0.780 (0.735-0.828) | <0.0001 |
| *Wellbeing* |  |  |  |
| PC1= Feeling good recently | 73.12165 | 0.985 (0.946-1.025) | 0.4486 |
| *Covid-19 vaccine* |  |  |  |
| PC1=Rational attitude towards vaccines | 49.38631 | 1.112 (1.084-1.140) | <0.0001 |
| PC2=Vaccine is a useful weapon | 12.37524 | 0.638 (0.603-0.675) | <0.0001 |

### S5 Table. Multivariate logistic model on association between principal components, summation indexes and single questions and unwillingness toward sharing names

|  | OR (95%CI) | p.value |
| --- | --- | --- |

| Age | 0.990 (0.981-0.999) | 0.0366 |
| --- | --- | --- |
| Sex (female) | 1.174 (0.932-1.479) | 0.1737 |
| Financial situation: Remained the same | 0.808 (0.511-1.313) | 0.3747 |
| Financial situation: Worsened | 0.846 (0.524-1.398) | 0.5018 |
| *Principal components* |  |  |
| Easy to find, understand and follow the recommendations | 0.994 (0.955-1.035) | 0.7711 |
| Easy to find and judge the information | 1.195 (1.107-1.292) | <0.0001 |
| Perceive threat to self | 1.045 (0.961-1.136) | 0.3017 |
| Perceived vulnerability | 1.099 (1.000-1.207) | 0.0486 |
| Fear and helpless | 1.052 (1.000-1.107) | 0.0515 |
| Media hype | 0.964 (0.908-1.023) | 0.2288 |
| Trust in institutional sources of information | 1.000 (0.947-1.056) | 0.9866 |
| Trust in social media | 1.019 (0.934-1.113) | 0.6683 |
| Use of institutional information | 0.991 (0.948-1.036) | 0.6788 |
| Use of medical information | 0.899 (0.835-0.967) | 0.0045 |
| Trust in institutions | 0.993 (0.938-1.053) | 0.8241 |
| Trust in medical institutions | 1.042 (0.954-1.138) | 0.3635 |
| Presence of conspirations | 0.997 (0.948-1.049) | 0.9106 |
| External control on social life | 1.056 (0.951-1.174) | 0.3080 |
| Adopt specific restriction | 0.854 (0.816-0.894) | <0.0001 |
| Avoid general restrictions | 1.074 (1.003-1.149) | 0.039 |
| Release after thorough testing | 0.856 (0.799-0.916) | <0.0001 |
| Feeling good recently | 1.034 (0.985-1.086) | 0.175 |
| Rational attitude towards vaccines | 0.989 (0.957-1.021) | 0.493 |
| Vaccine is a useful weapon | 0.944 (0.868-1.028) | 0.1839 |
| *Summation indexes* |  |  |
| Prevention-own behaviour | 0.968 (0.953-0.982) | <0.0001 |
| Unwanted Behaviour | 1.012 (0.938-1.09) | 0.7517 |
| *Original questions* |  |  |
| Have you been infected with COVID-19: Yes | 0.707 (0.434-1.109) | 0.1468 |
| Have you been infected with COVID-19: Don't know | 1.698 (1.221-2.330) | 0.0013 |
| Do you know someone who died from COVID-19: Yes | 0.736 (0.570-0.944) | 0.0169 |
| Frequency of information seeking | 1.003 (0.912-1.102) | 0.9513 |
| It is hard for me to snap back when something bad happens | 0.993 (0.907-1.088) | 0.8797 |
| The decisions that are made in your country to reduce spread of COVID-19 are fair | 0.942 (0.854-1.039) | 0.2349 |
| Apart from COVID-19, I think everyone should be vaccinated according to the national vaccination schedule: Yes | 0.45 (0.328-0.62) | <0.0001 |
| Apart from COVID-19, I think everyone should be vaccinated according to the national vaccination schedule: Don't know | 0.64 (0.461-0.887) | 0.0074 |

|  |  |  |
| --- | --- | --- |

### S6 Table. Multivariate logistic model on association between principal components, summation indexes and single questions significantly related to unwillingness toward sharing names

|  | OR (95%CI) | p-value |
| --- | --- | --- |
| Age | 0.990 (0.981-0.998) | 0.0203 |
| Sex (female) | 1.135 (0.908-1.418) | 0.2660 |
| *Principal components* |  |  |
| Easy to find and judge the information | 1.200 (1.112-1.295) | <0.0001 |
| Use of medical information | 0.901 (0.847-0.959) | 0.0011 |
| Adopt specific restriction | 0.833 (0.803-0.864) | <0.0001 |
| Avoid general restrictions | 1.091 (1.026-1.160) | 0.0056 |
| Release after thorough testing | 0.863 (0.813-0.915) | <0.0001 |
| Vaccine is a useful weapon | 0.931 (0.861-1.006) | 0.0706 |
| *Summation index* |  |  |
| Prevention-own behaviour | 0.966 (0.952-0.980) | <0.0001 |
| *Original questions* |  |  |
| Have you been infected with COVID-19: Yes | 0.757 (0.475-1.165) | 0.2237 |
| Have you been infected with COVID-19: Don't know | 1.747 (1.261-2.388) | 0.0006 |
| Do you know someone who died from COVID-19: Yes | 0.728 (0.567-0.929) | 0.0116 |
| Apart from COVID-19, I think everyone should be vaccinated according to the national vaccination schedule: Yes | 0.447 (0.331-0.607) | <0.0001 |
| Apart from COVID-19, I think everyone should be vaccinated according to the national vaccination schedule: Don't know | 0.649 (0.473-0.889) | 0.0072 |
